# *Leishmania (Leishmania*) *amazonensis* induces macrophage miR-294 and miR-721 expression and modulates infection by targeting NOS2 and L-arginine metabolism

**DOI:** 10.1038/srep44141

**Published:** 2017-03-09

**Authors:** Sandra Marcia Muxel, Maria Fernanda Laranjeira-Silva, Ricardo Andrade Zampieri, Lucile Maria Floeter-Winter

**Affiliations:** 1 Departamento de Fisiologia, Instituto de Biociências, Universidade de São Paulo, São Paulo, SP, Brazil

## Abstract

*Leishmania (Leishmania*) *amazonensis* is an intracellular protozoan parasite responsible for the cutaneous leishmaniasis. The parasite replicates inside mammalian macrophage to establish infection. Host-pathogen interactions result in microRNA-mediated post-transcriptional regulation of host genes involved in inflammatory immune response. We analyzed macrophage miRNA profiles during *L*. (*L*.) *amazonensis* infection. The regulation of macrophage miRNA expression by the parasite correlates with/depends on parasite arginase activity during infection. *L*. (*L*.) *amazonensis (La*-WT) presented significant miRNA profile alteration (27%) compared to *L*. (*L*.) *amazonensis* arginase knockout (*La*-*arg*^−^) (~40%) in relation to uninfected-macrophages. We observed that 78% of the altered miRNAs were up-regulated in *La*-WT infection, while only 32% were up-regulated in *La*-*arg*^−^-infected macrophages. In contrast to *La*-WT, the lack of *L*. (*L*.) *amazonensis* arginase led to the inhibition of miR-294 and miR-721 expression. The expression of miR-294 and miR-721 was recovered to levels similar to *La*-WT in *La*-*arg*^−^ addback mutant. The inhibition of miR-294/*Nos*2 and miR721/*Nos2* interactions increased NOS2 expression and NO production, and reduced *L*. (*L*.) *amazonensis* infectivity, confirming *Nos2* as target of these miRNAs. The role of miR-294 and miR-721 in the regulation of NOS2 expression during *Leishmania* replication in infected macrophages pointing these miRNAs as potential new targets for drug development.

Leishmaniases are diseases caused by protozoan parasites of the genus *Leishmania*. The infection process caused by *Leishmania (Leishmania*) *amazonensis* can generate cutaneous and/or diffuse cutaneous manifestations^1^,^2^. After the inoculum of promastigotes through the sand fly bite, the promastigotes are phagocytized by resident mammalian cells (macrophages, neutrophils, and dendritic cells) and differentiate to amastigotes, the replicative form that establishes infection in the macrophage phagolysosome^1^,^2^. During the initial steps of *Leishmania* infection, monocytes are recruited to the site of infection and initiate the inflammatory immune response with nitric oxide (NO) production^3^. Nevertheless, the parasite survives and replicates inside the macrophages subverting their microbicidal activity and reducing the efficiency of the adaptive immune response^4^.

The cytokines produced during T helper 1 (Th1) responses, such as TNF and IFN-ɣ, and signals transduced via Toll-like receptors (TLRs), induce macrophage nitric oxide synthase 2 (NOS2) expression, resulting in the conversion of L-arginine to NO, which leads to parasite killing^5^,^6^,^7^. On the other hand, Th2 cytokines (IL-4, IL-10, IL-13 and TGF-β) induce macrophage arginase 1 (ARG1) expression, resulting in the conversion of L-arginine into ornithine, a polyamine precursor that promotes the replication and survival of the parasites^8^,^9^,^10^,^11^. Both Th1 and Th2 stimulation induce the expression of the macrophage L-arginine transporter cationic amino acid transporter 2B (CAT2B)^12^. Our group showed that *L*. (*L*.) *amazonensis* encodes its own arginase enzyme^10^,^11^ and also demonstrated that the lack of this protein impairs parasite infectivity^11^. The importance of the parasite L-arginine transporter was also demonstrated, as L-arginine starvation led to increased half-life of one of the transporter transcripts (*La*-AAP3 5.1 mRNA), consequently increased L-arginine uptake^13^. Altogether, these data indicate that L-arginine plays a key role in the survival of *Leishmania* in its mammalian host^5^,^6^,^7^.

Host-pathogen interactions result in signaling and physiological modifications in host cells that induce the microRNA-mediated post-transcriptional regulation of genes involved in the inflammatory response during the induction of the immune response^14^,^15^. miRNAs are non-coding small RNAs that regulate target mRNAs. The interaction of the 21- to 24-nucleotide mature miRNA with the complementary 3′UTR sequence of its target-mRNA blocks the translation of the target mRNA or promotes its degradation^16^,^17^. The miRNAs are transcribed from intergenic, exonic or intronic regions by RNA polymerase II and fold into double-strand primary miRNA transcripts (pri-miRNA)^18^. In the nucleus, class 2 RNAse III DROSHA recognizes the stem-loop structures of pri-miRNA and processes the molecule to form the precursor miRNA transcript (pre-miRNA)^19^ that is exported into the cytoplasm and processed into the mature miRNA by Dicer, another member of the RNAse III family^20^,^21^. The functional strand of the mature miRNA is incorporated into the RNA-induced silencing complex (RISC), which guides the interaction with target mRNA and leads to gene expression regulation^20^,^22^,^23^,^24^.

In recent years, the alteration of miRNA expression by bacteria, viruses and parasites in infectious diseases or other pathologies such as cancer has been studied extensively. Recent studies demonstrated that *L. major* and *L. donovani* infection induce alteration of human and murine host miRNA profiles^25^,^26^,^27^,^28^,^29^. Here, we investigate the role of *L*. (*L*.) *amazonensis* in the regulation of murine host miRNAs. Given the importance of parasite arginase in the establishment of infection through L-arginine metabolism, we evaluate whether this enzyme has a role in the macrophage miRNA profile during infection.

Comparing the expression of 84 miRNAs from macrophages infected with *La*-WT *L*. (*L*.) *amazonensis* with those from macrophages infected with the arginase knockout mutant *La*-*arg*^*−*^, we detected that the lack of arginase promoted a differential regulation of miRNA expression. It is interesting to highlight that while 78% of the altered miRNAs from *La*-WT-infected macrophages were up-regulated only 32% of the altered miRNAs from *La*-*arg*-infected macrophages were up-regulated. Moreover, miR-294 and miR-721 that were up-regulated in the *La*-WT-infection, were down-regulated in *La*-*arg*^*−*^-infected macrophages. We also showed that the absence of parasite arginase led to increased expression of *Nos2* mRNA and the NOS2 protein, with a consequent increase in NO production. The arginase addback presented results similar to *La*-WT-infection. Inhibition of miR-294 and/or miR-721 resulted in an increase in Nos2 and NOS2, with a consequent increase in NO production, confirming the involvement of these miRNAs in a process that lead to reduction of infectivity. Our study demonstrated for the first time the role of miR-294 and miR-721 in the regulation of *Nos2* expression, which is dependent on *Leishmania* arginase and can determine the fate of infection favoring *Leishmania* survival or killing in the host.

## Results

### *L*. (*L*.) *amazonensis* modifies the microRNA profile of infected macrophages

Initially, we validated murine BMDMs as a macrophage model in *La*-WT or *La*-*arg*^*−*^ *L*. (*L*) *amazonensis* infection. As shown in Fig. S1, for both parasites, the course of infection in these macrophages was similar to that described for murine peritoneal macrophages, confirming that *La*-*arg*^−^ impairs infectivity and the replication rate in BALB/c BMDMs^11^.

**Figure 1:**
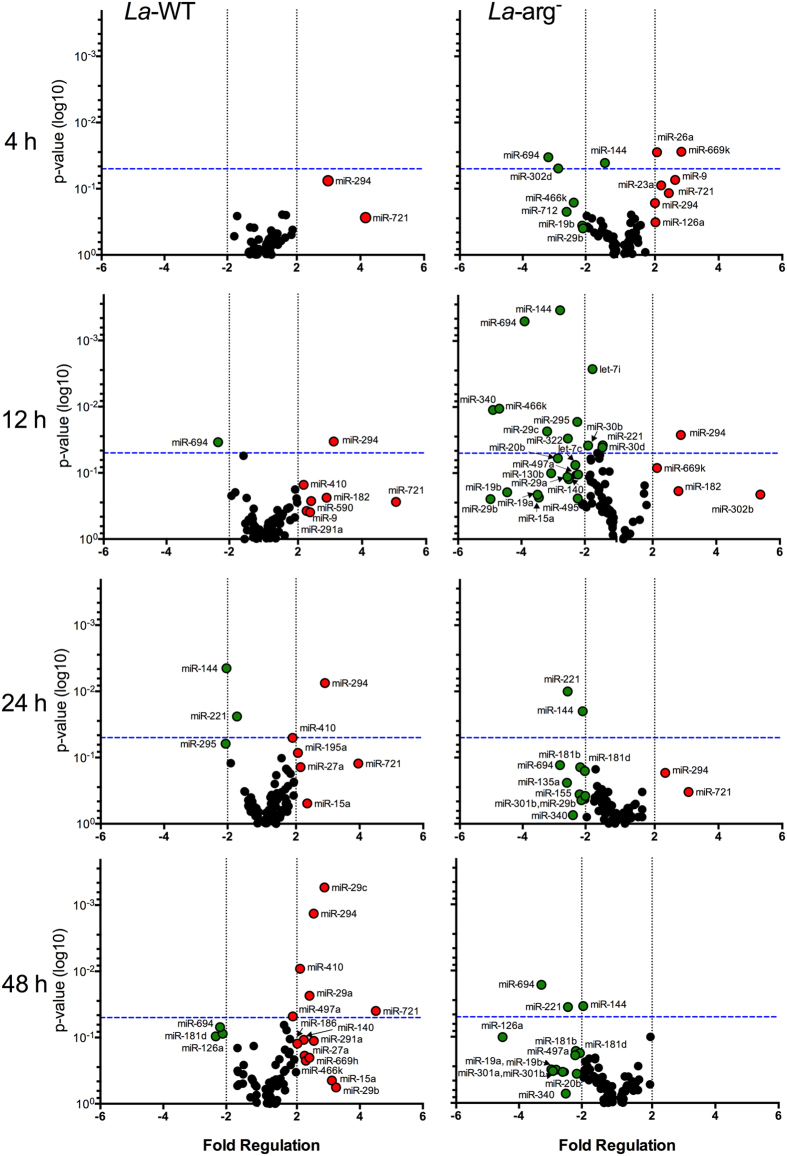
Volcano plot of the miRNA profiles of BMDMs infected with *La*-WT and *La*-*arg*^−^ *L*. (*L*.) *amazonensis*. Each dot represents one miRNA of BMDMs infected for 4, 12, 24 and 48 h with *La*-WT or *La*-*arg*^−^ *L*. (*L*.) *amazonensis*. The red dot indicated up-regulated miRNA and green dot indicated down-regulated miRNAs. Blue dotted line corresponds to p = 0.05, log 10. The relative up- and down-regulation of miRNAs, expressed as boundaries of 2 or −2 of Fold Regulation, respectively. P-value was determined based on two-tailed Student’s t test. The data are representative of three independent experiments.

To determine the role of *La*-WT *L*. (*L*.) *amazonensis* in the miRNA profile of infected murine BMDMs during parasite entrance and replication, we analysed the expression profiles of 84 miRNAs using the miScript Mouse Inflammation miRNA PCR Array with total RNA from BMDMs infected for 4, 12, 24 and 48 h and compared the data with the ones obtained using RNA from uninfected BMDMs kept in culture for the same periods (control group) (Fig. 1, Table S1).

In comparison to uninfected macrophages, the microRNA profiles during parasite entrance (4 h) and replication (12–48 h) revealed the expression regulation of 27% (23/84) of the analyzed miRNAs. Considering Fold Regulation of expression levels greater than or equal to 2.0 as up-regulation and Fold Regulation of expression levels less than or equal to −2.0 as down-regulation, of these 24 miRNAs, 78% (18/23, red dot) were up-regulated and 22% (5/23, green dot) were down-regulated (Fig. 1, Table S1).

The up-regulation of miR-294-3p and miR-721 was detected since the start of the infection (4 h) (Fig. 1, red dot) and was sustained during the replication phase (12–48 h of infection) in comparison to uninfected (red dot, Fig. 1, Table S1). The increase in the expression levels of miR-294-3p was significant from 12 to 48 h and of miR-721 at 48 h. After 12 h of infection, we observed the up-regulation of the following group of miRNAs: miR-182-5p, miR-291a-3p, miR-410-3p, miR-590-3p and miR9-5p. Moreover, miR-291a-3p, miR410-3p up-regulation was sustained from 12 to 48 h of infection. After 24 h, the expression levels of miR-15a-5p, miR-195a-5p, miR-27a-3p were increased and sustained until 48 h of infection (red dot, Fig. 1, Table S1). Yet, the expression levels of miR-140-5p, miR-186-5p, miR-29a-3p (p < 0.05), miR-29b-3p, miR-29c-3p (p < 0.01), miR-466k, miR-497a-5p (p < 0.05) and miR-669h-3p were higher than in uninfected macrophages. We also observed the down-regulation of miR-694 after 12 to 48 h of infection. Others miRNAs down-regulated after 24 and 48 h of infection were: miR-126-5p, miR-144-3p, miR-181d-5p, miR-295-3p (green dot, Fig. 1, Table S1). Thus, our results show that *L*. (*L*.) *amazonensis* is able to modify the macrophage miRNA expression profile during its entrance and replication.

### Lack of *L*. (*L*.) *amazonensis* arginase leads to distinct regulation of miRNA profile of infected macrophages

Analyses of the 84 miRNAs from total RNA of *La*-*arg*^−^-infected macrophages revealed a distinct dysregulation of the miRNA profile of BMDMs (Fig. 1, Table S2). It is remarkable that 40% of miRNAs was regulated (34 of the 84). However, only 32% (11/34, red dot) of the miRNAs were up-regulated; most of the miRNAs (68%, green dot) were down-regulated when compared to the *La*-WT-infected profile (Fig. 1, Table S1).

After 4 h of *La*-*arg*^−^ infection, the expression levels of miR-294-3p and miR-721 were increased, but the Fold Regulation values were lower than in *La*-WT-infected macrophages. The miR-294-3p expression at 12 h in *La*-*arg*^−^-infected macrophage was similar to *La*-WT-infected macrophage. In contrast, the expression level of miR-294-3p at 24 and 48 h was lower in *La*-*arg*^−^-infected macrophage compared to *La*-WT-infected. At 12 and 48 h, we were not able to detect any expression of miR-721 in *La*-*arg*^−^-infected macrophage, the expression was detected only at 24 h of infection.

The infection with *La*-*arg*^−^ led to increased expression of miR-126a-5p, miR-182-5p, miR-23a-3p, miR-26a-5p, miR-302b-3p, miR-669k-3p and miR-9-5p. Additionally, compared with *La*-WT infection, infection with *La*-*arg*^*−*^ led to down-regulation of the following miRNAs at 4–24 h: let-7b-5p, let-7c-5p, miR-130b-3p, miR-135a-5p, miR-140-5p, miR-155-5p, miR-15a-5p, miR-181b-5p, miR-19a-3p, miR-19b-3p, miR-20b-5p, miR-221-3p, miR-29a-3p, miR-29b-3p, miR-29c-3p, miR-30b-5p, miR-301a-3p, miR-301b-3p, miR-302d-3p, miR-322-5p, miR-340-5p, miR-466k, miR-495-3p, miR-497a-5p, and miR-712-5p; and miR-126a-5p was down-regulated after 48 h of infection. These results show the importance of *L*. (*L*.) *amazonensis* arginase in determining the macrophage miRNA profile during infection and suggest a new role for parasite arginase in the modulation of the macrophage immune response to *Leishmania*.

### NOS2 expression and NO production are up-regulated during infection with *La*-arg^−^ *L*. (*L*.) *amazonensis*

**Figure 2:**
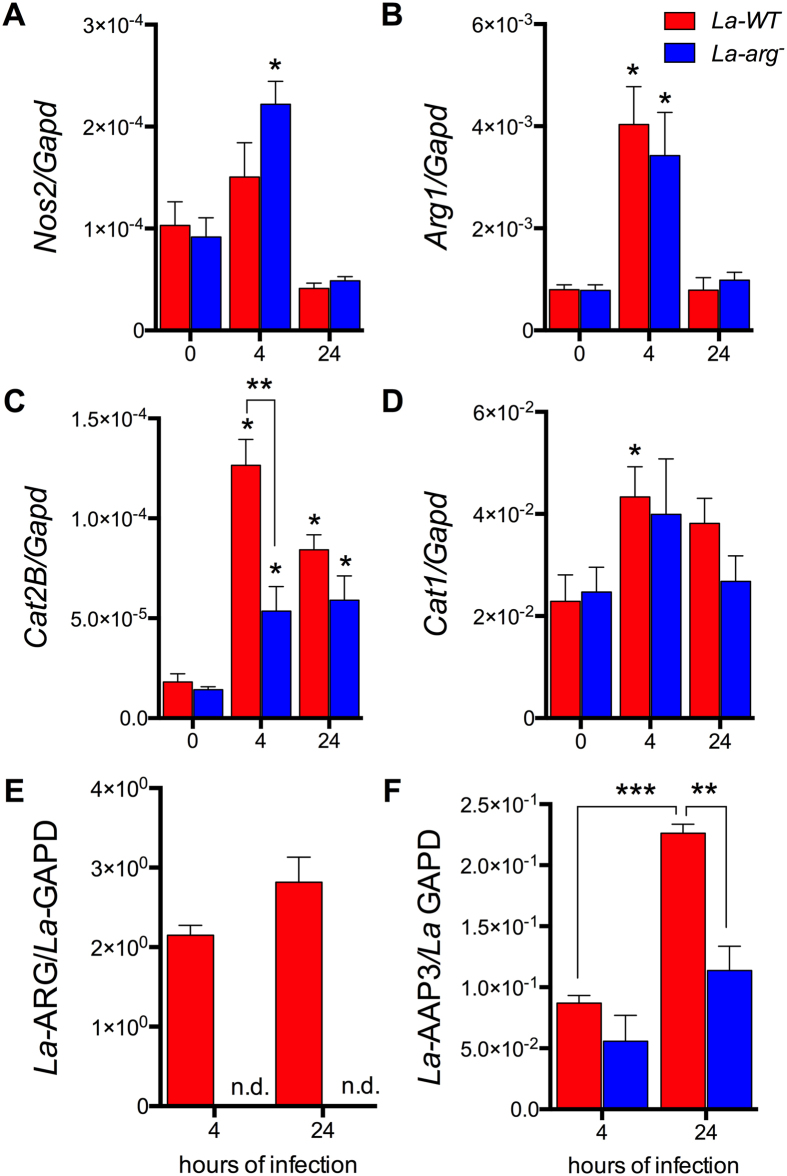
Lack of *L*. (*L*.) *amazonensis* arginase leads to differential modulation of expression of genes involved in the polyamine and NO production pathways. The BMDMs (1 × 10^6^) were infected with *La*-WT (red) or *La*-*arg*^*−*^ (blue) *L*. (*L*.) (MOI 5:1). After 4 and 24 h of infection, the copy numbers of mRNAs of *Nos2* (**A**), *Arg1* (**B**), *Cat2B* (**C**), and *Cat1* (**D**) from macrophages and *La*-*ARG* (**E**) and the *La*-*AAP3 5.1* (**F**) from *Leishmania* were quantified by real-time RT-qPCR. Each bar represents the average ± SEM of the values obtained in 3 independent experiments (n = 6). Statistical significance was determined based on two-tailed Student’s t test. *p < 0.05, compared to uninfected macrophages (0 hours of infection). **p < 0.05, *La*-WT compared to *La*-*arg*^−^. n.d. (not detected).

**Figure 3:**
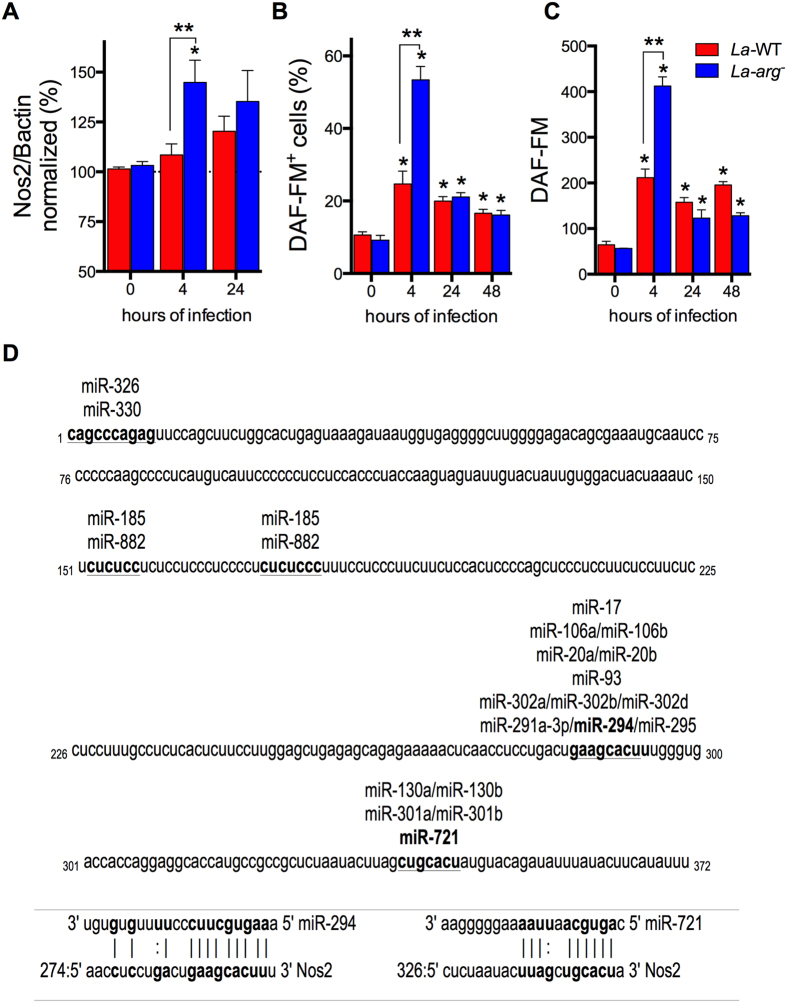
NOS2 protein amount and NO production in BMDMs infected with *La*-WT and *La*-*arg*^*−*^ *L*. (*L*.) *amazonensis*. BMDMs (1 × 10^6^) were infected with *La*-WT (red) or *La*-*arg*^−^ (blue) *L*. (*L*.) *amazonensis* (MOI 5:1). After 4 and 24 h of infection, the protein levels of NOS2 (**A**), the frequency of cells producing NO (**B**) and the average of fluorescence intensity reflecting NO production (**C**) were determined by *in*-*cell* western blotting and flow cytometry analysis of DAF-FM staining. Each bar represents the average ± SEM of the values obtained in 3 independent experiments, n = 6. Statistical significance was determined based on two-tailed Student’s t test. *p < 0.05, compared to uninfected macrophages (0 hours of infection). **p < 0.05, *La*-WT compared to *La*-*arg*^*−*^. (**D**) Sequence alignment of miR-294 and miR-721 and its target site in the 3′UTR of Nos2 using the microRNA.org database. Mmu-miR-294/Nos2 Alignment: mirSVR score −0.3992, PhastCons: 0.5607; Mmu-miR-721/Nos2 Alignment: mirSVR score −0.1183, PhastCons: 0.6227. Target sites of conserved miRNAs with good mirSVR scores.

The pivotal use of L-arginine in the polyamine pathway or in NO production via NOS2 activation can determine the fate of the parasite during infection. After 4 h of infection with *La*-WT *L*. (*L*.) *amazonensis*, the host *Nos*2 expression was not altered (Fig. 2A), but we observed up-regulated expression of host *Arg*1 mRNA (Fig. 2B). We also observed up-regulated expression of macrophage L-arginine transporter *Cat2B* after 4 and 24 h of *La*-WT-infection, but did not *Cat1* (Fig. 2C and D). The *Leishmania* arginase transcript was detected after 4 and 24 h of *La*-WT infection (Fig. 2E). L-arginine transporter AAP3 (*La*-AAP3 5.1) transcript was increased after 24 h of infection in comparison to the 4 h time point (Fig. 2F). The lack of arginase in *L*. (*L*.) *amazonensis* led to a similar up-regulation of the host *Nos*2 and *Cat1* mRNAs (Fig. 2A and D), but *Cat2B* expression was reduced after 4 h of infection in comparison to uninfected macrophages (Fig. 2C). Interestingly, the expression of *Arg*1 during *La*-*arg*^−^ infection was similar to the one observed during *La*-WT infection (Fig. 2B). After 24 h of *La*-*arg*^*−*^ infection, the expression of the *La*-AAP3 5.1 was lower than the one observed for *La*-WT infection (Fig. 2F). As expected, parasite arginase mRNA was not detected in *La*-*arg*^*−*^ infection (Fig. 2E). The higher levels of *Nos2* mRNA detected during *La*-*arg*^*−*^ infection led to an increased NOS2 expression (Fig. 3A) and NO production after 4 h of infection, which was determined by the increased percentage of cells producing NO (DAF-FM^+^ cells, Fig. 3B) and by the amount of NO produced per cell (DAF-FM florescence intensity mean, Fig. 3C). These results indicate that arginase from *L*. (*L*.) *amazonensis* alter the expression of host polyamine/NO production pathways, favoring parasite replication and survival in the host.

### *In silico* search of mRNA/miRNA interactions

For the *in silico* search of the miRWalk and microRNA.org databases to predict miRNA/mRNA interactions, we focused on L-arginine metabolism. This approach elucidates whether the differentially regulated miRNAs (miR-291a-3p, miR-294-3p, miR-29a-3p, miR-29c-3p, miR-410-3p, miR497a and miR-721, Fig. 1) are involved in the gene expression mediated by *L*. (*L*.) *amazonensis* arginase. The predicted targets of these miRNAs are the genes from the L-arginine-nitric oxide (NO) pathway (*Cat2, Cat1, Arg1* and *Nos2*) (Fig. S2). The *in silico* analysis showed that miR-294-3p and miR-721 had the same predicted targets, such as *Nos2, Il6*, besides pathways involved in phagolysosome formation (LAMP-1 or LAMP-2) (Table S3 and S4). These predictions can be taken as evidence of L-arginine-NO pathway regulation by miRNAs during infection with *La*-WT *L*. (*L*.) *amazonensis* and are corroborated by the data obtained during *La*-*arg*^*−*^ infection, such as the increased *Nos2* mRNA expression (Fig. 2A), NOS2 (Fig. 3A) and NO production (Fig. 3B and C) after 4 h of infection.

After performing the *in silico* search for miRNA targets, we looked specifically for miRNA candidates that could bind to the 3′UTR of *Nos2* mRNA from *Mus musculus* in miRanda – miRSVR database (microRNA.org) (Fig. 3D). The analysis showed the putative binding of differentially regulated miRNAs from macrophages infected with *La*-WT or *La*-*arg*^*−*^, such as miR-291a-3p, miR-294 and miR-721 that were up-regulated (Figs 1 and 3D). In addition, the putative binding site of miR-291/miR-294 is also complementary to miR-302a, miR-302b or miR-302d and potentially could be amplified in the miRNA qPCR array we used (Table S1 and S2). However, only miR-302b-3p was increased at 12 h of infection with *La*-*arg*^*−*^, but the level of expression was reduced in the longer periods of infection (Fig. 1). Moreover, the putative binding site of miR-721 is also complementary to miR-130a/miR-130b, miR-301a and miR-301b, but these miRNAs were less detected in *La*-WT infection and were down-regulated in *La*-*arg*^*−*^ (Figs 1 and 3D, Table S1 and S2).

Previous studies showed that miR-291a and miR-294 belong to the same family and are regulators of the cell cycle during embryogenesis^30^,^31^,^32^. These miRNAs are processed from the same encoded hairpin cluster^30^ (GenBank Gene ID: 100049712). The miR-721 is transcribed from the intronic region of the CUX-1 gene (GenBank Gene ID: 13047), which encodes a tumor suppressor protein^33^. The miR-721 was identified in embryogenic development and was specifically expressed in the nervous system^34^. However, the validation of targets for these miRNAs has not been described in the literature. Using the microRNA.org database, we showed the predicted interactions of Mmu-miR-294p/*Nos2* and Mmu-miR-721/*Nos2* mRNA, with good alignment mirSVR (−0.3992 and −0.1183, respectively) and PhastCons (0.6227 and 0.5607, respectively) scores for the predicted mRNA/miRNA interactions (Fig. 3D).

Our data indicate that the interaction of miR-294 and miR-721 to the 3′UTR of *Nos2* can lead to the down-regulation of *Nos2* translation and NO production during *L*. (*L*.) *amazonensis* infection and also indicates that the absence of *Leishmania* arginase impairs miR-294 and miR-721 expression favoring *Nos2* translation.

### *L*. (*L*.) *amazonensis La*-arg^−^ addback recovered the WT profile of miR-294-3p and miR-721 and *Nos*2 expression

For confirm the importance of *La*-arginase for *Nos*2 expression and its implications in the miR-294-3p and miR-721 expression during infection, we performed experiments with *L*. (*L*.) *amazonensis arg*^−^ addback arginase (*La*-*arg*^−^ + ARG). The *La*-*arg*^−^ + ARG presented a low activity of arginase but similar infectivity compared to *La*-WT^35^.

**Figure 4:**
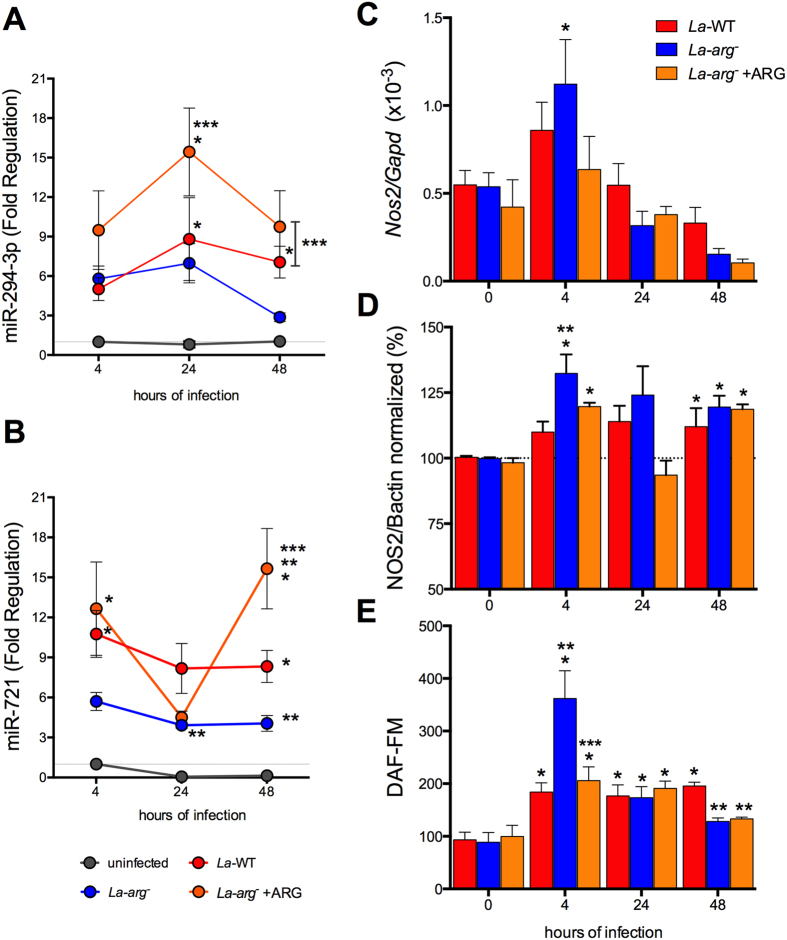
Levels of miR-294-3p, miR-721, *Nos2* mRNA, NOS2 and NO in BMDMs infected with *La*-WT, *La*-*arg*^*−*^ or *La*-*arg*^−^ + ARG *L*. (*L*.) *amazonensis*. BMDMs (1 × 10^6^) were infected with *La*-WT (red) or *La*-*arg*^−^ (blue) or *La*-*arg*^−^ + ARG (orange) *L*. (*L*.) *amazonensis* (MOI 5:1). After 4, 24 and 48 h of infection, miR-294-3p (**A**), miR-721 (**B**) and *Nos2* mRNA levels were quantified via qPCR (**C**), NOS2 protein levels was quantified via in-cell western blotting (**D**) and the average of fluorescence intensity reflecting NO production (**E**) was determined by flow cytometry analysis of DAF-FM staining and values was normalized by uninfected macrophages (100%). Each bar or dot represents the average ± SEM of the values obtained in 3 independent experiments (n = 4–6). Statistical significance was determined based on two-tailed Student’s t test. *p < 0.05, compared to uninfected macrophages (0 hours of infection). **p < 0.05, *La*-*arg*^−^ or *La*-*arg*^−^ + ARG compared to *La*-WT. ***p < 0.05, *La*-WT or *La*-*arg*^−^ + ARG compared to *La*-*arg*^−^.

The levels of miR-294-3p and miR-721 determined for BMDMs macrophages infected with the arginase addback confirmed their participation in *Nos2* regulation (Fig. 4A and B). The *La*-*arg*^−^ + ARG infection increased the expression levels of miR-294-3p and miR-721 at 4–48 h of infection, compared to uninfected macrophages, to levels similar to the observed in *La*-WT infection (Fig. 4A and B). Indeed, the levels of expression of miR-294-3p (24–48 h) and miR-721 (48 h) were significant higher compared to *La*-*arg*^−^ infections. As previously observed (Fig. 1), the expression of miR-294-3p and miR-721 was increased in *La*-WT compared to uninfected. As expected, the levels of miR-294-3p and miR-721 not modified in uninfected macrophages during different times of incubation (Fig. 4A and B).

The *Nos*2 mRNA expression and the NOS2 protein levels in *La*-*arg*^−^ + ARG were similar to *La*-WT, at 4 and 48 h of infection (Fig. 4C and D). The levels of *Arg*1 mRNA in the *La*-*arg*^−^ + ARG were similar to *La*-WT (Fig. S3). The amount of NO produced per cell was similar to *La*-WT after 4–24 h and decreased after 48 h of infection, but these levels was lower compared to *La*-*arg*^−^ infection, after 4 h (Fig. 4E).

These results support that parasite arginase could indirectly modulate the levels of *Nos*2-NOS2 and NO production via miRNAs induction besides competing for L-arginine substrate.

### Block miR-294/*Nos2* and miR-721/*Nos2* interactions induces NOS2 expression and impairs *L*. (*L*.) *amazonensis* infection

**Figure 5:**
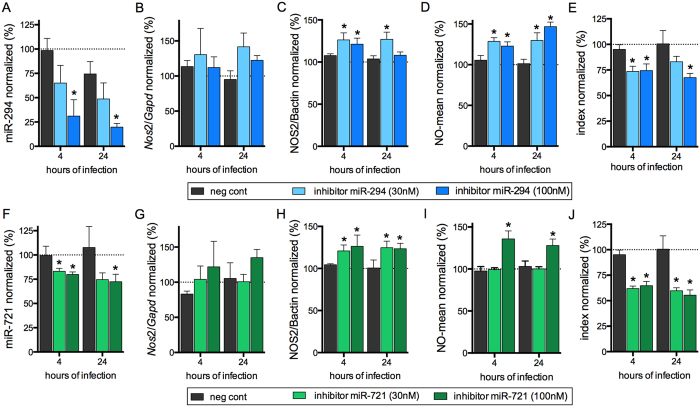
Inhibition of miR-294 and miR-721 functions increased NO production and reduced the infectivity of *L*. (*L*.) *amazonensis*. BMDMs (5 × 10^5^) were transiently transfected with the negative control, 30 or 100 nM of miR-294-5p (**A**–**E**) or miR-721 (**F**–**J**) inhibitors, or left non-transfected (untreated, black bars). After 24 h of incubation, the cells were co-cultivated with *La*-WT *L*. (*L*.) *amazonensis* (MOI 5:1) for 4 h, and the cultures were then washed. After 4 and 24 h of infection, the samples were analyzed for miR-294 (**A**), miR-721 (**F**) and *Nos2* mRNA (**B**,**G**) levels via RT-qPCR of total RNA, for NOS2 expression (**C**,**H**) via in-cell western, for NO production (**D**,**I**) via flow cytometry DAF-FM fluorescence analysis, and for infectivity (**E**,**J**) via microscopy analysis, counting of the numbers of infected macrophages and amastigotes per macrophage (n = 1,000 macrophages/treatment). The values were normalized based on the average values of untreated infected macrophages. Each bar represents the average ± SEM of the values obtained in 3 independent experiments (n = 4–6). Statistical significance was determined based on two-tailed Student’s t test. *p < 0.05, compared to negative control infected macrophages.

BMDMs transfected with 30 nM and 100 nM of miR-294 or miR-721 inhibitors showed respectively 40% and 70% inhibition of miR-294 expression after 4 hours of infection (Fig. 5A). This inhibition was observed for up to 24 h of infection. The reduction in miR-294 expression did not promote a statistically significant change in the amount of *Nos*2 mRNA (Fig. 5B), even so NOS2 expression (Fig. 5C) and NO production was increased after 4 and 24 h of infection (Fig. 5D), parallel to a 25–30% decrease in infectivity (Fig. 5E).

We also observed a much weaker inhibition effect for miR-721 than the one observed for miR-294. The miR-721 inhibition levels were 20% and 30% with 30 or 100 nM of inhibitor, respectively (Fig. 5F). In these conditions, *Nos2* mRNA was not significantly modified after 4 and 24 h of infection (Fig. 5G), but NOS2 expression (Fig. 5H) and NO production (Fig. 5I) were increased parallel to a decreased infectivity after 4 and 24 h of infection (Fig. 5J). These data indicate a role of miR-294 and miR-721 in the post-transcriptional regulation of *Nos*2 mRNA in macrophages infected with *L*. (*L*.) *amazonensis*.

**Figure 6:**
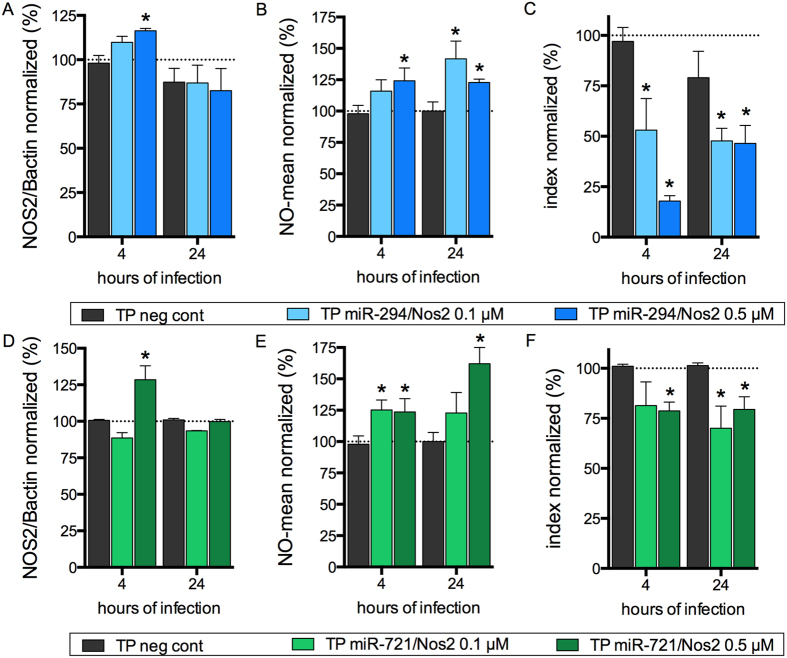
miR-294 and miR-721 bind to Nos2 mRNA and regulating NOS2 expression and *L*. (*L*.) *amazonensis* infectivity. BMDMs (5 × 10^5^) were plated into chamber slides overnight and transfected with negative control, 0.1 or 0.5 μM miScript Target Protector for miR-294/*Nos2* or miR-721/*Nos*2 or negative control for 24 h. Then, the BMDMs were co-cultivated with *La*-WT *L*. (*L*.) *amazonensis* (MOI 5:1) for 4 h, and the cultures were washed. After 4 h and 24 h of infection, the samples were analyzed for NOS2 protein levels (**A**,**D**) via in-cell western, NO production (**B**,**E**) via flow cytometry DAF-FM fluorescence analysis, and for infectivity (**C**,**F**) via microscopy analysis, counting of the numbers of infected macrophages and amastigotes per macrophage (n = 1,000 macrophages/treatment). The values were normalized based on the average of untreated infected macrophages. Each bar represents the average ± SEM of the values obtained in 3 independent experiments (n = 4–6). Statistical significance was determined based on two-tailed Student’s t test. *p < 0.05, compared to negative control infected macrophages.

The miScript Target Protector (Qiagen) competition assay for the binding site of miR-294 to the *Nos2* 3′UTR using 0.1 μM or 0.5 μM miR-294/*Nos2* revealed a significant increase (20% to 22%) in the levels of NOS2 after 4 h of infection (Fig. 6A). The competition led to a 20% to 25% increase in NO production after 4 h. After 24 h, competition with 0.1 μM led to a 40% increase in NO production (Fig. 6B), while with 0.5 μM NO production increased 20%. With both concentrations, the increased NO production led to an infectivity reduction of at least 50% after 4 h or 24 h, reaching 80% with 0.5 μM after 4 h (Fig. 6C).

Competition with 0.5 μM miR-721/Nos2 Target Protector led to a 25% increase in NOS2 protein levels only after 4 h of infection, and competition with 0.1 μM had no effect after 4 and 24 h of infection (Fig. 6D). Nonetheless, competition with both concentrations resulted in increased NO production (25%) after 4 h. This NO production increase was sustained with 0.1 μM after 24 h, but competition with 0.5 μM led to a 60% increase in NO production (Fig. 6E). This observed NO production increase led to a 25% decrease of *L*. (*L*.) *amazonensis* infectivity after 4 and 24 h of infection (Fig. 6F).

Our results indicate that competition for the miR-294 or miR-721 binding site in the 3′UTR of *Nos2* mRNA reversed NOS2 down-regulation and induced NO production, with a consequent impairment of *Leishmania* survival in macrophages.

**Figure 7:**
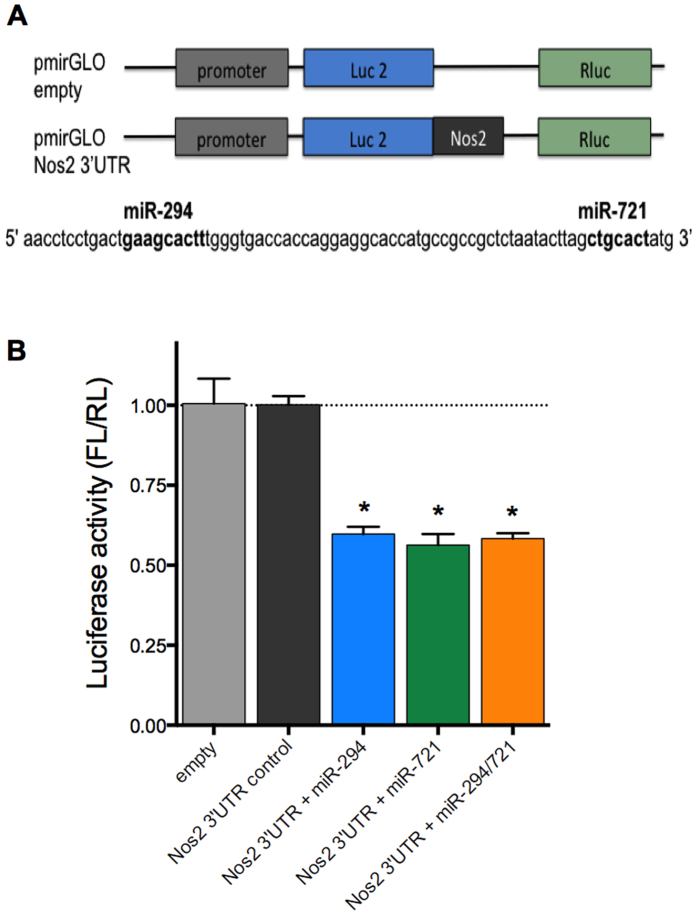
miR-294 and miR-721 bind to *Nos2* 3′UTR. RAW 264.7 (5 × 10^5^) were transfected with 5 μg of pmiRGLO, empty or with the regions containing miR-294 or miR-721 binding sites to *Nos2* 3′UTR, plus 100 nM of miR-294 or/and miR-721 mimics (**A**). After 24 h, the cells lysates were analyzed for luciferase activity (**B**). The values of firefly luciferase (FL) were firstly normalized by renilla luciferase (RL), and then normalized by the values obtained with cells transfected with pmiRGLO empty construct. Each bar represents the average ± SEM of the values obtained in 3 independent experiments (n = 5). Statistical significance was determined based on two-tailed Student’s t test. *p < 0.05, compared to pmiRGLO empty vector.

To confirm the interaction of miR-294 and miR-721 with *Nos2* 3′UTR, BMDMs were transfected with pmiRGLO constructs containing the regions of miR-294 and miR-721 interactions in *Nos2* 3′UTR (Fig. 7A). After 24 h, cells were assayed for dual luciferase activity. The transfection with 100 nM of miR-294 and/or miR-721 mimic resulted in a 40% inhibition of firefly luciferase (FL) (Fig. 7B) confirming the interaction of these miR with *Nos2* 3′UTR.

## Discussion

It has been well documented that L-arginine plays a dual role in the pathogenesis of *Leishmania* infection^36^,^37^. This amino acid influences the survival of the parasite via polyamines production by both *Leishmania (La*-*arginase*) and macrophage (ARG1) arginases^10^,^11^,^36^,^38^. On the other hand, L-arginine is also implicated in NO production through NOS2 activity to trigger the macrophage inflammatory response and kill the parasites^36^. Here, we showed that *L*. (*L*.) *amazonensis* infection alters murine macrophage miRNA profiles, adjusting the balance between ARG1 and NOS2, favoring ARG1 activity and parasite survival.

We focused our analyses on L-arginine metabolism, since macrophages respond to cytokines produced during a Th1 response, such as IFN-γ, IL-1β, TNF-α, by inducing the expression of CAT2B and NOS2^5^,^6^,^39^, leading to the conversion of L-arginine into NO and parasite killing^5^,^6^,^7^. On the other hand, the stimulation of macrophages with Th2 cytokines (IL-4, IL-10, IL-13 e TGF-β) induces CAT2B and ARG1 expression leading to polyamine production and parasite survival^12^.

*L*. (*L*.) *amazonensis* also possesses the machinery to uptake (L-arginine transporter – *La*-AAP3)^13^ and metabolize L-arginine to polyamines (arginase)^10^,^11^. Parasite arginase can compete for macrophage arginine, subverting NO production by inducing ARG1, probably in a coordinated action to produce polyamines. *Leishmania* can sense L-arginine availability in the environment and induce the expression of the L-arginine transporter *L*. (*L*.) *amazonensis (La*-AAP3)^13^ and *L. donovani (Ld*-AAP3)^40^,^41^, increasing the rate of L-arginine uptake^13^,^40^. *L. donovani* amastigotes express the Ld-AAP3 transporter and compete for L-arginine from the host phagolysosome^42^.

The data presented here confirmed that *L*. (*L*.) *amazonensis* infection induces the expression of both macrophage and parasite L-arginine transporters, CAT2B, CAT1 and *La*-AAP3, and arginases, ARG1 and *La*-arginase. Nevertheless, the levels of La-AAP3 5.1 (5,5 × 10^−1^ ± 0.05) and La-ARG (13 ± 1.0) in stationary-phase promastigotes were higher than in amastigotes from WT-infected macrophage (4–24 h)^13^,^35^. Amino acid deprivation repress the pol II transcription of CAT-1 mRNA in hepatocarcinoma (Huh7) cells, but CAT-1 protein level increased probably mediated by binding of HuR (ARE binding protein) to the 3′UTR of CAT-1 mRNA, blocking miR-122-induced inhibition and its release from cytoplasmic processing bodies (P-bodies) and its recruitment to polysomes^43^. During infection with *L. donovani* gp63 cleaves Dicer1, that could impact in post-transcriptional regulation of host-mRNAs by miRNA/RNP interactions, as showed by Dicer1-mediated inhibiting of pre-miR-122 processing and downregulation of miR-122 activity in Huh7 cells^28^.

However, the absence of *Leishmania* arginase promoted a distinct modulation of mRNA expression, with up-regulation of NOS2 and lower increase of CAT2B, CAT-1 and *La*-AAP3. This decrease in CAT2B expression reduced the infectivity of *L*. (*L*.) *amazonensis*, probably by reducing the uptake of L-arginine and polyamine production, then supplementation with putrescine allow the infectivity^44^. Indeed, the deprivation of L-arginine decreases ornithine and putrescine levels but does not change the levels of spermidine, spermidine or agmatine in promastigotes^45^. Nevertheless, the absence of *Leishmania* arginase increases L-arginine and citrulline levels but decreases the levels of ornithine, putrescine and proline in promastigotes, indicating an induction of alternative pathway to surpass the enzyme or its substrate absence^13^,^35^,^45^. Here we show that similar to *La*-WT, the *Leishmania* arginase addback led to reduction of *Nos2* mRNA and NOS2 protein levels and increase of *Arg*1 levels, consequently reducing NO production. These observations support the idea that reducing/blocking parasite arginase activity shifts L-arginine pathway to produce citrulline and NO, improving the host capacity to control the infection.

*L*. (*L*.) *amazonensis* WT infection alters the levels of 27% of 84 macrophage miRNAs that were analyzed at different time points of the infection. Of these miRNAs, 78% were up-regulated. This observation is consistent with the available data for *Leishmania major* (MHOM/TN/95/GLC94 strain) infection, which modulated approximately 20% of the miRNAs of infected human monocyte-derived macrophages (MDM), 70% of which were up-regulated^27^, and *L. donovani*-infected mouse liver^28^. Despite the technique used to screen miRNA dysregulation, *L. major* (MHOM/IL/80/Friedlin) and *Leishmania donovani* (MHOM/SD/00/1s) also promoted modifications in miRNA expression in infected-MDMs^29^. Similarly was observed for *L. major* (MHOM/IL/81/FE/BNI) infection of BMDMs from BALB/c mice^25^ or infection of peritoneal macrophages from BALB/c with *L. donovani* resistant to antimony^26^. Further, considering genetic differences, the states of activation of the tested macrophages and differences among *Leishmania* species, these studies supported the proposition that *Leishmania* infection can modify the expression of macrophage miRNAs to alter regulatory RNA function in host-cells promoting the evasion from the host immune response.

Interestingly, the comparison of macrophage miRNAs profiles showed an opposite effect during *La*-*arg*^−^ infection. In contrast to the observed with *La*-WT infection, a negative regulation of genes involved in L-arginine metabolism was observed, as shown in a schematic model based on predicted miRNA/mRNA interactions (Fig. S2). *L. amazonensis* upregulated miR-29a, miR-29b and miR29c, after 12–48 h of infection, the absence of La-arginase downregulate these miRNAs. Despite, *L. major* upregulates miR-29b of human MDMs, the *L. donovani* downregulates miR-29a and miR-29b, but both did not modulate miR-29c^29^. The miR-29 was upregulated mediated by NOD2-stimulation, that downregulates IL-12p40, but not alter IL-6, TGF-β or IL-10 levels^51^. However, NOD2-stimulation amplifying the release of IL-1β, IL-6, and IL-23^49^,^50^ and induces NOS2 expression and NO production^41^,^42^. Among the dysregulated miRNAs, miR-294-3p and miR-721 drew our attention, as these miRNAs were less regulated in *La*-*arg*^*−*^-infected macrophages and were predicted as candidates for regulating the expression of proteins involved in the L-arginine pathway. In fact, the infection with *La*-*arg*^*−*^ resulted in lower levels of miR-294-3p and miR-721, which can be associated with higher levels of *Nos2* mRNA, NOS2 and NO production in infected macrophages, all associated with a lower infectivity. The infection of macrophages with arginase addback promotes a similar phenotype of *La*-WT, inducing high levels of miR-294-3p and miR-721 and low levels of *Nos2* mRNA, NOS2 and NO production. These observations elucidate the importance of parasite arginase for the balance of polyamine versus NO production and the maintenance of the infection. The absence of arginase in *L. mexicana* also reduced infectivity in a NOS2-NO-dependent manner^46^. Moreover, the control of *L. major* infection depends on the ability of macrophages to induce NOS2-NO^47^. Although L-arginine availability plays a key role in the survival of *Leishmania* in the mammalian host^5^,^6^,^7^, a negative modulation of the uptake of L-arginine by CAT2B can also reduce the infectivity of *L*. (*L*.) *amazonensis*^44^.

*Nos2* expression can be regulated at the transcriptional level by factors such as NF-*k*B^48^, Oct-1^49^ and STAT^50^. Indeed, *L. major* and *L. amazonensis* infections increase the nuclear translocation of the NF-kB p50/p50 dimer, which correlate with the decrease of *Nos2* and *Cat2B* transcription levels^12^. The accumulation of *Nos2* mRNA and NOS2 during *La*-*arg*^−^ infection suggested that miR-294-3p and miR-721 act at a post-transcriptional level by degrading *Nos2* mRNA (Fig. S4). The inhibition of these miRNAs blocked this degradation and stabilized the mRNA, sustaining translation and increasing the levels of NOS2 and NO production. On the other hand, competition for the binding site of miR-294-3p and miR-721 in the *Nos2* 3′UTR, stabilized the mRNA promoting protein translation and NO production. The interaction of miR-294-3p and/or miR-721 with the *Nos2* 3′UTR was directly shown by pmiRGLO constructs and luciferase assay. All these observations indicate that the regulation by miR-294-3p and miR-721 occurred at mRNA stability level. This effect stands in contrast to the action of miR-939 in human hepatocytes^51^, miR-26a in human T cell lymphoma^52^ and miR-146a in mouse renal carcinoma cells^53^ that were shown to inhibit translation.

This communication shows the role of miR-294 and miR-721 in *Leishmania* subversion of macrophage NO production in a parasite arginase/L-arginine metabolism-dependent manner leading to the evasion from macrophage inflammatory response and survival of *L*. (*L*.) *amazonensis*. This subversion also includes the use of L-arginine to supply polyamine pathway, favoring parasite replication (Fig. S4).

Altogether, our results indicate that the alteration of miRNA profile of infected macrophages, including the modulation of miR-294-3p and miR-721, by L-arginine metabolism*/Leishmania* arginase can be used as a target to improve the host response leading to parasite killing and infection control.

## Materials and Methods

### Ethics Statement

Experimental protocol for the animal experiments was approved by the Comissão de Ética no Uso de Animais (CEUA) from Instituto de Biociências of the Universidade de São Paulo (the approval number CEUA-IB: 169/2012). This study was carried out in strict accordance with the recommendations in the guide and policies for the care and use of laboratory animals of the São Paulo State (Lei Estadual 11.977, de 25/08/2005) and Brazil government (Lei Federal 11.794, de 08/10/2008).

### Parasite culture

*L*. (*L*.) *amazonensis* (MHOM/BR/1973/M2269) promastigotes were maintained in culture at 25 °C in M199 medium (Invitrogen, Grand Island, NY, USA) supplemented with 10% heat-inactivated fetal bovine serum (Invitrogen), 5 ppm hemine, 100 μM adenine, 100 U penicillin, 100 μg/mL streptomycin, 40 mM Hepes-NaOH and 12 mM NaHCO3, pH 6.85, for a week-long culture at a low passage (P1-5). *L*. (*L*.) *amazonensis La*-*arg*^*−*^ promastigotes were maintained in the same medium supplemented with 30 μg/mL hygromycin B and 30 μg/mL puromycin (Sigma, St. Louis, MO, USA), as well as 50 μM putrescine (Sigma)^35^. The arginase addback, *La*-*arg*^*−*^ + ARG were maintained in the same medium of *La*-*arg*^*−*^ supplemented with 20 μg/mL phleomycin^35^.

### *In vitro* macrophage infections

All experiments were performed using female from 6- to 8-week-old BALB/c mice from Biotério do Departamento de Fisiologia, Instituto de Biociências/USP. The animals were sacrificed in a CO_2_ chamber for obtained bone marrow-derived macrophages (BMDMs). The femurs were washed with 2 mL of PBS, and the cells were washed with PBS, collected at 1,500 × g for 10 min at 4 °C and resuspended in RPMI 1640 medium (LGC Biotecnologia, São Paulo, SP, Brazil) supplemented with penicillin (100 U/ml) (Invitrogen), streptomycin (100 μg/ml) (Invitrogen), 5% heat-inactivated FBS (Invitrogen) and 20% L9-29 supernatant. The cells were cultivated for 7–8 days at 34 °C in an atmosphere of 5% CO_2_. BMDMs were used after phenotypic analysis by flow cytometry (FACScalibur-Becton Dickinson, San Jose, CA, USA) demonstrated the presence of 95% F4/80- and CD11b-positive cells. The BMDMs were seeded into 8-well glass chamber slides (Lab-Teck Chamber Slide; Nunc, Naperville, IL, USA) (2 × 10^5^/well) for infectivity analysis or into 24-well plates (SPL, Lifescience, Pocheon, Korea) (1 × 10^6^/well) for miRNA and mRNA analysis. After 18 h of incubation at 34 °C in an atmosphere of 5% CO_2_, stationary phase promastigotes were added to each well (MOI 5). After 4 h at 34 °C in an atmosphere of 5% CO_2_, non-phagocytosed promastigotes were washed with fresh medium. The infection course was followed for 4, 12, 24 and 48 h with sample collection for RNA extraction or fixation of the slides for the determination of infectivity indexes. For infectivity analysis, the cells were fixed with acetone/methanol (1:1, v:v, Merck) for 20 min at −20 °C, washed in PBS and stained with DAPI (Invitrogen). The infectivity indexes (rate of infected macrophages multiplied by the average number of amastigotes per macrophage) were calculated by randomly counting at least 600 macrophages per slide. For RNA analysis, the cells were treated with trypsin (0.5% trypsin, 10 min, 34 °C), washed with PBS at 1,500 × g for 10 min at 4 °C, resuspended in Qiazol (Qiagen, Germantown, MD, USA) and stored at −20 °C.

### Reverse transcription and quantitative real-time PCR for miRNA

RNA was obtained using the miRneasy^®^ Mini kit (Qiagen, Hilden, Germany), following the manufacturer’s instructions. cDNA was produced from mature miRNA using the miScript II RT Kit (Qiagen, Hilden, Germany). Briefly, 250 ng of total RNA were added to 2 μL of 5x miScript HiSpec Buffer, 1 μL of 10x Nucleics Mix, and 1 μL of miScript Reverse Transcriptase Mix, and RNAse-free water was added to a final volume of 10 μL. The RNA was incubated for 60 min at 37 °C for the insertion of PoliA+ at the end of the miRNA sequence (downstream) and the annealing of a T-tail-tag for the elongation of the cDNA. The enzyme was inactivated at 95 °C for 5 min. The reaction was performed in a Thermocycler Mastercycler gradient (Eppendorf, Hamburg, Germany). The product was stored at −20 °C.

For miRNA quantification via real-time qPCR, the ten-fold diluted cDNA was analyzed using the Mouse Inflammatory Response & Autoimmunity miRNA PCR Array: MIMM-105Z (Qiagen, Germantown, MD, USA) and the miScript SYBR PCR Kit (Qiagen, Hilden, Germany). The reaction was performed with 2x QuantiTect SYBR Green PCR Master Mix, 10x miScript Universal Primer, and 105 μL of cDNA (3 samples diluted 10-fold), and RNAse-free water was added to a final volume of 2,625 μL (25 μL/well). For specific amplification of miR-294-3p, miR-721 and SNORD95A (used in normalization), real time reactions were performed with with 2x QuantiTect SYBR Green PCR Master Mix, 10x miScript Universal Primer, 10x Specific Primer and 5 μL of cDNA (3 samples diluted 10-fold), and RNAse-free water was added to a final volume of 25 μL (25 μL/well). The PCR reaction consisted of the activation of the HotStart DNA Polymerase for 15 s at 95 °C and 40 cycles of 15 s at 94 °C, followed by 30 s at 55 °C and 30 s at 70 °C. qPCR was performed in a Thermocycler ABI Prism 7300 (Applied Biosystems, Carlsbad, CA, USA), and the relative Ct was analyzed using the miScript miRNA PCR Array Data Analysis software (www.qiagen.com). Triplicate samples were analyzed for each condition. The average Ct was calculated to show the gene expression variation with good accuracy. The geometric average Ct of the miRNAs was normalized based on the average of snoRNAs (SNORD95A and SNORD96). The PCR and RT-PCR reaction efficiencies were determined, and a negative control containing all reaction components except the reverse transcriptase enzyme was included and subjected to real-time PCR to exclude the possibility of DNA contamination in the RNA samples. The Fold Regulation is the negative inverse of the fold-change (function = −1*(1/fold-change value)). The Fold Regulation levels greater than or equal to 2.0 were considered to indicate up-regulation, and levels less than or equal to −2.0 were considered to indicate down-regulation.

### Reverse transcription and quantitative real-time PCR for mRNA

RNA was obtained using the miRNeasy^®^ Mini kit (Qiagen), according to the manufacturer’s instructions. cDNA was produced from mRNAs using 200 U RevertAid™ Reverse Transcriptase (Fermentas Life Sciences, Burlington, Ontario, Canada) with 20 nmol random primer (Applied Biosystems, Carlsbad, CA, USA) and 1 μg of total RNA in a final volume of 13 μL. The RNA was denatured at 70 °C for 5 minutes and then cooled to 15 °C. A total of 4 μL of 5x Buffer and 2 μL of 10 mM dNTPs were then added. The reaction was heated to 37 °C for 5 minutes, and 1 μL (2 U) of reverse transcriptase (Revertaid Reverse Transcriptase, Fermentas Life Science, Burlington, Ontario, Canada) was then added to the reaction, followed by incubation at 42 °C for 60 minutes. The enzyme was inactivated at 75 °C for 15 minutes, and the reaction was stored at −20 °C. A negative control containing all reaction components except the reverse transcriptase enzyme was included and subjected to real-time PCR to exclude the possibility of DNA contamination in the RNA samples.

For mRNA quantification by real-time qPCR, 100-fold diluted cDNA was used as a template. The reaction was performed with 2x SYBR Green PCR Master Mix (Applied Biosystems), 0.4 μM of each corresponding primer pair, and 5 μL of cDNA (diluted 100-fold), and RNAse-free water was added to a final volume of 25 μL. The PCR reaction consisted of 40 cycles of 30 s at 94 °C followed by 30 s at 60 °C. qPCR was performed in an Exicycler™ 96 Real-Time Quantitative Thermal Block (Bioneer, Daejeon, Korea). Triplicate samples were analyzed for each condition. Target gene expression was quantified based on a standard curve prepared from a ten-fold serial dilution of a quantified and linearized plasmid containing the target DNA. The following primer pairs were utilized for mammalian mRNA analysis: *Nos2*: 5′-agagccacagtcctctttgc-3′ and 5′-gctcctcttccaaggtgctt-3′; *ArgI*: 5-agcactgaggaaagctggtc-3′ and 5′-cagaccgtgggttcttcaca-3′; *Cat*-*2b*: 5′-tatgttgtctcggcaggctc-3′ and 5′-gaaaagcaacccatcctccg-3′; *Cat1*: 5′-cgtaatcgccactgtgacct-3′ and 5′-ggctggtaccgtaagaccaa-3′; and *Gapd*: 5′-ggcaaattcaacggcacagt-3′ and 5′-ccttttggctccacccttca-3′. For *Leishmania* mRNA analysis, the following primer pairs were used: *arg*: 5′-atcctgttggggcttgatcg-3′ and 5′-acagcacgcagaccaatgta-3′; *aap3* copy 5.1: 5′-ccctgcctactcggacaatc-3′ and 5′-gagacagagcgacacggaag-3′; *aap3* copy 4.7: 5′-accattgtgggttagttatacatcc-3′and 5′-caagatcgctagcagtggag-3′; and *gapd*: 5′-caaggtcggtatcaacggc-3′ and 5′-tgcaccgtgtcgtacttcat-3′.

### Detection of NOS2 via in-cell western blotting

Infected BMDMs were fixed with methanol/acetone (1:1; 15 min at −20 °C) and permeabilized with 0.5% Tween 20 for 30 min at 4 °C, followed by blocking with 3% PBS/BSA for 1 hour at room temperature. Then, the samples were incubated with 1:200 dilutions of rabbit anti-NOS2 (sc651) or mouse anti-B-actin (s47778) antibodies (Santa Cruz, CA, USA) for 16 hours at 4 °C. The samples were then incubated with 1:5000 dilutions of IRDye^®^800CW-conjugated goat anti-rabbit IgG (827–08365) or IgG IRDye^®^680 LT-conjugated donkey anti-mouse (926–68022) antibodies (LI-COR, Lincoln, NE, USA) for 1 hour at 4 °C. The fluorescence of each well was measured at 700 nm and 800 nm using an Odyssey CLx system (LI-COR, Bad Homburg, Germany). To determine the relative NOS2 fluorescence intensity, the values were normalized by B-actin fluorescence using the Image software (LI-COR).

### Quantification of NO production

BMDMs were seeded into 24-well plates (SPL) (1 × 10^6^/well) for 18 h of incubation at 34 °C in an atmosphere of 5% CO_2_. Then, the macrophages were infected as described above for 4 and 24 h. The samples were detached with 0.5 mM EDTA in PBS for 10 min at 37 °C, scraped and washed with PBS. The samples were incubated with 5 μM DAF-FM (for NO quantification) and 500 nM Vybrant Orange Dye (for nuclei acid staining) (Molecular Probes, Life Technologies, Darmstadt, Germany) in PBS for 30 min at 34 °C in an atmosphere of 5% CO_2_. Fluorescence acquisition was performed using a FlowSight Amnis (Merck-Millipore, Darmstadt, Germany) and analyzed using the Ideas^®^ Software (Amnis Corporation, Seattle, WA, USA).

### Transfection of miRNA inhibitors

BMDMs were seeded into 24-well plates (SPL) (5 × 10^5^/well) for miRNA, mRNA, protein and NO analysis or into 8-well glass chamber slides (Lab-Teck Chamber Slide; Nunc, Naperville, IL, USA) (2 × 10^5^/well) for infectivity analysis. After 18 h of incubation at 34 °C in an atmosphere of 5% CO_2_, the cells were incubated for 24 h with 30 and 100 nM of the miR-294-3p inhibitor, the miR-721 inhibitor or the negative control (Ambion, Carlsbad, CA, USA), which was previously incubated for 20 min at room temperature with 3 μL of the FugeneHD transfection reagent (Roche, Madison, WI, USA) in 250 μL of serum-free RPMI 1640 medium (LGC Biotecnologia, São Paulo, SP, Brazil). After transfection, the cells were infected as described above.

### Transfection of miScript target protectors

The miScript target protector (Qiagen) designed for the 3′UTR of Nos2 contained the predicted sequence for the miR-294/Nos2 (5′-aacucaaccuccugacugaagcacuuugggugaccaccag-3′) or miR-721/Nos2 (5′-ugccgccgcucuaauacuuagcugcacuauguacagauau-3) interaction. BMDMs were seeded into 8-well glass chamber slides (Lab-Teck Chamber Slide; Nunc, Naperville, IL, USA) (2 × 10^5^/well) and incubated for 18 h at 34 °C in an atmosphere of 5% CO_2_. The cells were then incubated for 24 h with 0.1, 0.5 or 1 μM solution of the miScript Target Protector (Qiagen, Maryland, USA), which was previously incubated for 20 min at room temperature with 250 μL of serum-free RPMI 1640 medium (LGC Biotecnologia, São Paulo, SP, Brazil) containing 3 μL of the FugeneHD transfection reagent (Roche). After transfection, the cells were infected as described above.

### Transfection of pmiRGLO constructs and luciferase activity assay

The pmiRGLO constructs were designed for pmirGLO Dual-Luciferase miRNA Target Expression Vector (Promega) and the 3′UTR of *Nos2* contained the predicted sequence for the miR-294 or miR-721 interaction (5′- aacctcctgactgaagcactttgggtgaccaccaggaggcaccatgccgccgctctaatacttagctgcact-3′) (Fig. 7A). BMDMs were seeded into 24-well plates (SPL) (5 × 10^5^/well) and incubated for 18 h at 34 °C in an atmosphere of 5% CO_2_. The cells were then incubated for 24 h with 5 μg solution of the pmiRGLO constructs and 100 nM of miRNAs mimics miR-294 or miR-721 (Qiagen), which was previously incubated for 20 min at room temperature with 250 μL of serum-free RPMI 1640 medium (LGC Biotecnologia, São Paulo, SP, Brazil) containing 5 μL of the FugeneHD transfection reagent (Roche). After 24 h of transfection, the cells were lysated with 100 μL RIPA buffer (Sigma) at 4 °C for 15 min. The protein extract was clarified by centrifugation (8000 g, 10 min, 4 °C). For luciferase activity assay, 20 μL of each extract were incubated with 20 μL of Dual-Glo^®^ Luciferase reagent for 5 min, at room temperature and firefly luciferase luminescence measured at luminometer. Renilla luciferase luminescence was analysed by incubation with 100 μL of Dual-Glo^®^ Stop-Glo^®^ Substrate for 10 min, at room temperature and luminescence measure. The reagents were from Dual-Glo^®^ Luciferase Assay kit (Promega) and luminometer used was the GloMax^®^ 96 Microplate Luminometer (Promega). The values of firefly luciferase were normalized by renilla luciferase and compared to pmiRGLO-empty vector.

### Statistical analysis

Statistical significance was determined based on Student’s t test or an ANOVA using the GraphPad Prism Software (GraphPad Software, Inc., La Jolla, CA, USA). The obtained p-values are indicated throughout the Results section.

## Additional Information

**How to cite this article**: Muxel, S. M. *et al. Leishmania (Leishmania*) *amazonensis* induces macrophage miR-294 and miR-721 expression and modulates infection by targeting NOS2 and L-arginine metabolism. *Sci. Rep.* **7**, 44141; doi: 10.1038/srep44141 (2017).

**Publisher's note:** Springer Nature remains neutral with regard to jurisdictional claims in published maps and institutional affiliations.

## Supplementary Material

Supplementary Information
