# Supplementary material for: Leishmania (Leishmania) amazonensis induces macrophage miR-294 and miR-721 expression and modulates infection by targeting NOS2 and L-arginine metabolism
Source: Sci Rep. 2017 Mar 9;7:44141. doi: 10.1038/srep44141 (PMC5343489; doi:10.1038/srep44141)
Supplement: Supplementary Information [file srep44141-s1.pdf]

Supporting information

Leishmania (Leishmania) amazonensis induces macrophage miR-294 and miR-721 expression and modulates infection by targeting NOS2 and L-arginine metabolism

Sandra Marcia Muxel[1], Maria Fernanda Laranjeira-Silva, Ricardo Andrade Zampieri, Lucile Maria Floeter-Winter[1].

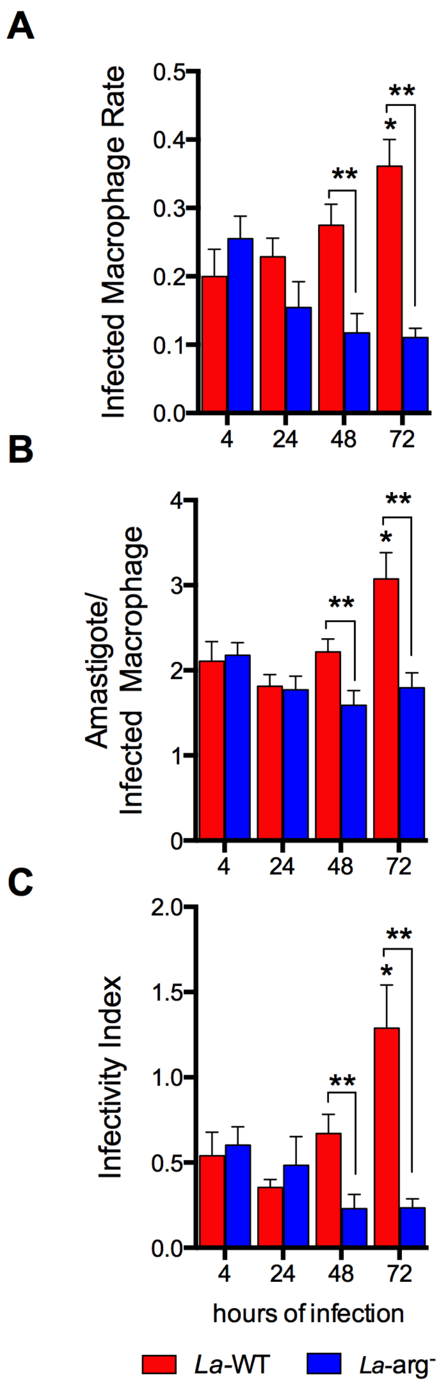

Supplementary Figure S1: Comparative analysis of BMDMs infected with *La*-WT and *La-arg*⁻ *L. (L.) amazonensis*. BMDMs (2 x 10^5) were plated into chamber slides overnight and infected with *La*-WT or *La-arg*⁻ *L. (L.) amazonensis* (MOI 5:1). After 4 h, the cultures were washed and maintained for 12, 24, 48 and 72 h. The plates were fixed and stained with Giemsa, and the index of infection was determined via microscopic counting of infected macrophages and amastigotes per macrophage. The data represent the mean ± SEM of 3 independent experiments (n = 600 macrophages). *, p<0.05, compared to 4 hours of infection. **, p<0.05, WT compared to *La-arg*⁻.

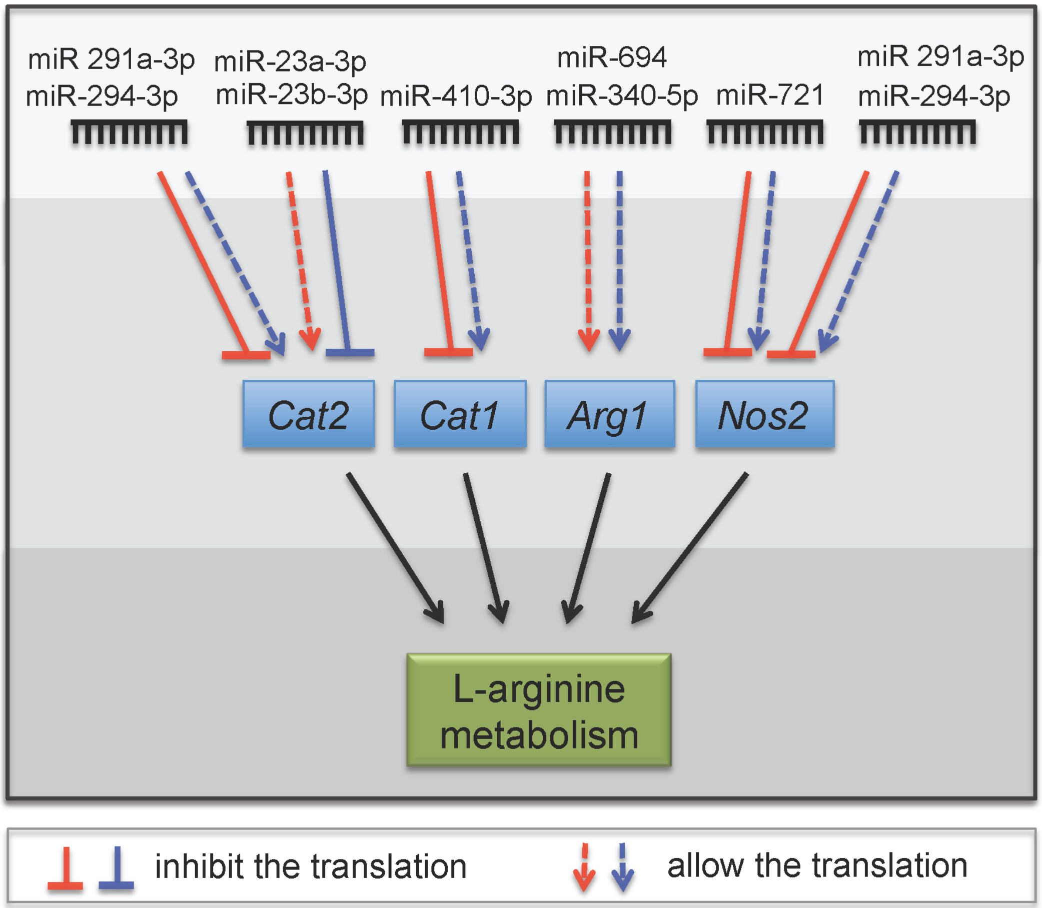

Supplementary Figure S2: Schematic representation of in silico analysis results from the search for predicted mRNA targets for the miRNAs modulated during *L. (L.) amazonensis* infection. The predicted mRNA targets for the miRNAs modulated during *La*-WT (red lines) or *La-arg*$^-$ (blue lines) *L. (L.) amazonensis* infection suggested the modulation of mRNAs that code for enzymes involved in arginine metabolism, such as miR-291 and miR-294, which target *Cat2b* and/or *Nos2*, miR-410, which targets *Cat1,* and miR-721, which targets *Nos2*. Predictions were made using microRNA.org, TargetScan and the miRWalk database.

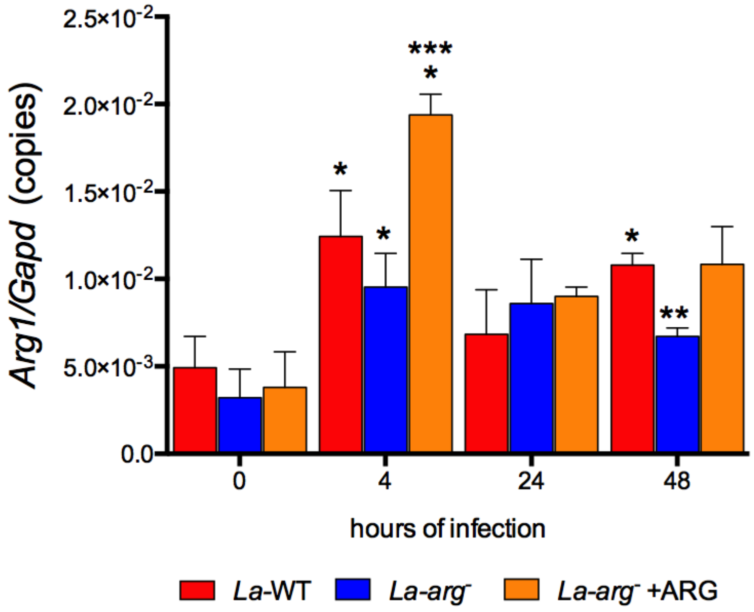

Supplementary Figure S3: Arg1 mRNA in BMDMs infected by *La*-WT, *La-arg*⁻ and *La-arg*⁻+ARG *L. (L.) amazonensis.* BMDMs (1 x 10^6) were plated overnight and co-cultivated with *La*-WT (red) or *La-arg*⁻ (blue) or *La-arg*⁻+ARG (orange) *L. (L.) amazonensis* (MOI 5:1). After 4, 24 and 48 h of infection, the Arg1 mRNA from *L. (L.) amazonensis* was quantified via RT-qPCR. Each bar represents the mean ± SEM of the values obtained in 3 independent experiments. *, $p<0.05$, compared to uninfected macrophages (0 hours of infection). ***, $p<0.05$, WT compared to *La-arg*⁻ .**, $p<0.05$, *La-arg*⁻+ARG compared to *La-arg*⁻.

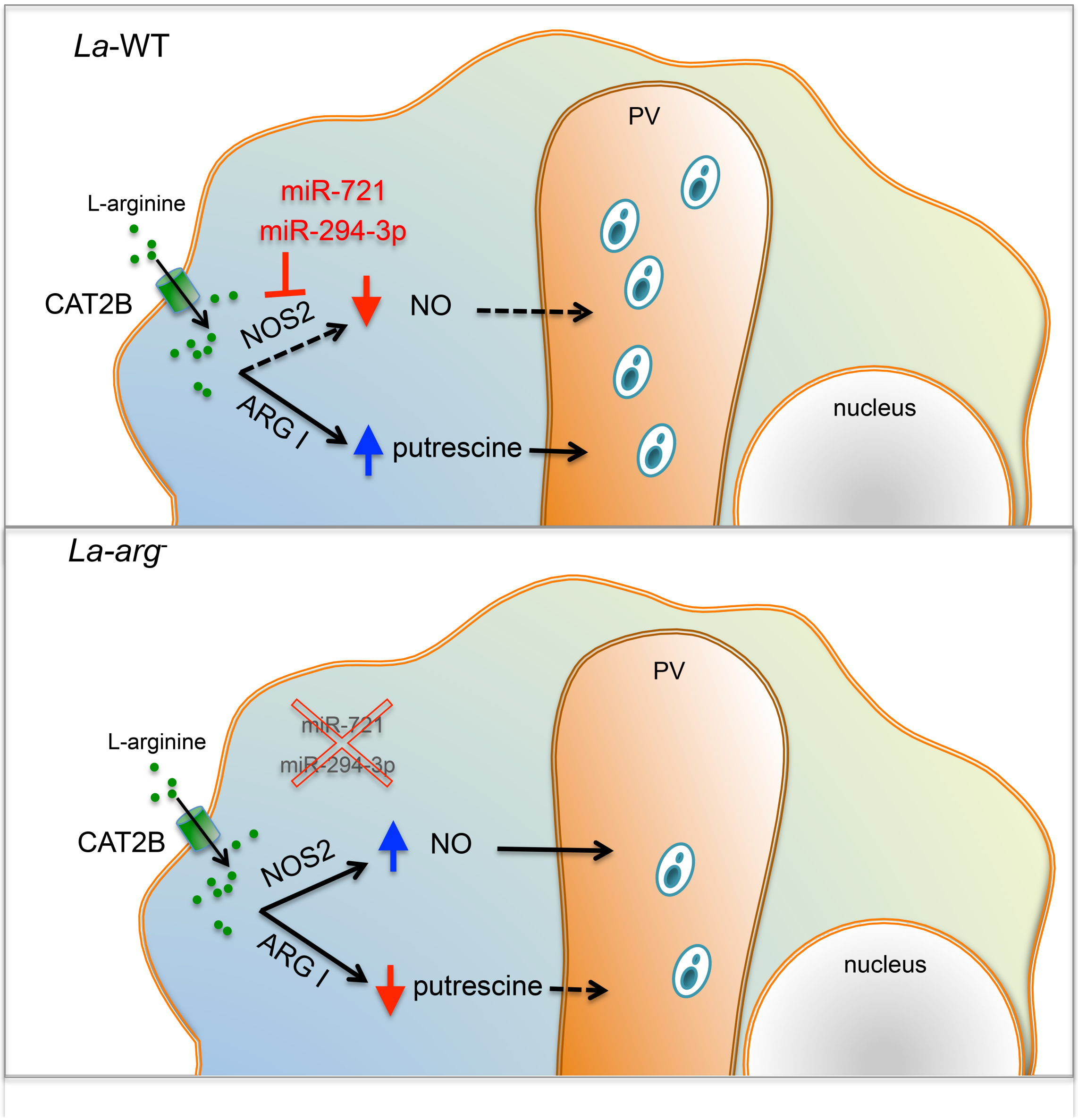

Supplementary Figure S4: Schematic representation of the effects of miR-294 and miR-721/*Nos2* mRNA targets in the L-arginine/NO pathway during *L. (L.) amazonensis* infection. Infection of macrophages increases the activity of polyamine pathways that promote *Leishmania* survival by increasing the expression of the host L-arginine transporters CAT2B and CAT1, which increase L-arginine uptake and ARG1 activity. At the same time, the negative regulation of the NOS2- and NO-producing pathway, which was induced by the miR-294 and miR-721 expression that degraded *Nos2* mRNA, consequently inhibited NO production and parasite killing. PV: parasitophorous.

S1 Table: Macrophage miRNA regulation during WT *L. (L.) amazonensis* (*La*-WT) infection. Fold (Fold regulation) represents the fold change results at a biological magnitude. Values > 1 indicate positive regulation and values < 1 indicate negative regulation.

	***La*-WT - 4 h**		***La*-WT - 12 h**		***La*-WT - 24 h**		***La*-WT - 48 h**	
**miRNA**	**Fold**	**p-value**	**Fold**	**p-value**	**Fold**	**p-value**	**Fold**	**p-value**
let-7a-5p	1.17	0.8820	1.40	0.7732	-1.01	0.6689	-1.16	0.4418
let-7b-5p	1.04	0.9237	-1.05	0.9237	-1.27	0.6551	1.14	0.9976
let-7c-5p	-1.08	0.8911	-1.28	0.7589	-1.05	0.8007	1.09	0.9803
let-7d-5p	1.02	0.8191	-1.14	0.9079	1.05	0.8512	1.02	0.7536
let-7e-5p	1.07	0.8194	-1.19	0.7045	1.41	0.4324	1.37	0.4697
let-7f-5p	1.34	0.6283	-1.07	0.9370	1.71	0.4744	1.41	0.4973
let-7g-5p	1.34	0.4750	-1.21	0.6552	1.36	0.4630	1.64	0.3109
let-7i-5p	-1.10	0.9861	-1.55	0.0552	1.41	0.3460	1.78	0.1056
miR-106a-5p	-1.01	0.9079	-1.81	0.1971	1.19	0.6802	1.05	0.9804
miR-106b-5p	1.16	0.8894	-1.49	0.5804	1.68	0.4968	1.58	0.5048
miR-1192	-1.19	0.9557	1.67	0.3468	1.09	0.9705	-1.04	0.9532
miR-126a-5p	1.05	0.9178	1.07	0.7431	-1.09	0.5843	-2.34	0.0961
miR-128-3p	1.03	0.9162	1.18	0.6576	1.01	0.9521	-1.04	0.7376
miR-130a-3p	-1.14	0.8207	1.08	0.7742	1.25	0.8675	1.09	0.8775
miR-130b-3p	-1.06	0.8585	-1.00	0.7952	-1.11	0.7662	-1.19	0.4197
miR-135a-5p	1.87	0.4177	1.49	0.4584	-1.00	0.8662	-1.09	0.5180
miR-140-5p	1.23	0.4755	1.24	0.5097	1.93	0.2335	2.22	0.1081
miR-144-3p	-1.78	0.5229	-1.18	0.6482	-2.04	0.0045	-1.13	0.1340
miR-155-5p	-1.03	0.9675	1.14	0.9405	-1.33	0.5555	-1.63	0.3313
miR-15a-5p	-1.02	0.8139	-1.43	0.7338	2.35	0.4923	3.11	0.4453
miR-15b-5p	1.11	0.6061	-1.22	0.3472	1.07	0.6796	-1.46	0.3598
miR-16-5p	1.11	0.7407	-1.25	0.4948	1.66	0.3019	1.59	0.0647
miR-17-5p	1.03	0.9526	-1.38	0.5086	1.20	0.6253	1.14	0.7624
miR-181a-5p	1.08	0.8842	-1.13	0.9869	1.33	0.9546	1.32	0.9527
miR-181b-5p	-1.09	0.9555	1.20	0.7281	-1.45	0.3278	-1.64	0.1442
miR-181c-5p	1.41	0.7011	-1.04	0.7176	1.50	0.8053	1.35	0.9596
miR-181d-5p	-1.24	0.6343	1.00	0.8268	-1.47	0.3236	-2.11	0.0874
miR-182-5p	1.83	0.4975	2.89	0.2371	1.52	0.7322	-1.04	0.5491
miR-186-5p	1.27	0.6001	1.16	0.8061	1.93	0.2396	2.02	0.1234
miR-195a-5p	1.20	0.6483	-1.11	0.7966	2.06	0.0850	1.92	0.2148
miR-19a-3p	1.21	0.9892	1.13	0.9659	1.54	0.6247	1.37	0.8799
miR-19b-3p	1.07	0.8613	1.03	0.8997	1.76	0.5421	1.31	0.9979
miR-200c-3p	1.14	0.7544	1.44	0.5834	1.31	0.7115	-1.44	0.2596
miR-20a-5p	1.13	0.7788	-1.24	0.5830	1.27	0.5493	1.09	0.9424
miR-20b-5p	1.11	0.6474	-1.45	0.2390	1.55	0.3332	1.45	0.2128
miR-221-3p	-1.27	0.3823	-1.93	0.2220	-1.70	0.0239	-1.60	0.5085
miR-222-3p	1.20	0.4276	1.30	0.4888	1.69	0.1477	1.33	0.2591
miR-23a-3p	1.24	0.5976	1.36	0.4801	1.36	0.5421	1.12	0.4810
miR-23b-3p	1.16	0.6596	1.17	0.5772	1.17	0.7326	1.12	0.5756
miR-26a-5p	1.39	0.4762	1.40	0.4634	1.24	0.5322	1.34	0.4297
miR-26b-5p	-1.06	0.9860	-1.24	0.9411	-1.12	0.7641	1.15	0.6169
miR-27a-3p	1.12	0.6674	1.25	0.5462	2.15	0.1395	2.25	0.1876
miR-27b-3p	1.15	0.6621	1.00	0.9103	1.49	0.3823	1.59	0.3284
miR-291a-3p	1.29	0.6757	2.27	0.3761	1.67	0.4112	2.54	0.1124

miR-294-3p	2.99	0.0755	3.11	0.0336	2.91	0.0075	2.54	0.0013
miR-295-3p	-1.31	0.8944	1.06	0.8158	-2.06	0.0619	-1.64	0.3176
miR-29a-3p	1.08	0.9691	1.38	0.5798	1.92	0.3752	2.40	0.0234
miR-29b-3p	-1.17	0.6712	1.21	0.8733	1.72	0.6550	3.24	0.5682
miR-29c-3p	1.32	0.4925	1.14	0.5936	1.75	0.2697	2.88	0.0005
miR-301a-3p	1.10	0.9119	1.58	0.8811	1.36	0.7978	-1.23	0.8012
miR-301b-3p	-1.00	0.8133	1.29	0.8819	1.06	0.9834	-1.64	0.5345
miR-302b-3p	-1.19	0.9557	1.91	0.1780	-1.05	0.9822	-1.04	0.9532
miR-302d-3p	1.13	0.5771	1.14	0.6670	1.46	0.3999	1.12	0.6187
miR-30a-5p	1.04	0.7539	1.16	0.5715	1.55	0.1021	1.77	0.1653
miR-30b-5p	1.00	0.8925	1.00	0.7983	1.75	0.1579	1.81	0.2600
miR-30c-5p	-1.04	0.9466	1.01	0.7700	1.38	0.1849	1.04	0.6168
miR-30d-5p	-1.15	0.3888	1.12	0.6162	1.33	0.2501	1.62	0.1528
miR-30e-5p	1.22	0.4936	1.12	0.7221	1.83	0.1751	1.75	0.1775
miR-322-5p	-1.03	0.8470	1.73	0.3329	1.37	0.3715	1.30	0.4477
miR-325-3p	-1.19	0.9557	1.67	0.3468	-1.34	0.4404	-1.04	0.9532
miR-338-5p	1.11	0.7981	1.53	0.4134	1.09	0.9545	1.16	0.7965
miR-340-5p	1.05	0.6783	1.08	0.5701	1.20	0.3979	1.67	0.0772
miR-350-3p	1.04	0.7134	1.06	0.6451	1.23	0.3666	1.16	0.5043
miR-369-3p	1.21	0.6266	1.31	0.9744	1.15	0.6222	1.97	0.3333
miR-384-5p	-1.19	0.9557	1.67	0.3468	-1.34	0.4404	-1.04	0.9532
miR-410-3p	1.66	0.2505	2.18	0.1515	1.89	0.0505	2.10	0.0091
miR-429-3p	-1.17	0.8147	1.20	0.5397	1.22	0.4967	1.25	0.4512
miR-466d-3p	1.21	0.7268	1.76	0.5170	1.38	0.9989	1.17	0.8057
miR-466k	1.19	0.6417	1.34	0.4841	1.20	0.6037	2.28	0.2241
miR-495-3p	-1.00	0.5835	-1.28	0.4540	1.08	0.9786	1.63	0.6259
miR-497a-5p	1.54	0.2460	1.19	0.5227	1.33	0.5579	1.87	0.0480
miR-568	1.10	0.7735	1.96	0.2428	-1.11	0.5850	1.37	0.5412
miR-590-3p	1.00	0.8191	2.41	0.2668	-1.13	0.5664	-1.04	0.9532
miR-669h-3p	1.64	0.5478	1.90	0.4714	1.48	0.7262	2.40	0.2020
miR-669k-3p	1.25	0.6731	1.98	0.2806	1.27	0.5789	1.48	0.4194
miR-694	-1.69	0.2586	-2.36	0.0345	-1.90	0.1216	-2.20	0.0696
miR-712-5p	-1.32	0.9230	-1.51	0.9969	-1.22	0.4622	1.21	0.9068
miR-721	4.16	0.2736	5.06	0.2738	3.96	0.1231	4.51	0.0396
miR-743a-3p	-1.14	0.8515	1.36	0.3648	-1.26	0.4002	1.16	0.5971
miR-743b-3p	-1.19	0.9557	1.67	0.3468	-1.34	0.4404	-1.04	0.9532
miR-876-3p	-1.19	0.9557	1.67	0.3468	-1.34	0.4404	-1.04	0.9532
miR-9-5p	1.51	0.6132	2.38	0.3937	1.57	0.6698	1.01	0.6005
miR-93-5p	1.04	0.9637	1.03	0.9249	1.11	0.8823	1.12	0.9534
miR-98-5p	-1.28	0.9754	1.02	0.9741	-1.34	0.5357	-1.47	0.4957

S2 Table: Macrophage miRNA regulation during *La-arg*$^-$ *L. (L.) amazonensis* infection. Fold (Fold regulation) represents the fold change results at a biological magnitude. Values > 1 indicate positive regulation. and values < 1 indicate negative regulation.

	***La-arg*$^-$ - 4h**		***La-arg*$^-$ - 12h**		***La-arg*$^-$ - 24h**		***La-arg*$^-$ - 48h**	
**Mature ID**	**Fold**	**p-value**	**Fold**	**p-value**	**Fold**	**p-value**	**Fold**	**p-value**
let-7a-5p	-1.27	0.4881	-1.13	0.4627	-1.37	0.4559	-1.49	0.4359
let-7b-5p	-1.96	0.2567	-2.29	0.1065	-1.49	0.4624	-1.74	0.3115
let-7c-5p	-1.80	0.2802	-2.30	0.0755	-1.60	0.3570	-1.48	0.4258
let-7d-5p	-1.31	0.5498	-1.54	0.0604	-1.33	0.4466	-1.46	0.2943
let-7e-5p	-1.10	0.9761	-1.38	0.2765	-1.16	0.8751	-1.15	0.8578
let-7f-5p	1.47	0.6533	-1.09	0.4723	1.12	0.9061	1.41	0.6544
let-7g-5p	1.55	0.3546	-1.26	0.3493	1.10	0.7145	1.06	0.7168
let-7i-5p	-1.11	0.9611	-1.75	0.0027	-1.41	0.8571	-1.29	0.9989
miR-106a-5p	1.12	0.9423	-1.73	0.1395	-1.47	0.5038	-1.91	0.3428
miR-106b-5p	-1.03	0.6969	-1.52	0.3139	-1.29	0.5723	-1.62	0.3948
miR-1192	1.42	0.5706	-1.41	0.2721	-1.07	0.7448	1.48	0.4970
miR-126a-5p	2.02	0.3208	1.81	0.3038	-1.33	0.6904	-4.52	0.1012
miR-128-3p	1.29	0.4639	-1.35	0.2808	-1.31	0.6001	-1.21	0.8095
miR-130a-3p	-1.36	0.4806	-1.77	0.2866	-1.62	0.4248	-1.17	0.5606
miR-130b-3p	-1.16	0.5467	-3.06	0.1005	-1.63	0.2675	-1.76	0.2139
miR-135a-5p	-1.03	0.8509	-1.46	0.2316	-2.59	0.2358	-1.77	0.4537
miR-140-5p	1.07	0.9009	-2.50	0.1236	-1.05	0.8691	-1.05	0.7823
miR-144-3p	-1.39	0.0407	-2.78	0.0003	-2.09	0.0202	-1.95	0.0344
miR-155-5p	-1.35	0.4753	-1.44	0.3660	-2.19	0.3587	-2.54	0.3410
miR-15a-5p	-1.89	0.3953	-3.45	0.2340	-1.41	0.6710	-1.77	0.4412
miR-15b-5p	1.23	0.3280	-1.22	0.0947	-1.28	0.3271	-1.44	0.1113
miR-16-5p	1.21	0.5283	-1.12	0.6741	1.14	0.7267	1.03	0.9660
miR-17-5p	1.20	0.6875	-1.83	0.1062	-1.56	0.3718	-1.82	0.2786
miR-181a-5p	-1.18	0.5619	-1.31	0.3991	-1.30	0.4962	-1.29	0.5057
miR-181b-5p	-1.44	0.3646	-1.54	0.1774	-2.17	0.1432	-2.19	0.1639
miR-181c-5p	1.03	0.7082	-1.26	0.4120	-1.03	0.6919	-1.21	0.5190
miR-181d-5p	-1.52	0.3074	-1.54	0.1662	-2.02	0.1628	-2.06	0.1769
miR-182-5p	1.40	0.4569	2.81	0.1868	1.61	0.7920	-1.39	0.6114
miR-186-5p	1.17	0.8141	-1.86	0.1151	1.11	0.8023	-1.02	0.9688
miR-195a-5p	1.44	0.3809	-1.12	0.6262	1.36	0.5238	1.35	0.5108
miR-19a-3p	-1.95	0.3733	-3.49	0.2123	-1.72	0.4576	-2.82	0.3141
miR-19b-3p	-2.11	0.3608	-4.45	0.1948	-2.01	0.4303	-2.97	0.3120
miR-200c-3p	1.46	0.5651	1.73	0.2046	1.07	0.9904	1.06	0.9177
miR-20a-5p	-1.09	0.6507	-1.62	0.1542	-1.43	0.5827	-1.82	0.2892
miR-20b-5p	-1.13	0.7408	-2.85	0.0598	-1.58	0.6387	-2.16	0.3621
miR-221-3p	-1.31	0.1347	-1.43	0.0379	-2.57	0.0130	-2.44	0.0355
miR-222-3p	-1.32	0.1957	-1.43	0.0940	-1.04	0.7316	-1.02	0.9696
miR-23a-3p	2.20	0.0891	1.14	0.9111	1.40	0.4986	1.30	0.5772
miR-23b-3p	1.38	0.4776	1.08	0.9985	1.07	0.9732	1.38	0.4723
miR-26a-5p	2.06	0.0280	1.13	0.7312	1.18	0.6467	1.56	0.2778
miR-26b-5p	1.18	0.8409	-1.81	0.1344	-1.24	0.9406	1.10	0.8707
miR-27a-3p	1.20	0.5850	-1.81	0.0603	1.15	0.5766	1.22	0.5519

miR-27b-3p	1.33	0.4735	-1.56	0.1930	-1.01	0.7725	-1.03	0.7317
miR-291a-3p	1.14	0.7421	1.29	0.8269	-1.20	0.5789	1.06	0.9486
miR-294-3p	2.00	0.1646	2.89	0.0265	2.33	0.1660	1.14	0.3988
miR-295-3p	-1.12	0.5291	-2.24	0.0168	-1.68	0.1539	-1.57	0.2078
miR-29a-3p	1.11	0.9316	-2.55	0.1141	-1.05	0.9576	-1.16	0.8368
miR-29b-3p	-2.08	0.3969	-4.98	0.2476	-2.13	0.4364	-1.94	0.4145
miR-29c-3p	1.04	0.9550	-3.19	0.0234	-1.26	0.9880	-1.15	0.9952
miR-301a-3p	-1.57	0.4899	-2.06	0.2956	-1.70	0.4172	-2.92	0.3293
miR-301b-3p	-1.72	0.4611	-1.95	0.3297	-2.01	0.3761	-2.61	0.3386
miR-302b-3p	1.42	0.5706	5.39	0.2108	-1.07	0.7448	1.59	0.4445
miR-302d-3p	-2.87	0.0494	-1.11	0.8629	-1.40	0.3678	-1.34	0.4052
miR-30a-5p	1.26	0.2468	-1.52	0.0536	1.08	0.6795	1.25	0.3998
miR-30b-5p	1.23	0.5094	-1.89	0.0382	1.19	0.5817	1.19	0.5732
miR-30c-5p	1.37	0.2843	-1.26	0.2612	1.11	0.7080	1.21	0.5440
miR-30d-5p	1.07	0.7146	-1.44	0.0409	-1.01	0.9060	1.03	0.7728
miR-30e-5p	1.14	0.6872	-1.20	0.3670	1.10	0.7707	1.15	0.6685
miR-322-5p	1.11	0.7248	-2.54	0.0299	1.20	0.5802	-1.30	0.8874
miR-325-3p	1.42	0.5706	-1.41	0.2721	1.26	0.8202	1.00	0.8471
miR-338-5p	-1.04	0.9051	1.09	0.9650	-1.09	0.6855	1.08	0.9882
miR-340-5p	-1.60	0.5224	-4.90	0.0111	-2.40	0.7433	-2.51	0.7208
miR-350-3p	1.18	0.4852	-1.60	0.0497	1.01	0.8766	1.07	0.6657
miR-369-3p	1.69	0.9093	-1.13	0.3998	1.61	0.6058	1.94	0.5248
miR-384-5p	1.42	0.5706	-1.41	0.2721	-1.07	0.7448	1.00	0.8471
miR-410-3p	1.11	0.7553	1.75	0.1538	-1.09	0.7448	-1.58	0.2186
miR-429-3p	1.15	0.6360	-1.36	0.1467	-1.06	0.9943	1.20	0.5614
miR-466d-3p	1.19	0.8139	-1.45	0.3863	1.02	0.6766	1.06	0.7236
miR-466k	-2.38	0.1621	-4.70	0.0106	-1.48	0.2968	-1.89	0.2376
miR-495-3p	-1.48	0.4217	-2.22	0.2426	-1.50	0.4315	-1.85	0.3383
miR-497a-5p	-1.10	0.8061	-2.21	0.1050	-1.96	0.7867	-2.21	0.1904
miR-568	1.39	0.5786	-1.19	0.5424	1.27	0.7655	-1.02	0.8287
miR-590-3p	1.42	0.5706	-1.41	0.2721	-1.07	0.7448	1.00	0.8471
miR-669h-3p	-1.16	0.5954	-1.36	0.3733	-1.45	0.4502	-1.91	0.3190
miR-669k-3p	2.83	0.0276	2.14	0.0837	1.61	0.3316	1.95	0.1005
miR-694	-3.19	0.0334	-3.90	0.0005	-2.81	0.1276	-3.29	0.0164
miR-712-5p	-2.61	0.2233	-1.81	0.2229	-1.16	0.5301	-1.04	0.5613
miR-721	2.44	0.1167	1.38	0.5460	3.06	0.3321	1.03	0.8722
miR-743a-3p	1.11	0.7925	-1.80	0.0641	1.06	0.9217	-1.23	0.6255
miR-743b-3p	1.42	0.5706	-1.41	0.2721	-1.07	0.7448	1.00	0.8471
miR-876-3p	1.42	0.5706	-1.41	0.2721	-1.07	0.7448	1.00	0.8471
miR-9-5p	2.64	0.0735	1.59	0.5085	1.48	0.7924	1.55	0.6411
miR-93-5p	1.22	0.7720	-1.10	0.5455	-1.20	0.5168	-1.21	0.5308
miR-98-5p	1.29	0.9597	-1.43	0.3352	-1.70	0.3962	-1.53	0.4946

S3 Table: In silico analysis of predicted miR-294 mRNA targets. Prediction analysis of miR-294 binding sequences in the 3' UTR of mRNA targets using the miRWalk software.

Gene Name	RefSeqID	Seed Length	Start	Sequence	End	Region	Pvalue
Wnt9b	NM_011719	14	1607	AAAGUGCUUCCCUU	1620	3 UTR	0.0000
Paf1	NM_019458	13	1868	AAAGUGCUUCCCU	1880	3 UTR	0.0000
Gtf3a	NM_025652	12	626	AAAGUGCUUCCC	637	3 UTR	0.0001
Zfp367	NM_175494	11	1536	AAAGUGCUUCC	1546	3 UTR	0.0005
Il25	NM_080729	11	681	AAAGUGCUUCC	691	3 UTR	0.0001
1200015N20Rik	NM_024244	11	2000	AAAGUGCUUCC	2010	3 UTR	0.0003
Il6st	NM_010560	11	3840	AAAGUGCUUCC	3850	3 UTR	0.0006
6030436E02Rik	NM_194340	11	2262	AAAGUGCUUCC	2272	3 UTR	0.0010
Rdh1	NM_080436	11	2718	AAAGUGCUUCC	2728	3 UTR	0.0007
Iws1	NM_173441	11	8720	AAAGUGCUUCC	8730	3 UTR	0.0017
Tyw1	NM_178897	11	2539	AAAGUGCUUCC	2549	3 UTR	0.0008
Tbxas1	NM_011539	11	1792	AAAGUGCUUCC	1802	3 UTR	0.0001
Mkrn1	NM_018810	10	2910	AAAGUGCUUC	2919	3 UTR	0.0013
Zfp560	NM_001004190	10	3592	AAAGUGCUUC	3601	3 UTR	0.0021
Serpine1	NM_008871	10	2104	AAAGUGCUUC	2113	3 UTR	0.0016
Reps2	NM_178256	10	5006	AAAGUGCUUC	5015	3 UTR	0.0053
Zfp280b	NM_177475	10	3136	AAAGUGCUUC	3145	3 UTR	0.0029
Irak2	NM_172161	10	2790	AAAGUGCUUC	2799	3 UTR	0.0012
Wrb	NM_207301	10	1468	AAAGUGCUUC	1477	3 UTR	0.0018
Grhl2	NM_026496	10	3497	AAAGUGCUUC	3506	3 UTR	0.0026
Ythdf3	NM_172677	10	2257	AAAGUGCUUC	2266	3 UTR	0.0028
Nhlrc1	NM_175340	10	2179	AAAGUGCUUC	2188	3 UTR	0.0010
Zc3h14	NM_029334	10	2714	AAAGUGCUUC	2723	3 UTR	0.0011
Sar1b	NM_025535	10	985	AAAGUGCUUC	994	3 UTR	0.0005
Stox2	NM_175162	10	6276	AAAGUGCUUC	6285	3 UTR	0.0062
Lamp2	NM_001017959	10	1472	AAAGUGCUUC	1481	3 UTR	0.0004
Aox3l1	NM_001008419	10	4366	AAAGUGCUUC	4375	3 UTR	0.0006
Arhgef7	NM_017402	10	4265	AAAGUGCUUC	4274	3 UTR	0.0020
Tshz3	NM_172298	10	3770	AAAGUGCUUC	3779	3 UTR	0.0015
4932425I24Rik	NM_001081025	10	2575	AAAGUGCUUC	2584	3 UTR	0.0003
Hn1	NM_008258	10	670	AAAGUGCUUC	679	3 UTR	0.0008
Ldoc1	NM_001018087	10	951	AAAGUGCUUC	960	3 UTR	0.0008
Cxcr6	NM_030712	10	1348	AAAGUGCUUC	1357	3 UTR	0.0007
Derl2	NM_033562	10	869	AAAGUGCUUC	878	3 UTR	0.0027
Pacs2	NM_001081170	10	4750	AAAGUGCUUC	4759	3 UTR	0.0026
Rgma	NM_177740	10	3415	AAAGUGCUUC	3424	3 UTR	0.0018
Rabgap1	NM_146121	10	3866	AAAGUGCUUC	3875	3 UTR	0.0015
Ano6	NM_175344	10	2898	AAAGUGCUUC	2907	3 UTR	0.0020
Ghsr	NM_177330	10	2975	AAAGUGCUUC	2984	3 UTR	0.0029
Tsr1	NM_177325	10	3355	AAAGUGCUUC	3364	3 UTR	0.0009
Arv1	NM_026855	10	1106	AAAGUGCUUC	1115	3 UTR	0.0003
Ica1l	NM_027407	10	2936	AAAGUGCUUC	2945	3 UTR	0.0020
Rgl1	NM_016846	10	3797	AAAGUGCUUC	3806	3 UTR	0.0014
Slc22a23	NM_001033167	10	2721	AAAGUGCUUC	2730	3 UTR	0.0036
Snx8	NM_172277	10	2552	AAAGUGCUUC	2561	3 UTR	0.0012
Stk11	NM_011492	10	1514	AAAGUGCUUC	1523	3 UTR	0.0002
Plekha7	NM_172743	10	3852	AAAGUGCUUC	3861	3 UTR	0.0006
Nos2	NM_010927	10	3904	AAAGUGCUUC	3913	3 UTR	0.0004
Nxph2	NM_008752	10	1581	AAAGUGCUUC	1590	3 UTR	0.0015
Slc16a9	NM_025807	10	3171	AAAGUGCUUC	3180	3 UTR	0.0018
Cry2	NM_009963	10	3897	AAAGUGCUUC	3906	3 UTR	0.0021
Gda	NM_010266	10	3131	AAAGUGCUUC	3140	3 UTR	0.0037
Pak7	NM_172858	9	3051	AAAGUGCUU	3059	3 UTR	0.0081
Tiam2	NM_011878	9	5994	AAAGUGCUU	6002	3 UTR	0.0026
Phf6	NM_027642	9	3239	AAAGUGCUU	3247	3 UTR	0.0117
St6galnac2	NM_009180	9	1254	AAAGUGCUU	1262	3 UTR	0.0031
Eda	NM_010099	9	3101	AAAGUGCUU	3109	3 UTR	0.0137
AC129021.4-202	NM_001081217	9	2570	AAAGUGCUU	2578	3 UTR	0.0154
Elovl6	NM_130450	9	3455	AAAGUGCUU	3463	3 UTR	0.0189

Ubfd1	NM_138589	9	1193	AAAGUGCUU	1201	3 UTR	0.0138
Ubxn2b	NM_026534	9	3169	AAAGUGCUU	3177	3 UTR	0.0141
Gtf2h2	NM_022011	9	1446	AAAGUGCUU	1454	3 UTR	0.0013
Pappa	NM_021362	9	7309	AAAGUGCUU	7317	3 UTR	0.0218
Wdr60	NM_146039	9	3482	AAAGUGCUU	3490	3 UTR	0.0022
Rs1	NM_011302	9	942	AAAGUGCUU	950	3 UTR	0.0189
Rock2	NM_009072	9	7286	AAAGUGCUU	7294	3 UTR	0.0138
App	NM_007471	9	2960	AAAGUGCUU	2968	3 UTR	0.0035
Ccnd2	NM_009829	9	2132	AAAGUGCUU	2140	3 UTR	0.0175
Lypd6	NM_177139	9	2570	AAAGUGCUU	2578	3 UTR	0.0106
Syap1	NM_025932	9	1657	AAAGUGCUU	1665	3 UTR	0.0039
Hrbl	NM_178162	9	2147	AAAGUGCUU	2155	3 UTR	0.0035
Rab22a	NM_024436	9	1312	AAAGUGCUU	1320	3 UTR	0.0040
Map3k14	NM_016896	9	3751	AAAGUGCUU	3759	3 UTR	0.0048
G6pc	NM_008061	9	2172	AAAGUGCUU	2180	3 UTR	0.0042
Slc24a2	NM_172426	9	7281	AAAGUGCUU	7289	3 UTR	0.0309
Ano5	NM_177694	9	6557	AAAGUGCUU	6565	3 UTR	0.0184
Gpr157	NM_177366	9	3667	AAAGUGCUU	3675	3 UTR	0.0133
Rpe	NM_025683	9	1652	AAAGUGCUU	1660	3 UTR	0.0073
Magi3	NM_133853	9	3671	AAAGUGCUU	3679	3 UTR	0.0032
Tollip	NM_023764	9	2405	AAAGUGCUU	2413	3 UTR	0.0106
Usp29	NM_021323	9	5302	AAAGUGCUU	5310	3 UTR	0.0131
Col17a1	NM_007732	9	5378	AAAGUGCUU	5386	3 UTR	0.0033
Slc6a15	NM_175328	9	2865	AAAGUGCUU	2873	3 UTR	0.0039
Helb	NM_080446	9	4339	AAAGUGCUU	4347	3 UTR	0.0046
Pld5	NM_176916	9	3557	AAAGUGCUU	3565	3 UTR	0.0058
Zfp800	NM_001081678	9	2870	AAAGUGCUU	2878	3 UTR	0.0066
Ift88	NM_009376	9	2814	AAAGUGCUU	2822	3 UTR	0.0018
Iqck	NM_001081446	9	1000	AAAGUGCUU	1008	3 UTR	0.0044
Lhx6	NM_001083125	9	3256	AAAGUGCUU	3264	3 UTR	0.0078
Pcdh7	NM_018764	9	4863	AAAGUGCUU	4871	3 UTR	0.0050
Col19a1	NM_007733	9	10521	AAAGUGCUU	10529	3 UTR	0.0264
Cdca7	NM_025866	9	1529	AAAGUGCUU	1537	3 UTR	0.0043
Rbl1	NM_011249	9	3292	AAAGUGCUU	3300	3 UTR	0.0062
Lpgat1	NM_172266	9	2976	AAAGUGCUU	2984	3 UTR	0.0216
Tmed8	NM_001033475	9	5597	AAAGUGCUU	5605	3 UTR	0.0233
Calb1	NM_009788	9	2237	AAAGUGCUU	2245	3 UTR	0.0074
6430573F11Rik	NM_176952	9	1522	AAAGUGCUU	1530	3 UTR	0.0016
Tmub2	NM_028076	9	1648	AAAGUGCUU	1656	3 UTR	0.0038
Uhrf1bp1	NM_001080769	9	6693	AAAGUGCUU	6701	3 UTR	0.0153
Unk	NM_172569	9	2713	AAAGUGCUU	2721	3 UTR	0.0046
Slc25a40	NM_178766	9	2551	AAAGUGCUU	2559	3 UTR	0.0057
Jazf1	NM_173406	9	1645	AAAGUGCUU	1653	3 UTR	0.0054
Mga	NM_013720	9	13449	AAAGUGCUU	13457	3 UTR	0.0175
Mink1	NM_001045959	9	4700	AAAGUGCUU	4708	3 UTR	0.0029
Dnase1l3	NM_007870	9	3435	AAAGUGCUU	3443	3 UTR	0.0098
C77080	NM_001033189	9	3692	AAAGUGCUU	3700	3 UTR	0.0071
Cngb3	NM_013927	9	2785	AAAGUGCUU	2793	3 UTR	0.0096
Hoxa1	NM_010449	9	1824	AAAGUGCUU	1832	3 UTR	0.0052
Bpi	NM_008325	9	2363	AAAGUGCUU	2371	3 UTR	0.0046
Mtf1	NM_008636	9	5357	AAAGUGCUU	5365	3 UTR	0.0206
Mecp2	NM_001081979	9	6475	AAAGUGCUU	6483	3 UTR	0.0323
Ankrd52	NM_172790	9	6007	AAAGUGCUU	6015	3 UTR	0.0125
Dcun1d4	NM_178896	9	1812	AAAGUGCUU	1820	3 UTR	0.0118
Nudt3	NM_019837	9	991	AAAGUGCUU	999	3 UTR	0.0053
Lace1	NM_145743	9	2036	AAAGUGCUU	2044	3 UTR	0.0035
Rgmb	NM_178615	9	2026	AAAGUGCUU	2034	3 UTR	0.0034
Impg2	NM_174876	9	4845	AAAGUGCUU	4853	3 UTR	0.0115
Sf3b1	NM_031179	9	5519	AAAGUGCUU	5527	3 UTR	0.0082
Zdhhc8	NM_172151	9	4417	AAAGUGCUU	4425	3 UTR	0.0094
Ikbkg	NM_178590	9	4261	AAAGUGCUU	4269	3 UTR	0.0120
F2rl2	NM_010170	9	2154	AAAGUGCUU	2162	3 UTR	0.0046
Rb1cc1	NM_009826	9	5851	AAAGUGCUU	5859	3 UTR	0.0050

Lmo3	NM_207222	9	1327	AAAGUGCUU	1335	3 UTR	0.0056
Nkapl	NM_025719	9	1347	AAAGUGCUU	1355	3 UTR	0.0006
Fyco1	NM_148925	9	7576	AAAGUGCUU	7584	3 UTR	0.0120
Wdr37	NM_001039388	9	2473	AAAGUGCUU	2481	3 UTR	0.0108
Ezh1	NM_007970	9	3284	AAAGUGCUU	3292	3 UTR	0.0069
Il6ra	NM_010559	9	1888	AAAGUGCUU	1896	3 UTR	0.0070
Cfl2	NM_007688	9	1125	AAAGUGCUU	1133	3 UTR	0.0088
Atad2	NM_027435	9	4516	AAAGUGCUU	4524	3 UTR	0.0056
Pdk4	NM_013743	9	1856	AAAGUGCUU	1864	3 UTR	0.0080
Rassf2	NM_175445	9	4589	AAAGUGCUU	4597	3 UTR	0.0135
Casc4	NM_177054	9	2562	AAAGUGCUU	2570	3 UTR	0.0101
Mterfd2	NM_178051	9	1137	AAAGUGCUU	1145	3 UTR	0.0085
Slc41a2	NM_177388	9	3061	AAAGUGCUU	3069	3 UTR	0.0092
Pde3b	NM_011055	9	3746	AAAGUGCUU	3754	3 UTR	0.0065
Trem1	NM_021406	9	1889	AAAGUGCUU	1897	3 UTR	0.0086
AA881470	NM_181066	9	3029	AAAGUGCUU	3037	3 UTR	0.0028
Itfg1	NM_028007	9	2039	AAAGUGCUU	2047	3 UTR	0.0013
Eif2ak2	NM_011163	9	4106	AAAGUGCUU	4114	3 UTR	0.0098
Tmem65	NM_175212	9	2920	AAAGUGCUU	2928	3 UTR	0.0100
Mmp24	NM_010808	9	3682	AAAGUGCUU	3690	3 UTR	0.0088
Lmbr1	NM_020295	9	2745	AAAGUGCUU	2753	3 UTR	0.0124
P2rx4	NM_011026	9	1817	AAAGUGCUU	1825	3 UTR	0.0028
Irak4	NM_029926	9	1549	AAAGUGCUU	1557	3 UTR	0.0038
Vldlr	NM_013703	9	3185	AAAGUGCUU	3193	3 UTR	0.0018
Znfx1	NM_001033196	9	6448	AAAGUGCUU	6456	3 UTR	0.0036
AB041550	NM_021418	9	1262	AAAGUGCUU	1270	3 UTR	0.0041
2010321M09Rik	NM_001077631	9	2843	AAAGUGCUU	2851	3 UTR	0.0033
Zfp26	NM_011753	9	6079	AAAGUGCUU	6087	3 UTR	0.0303
Zfp830	NM_025884	9	2278	AAAGUGCUU	2286	3 UTR	0.0082
March5	NM_027314	9	1479	AAAGUGCUU	1487	3 UTR	0.0024
Cc2d1a	NM_145970	9	3127	AAAGUGCUU	3135	3 UTR	0.0018
Ramp1	NM_016894	9	1678	AAAGUGCUU	1686	3 UTR	0.0070
St6galnac6	NM_001025311	9	2074	AAAGUGCUU	2082	3 UTR	0.0049
Pgbd5	NM_171824	9	2652	AAAGUGCUU	2660	3 UTR	0.0056
Lrp11	NM_172784	9	2708	AAAGUGCUU	2716	3 UTR	0.0064
Zfp708	NM_001012325	9	2133	AAAGUGCUU	2141	3 UTR	0.0030
Liph	NM_001083894	9	1775	AAAGUGCUU	1783	3 UTR	0.0087
Ogfod2	NM_025671	9	1538	AAAGUGCUU	1546	3 UTR	0.0019
Slc35b4	NM_021435	9	1597	AAAGUGCUU	1605	3 UTR	0.0095
Pgr15l	NM_001033361	9	4028	AAAGUGCUU	4036	3 UTR	0.0141
Ddhd1	NM_001042719	9	2996	AAAGUGCUU	3004	3 UTR	0.0088
Rgs8	NM_026380	9	3529	AAAGUGCUU	3537	3 UTR	0.0181
Map3k2	NM_011946	9	2650	AAAGUGCUU	2658	3 UTR	0.0315
Nek9	NM_145138	9	3229	AAAGUGCUU	3237	3 UTR	0.0088
Fgd5	NM_172731	9	4630	AAAGUGCUU	4638	3 UTR	0.0054
Ube2q2	NM_180600	9	2435	AAAGUGCUU	2443	3 UTR	0.0058
Kif26b	NM_177757	9	5100	AAAGUGCUU	5108	3 UTR	0.0025
Maoa	NM_173740	9	2641	AAAGUGCUU	2649	3 UTR	0.0089
Lif	NM_008501	9	2984	AAAGUGCUU	2992	3 UTR	0.0121
Ccnd1	NM_007631	9	2043	AAAGUGCUU	2051	3 UTR	0.0102
Tmod2	NM_001038710	9	2051	AAAGUGCUU	2059	3 UTR	0.0318
Srrp	NM_177774	9	1248	AAAGUGCUU	1256	3 UTR	0.0078
St3gal1	NM_009177	9	5555	AAAGUGCUU	5563	3 UTR	0.0143
CT025653.7-201	NM_172765	9	1810	AAAGUGCUU	1818	3 UTR	0.0269
Bbs12	NM_001008502	9	2329	AAAGUGCUU	2337	3 UTR	0.0011
Mknk2	NM_021462	9	2953	AAAGUGCUU	2961	3 UTR	0.0068
Star	NM_011485	9	2884	AAAGUGCUU	2892	3 UTR	0.0117
E130009J12Rik	NM_001039104	9	5230	AAAGUGCUU	5238	3 UTR	0.0025
Irf9	NM_008394	9	1701	AAAGUGCUU	1709	3 UTR	0.0039
Clock	NM_007715	9	6795	AAAGUGCUU	6803	3 UTR	0.0171
Pbx3	NM_016768	9	1607	AAAGUGCUU	1615	3 UTR	0.0037
Rnf6	NM_028774	9	2614	AAAGUGCUU	2622	3 UTR	0.0038
Mmp23	NM_011985	9	1418	AAAGUGCUU	1426	3 UTR	0.0002

Slc18a1	NM_153054	9	2608	AAAGUGCUU	2616	3 UTR	0.0041
Tanc1	NM_198294	9	7529	AAAGUGCUU	7537	3 UTR	0.0077
Lrrc3	NM_145152	9	1157	AAAGUGCUU	1165	3 UTR	0.0124
Rapgefl1	NM_001080925	9	2409	AAAGUGCUU	2417	3 UTR	0.0069
Flt1	NM_010228	9	5386	AAAGUGCUU	5394	3 UTR	0.0077
Ugp2	NM_139297	9	1801	AAAGUGCUU	1809	3 UTR	0.0033
4932442K08Rik	NM_024203	9	3877	AAAGUGCUU	3885	3 UTR	0.0070
C1qa	NM_007572	9	1041	AAAGUGCUU	1049	3 UTR	0.0009
Asb8	NM_030121	9	1960	AAAGUGCUU	1968	3 UTR	0.0044
Zfp575	NM_001033205	9	1686	AAAGUGCUU	1694	3 UTR	0.0063
Abca1	NM_013454	9	10035	AAAGUGCUU	10043	3 UTR	0.0120
Arl4c	NM_177305	9	3323	AAAGUGCUU	3331	3 UTR	0.0109
Ss18l1	NM_178750	9	3709	AAAGUGCUU	3717	3 UTR	0.0109
Camk2n1	NM_025451	9	370	AAAGUGCUU	378	3 UTR	0.0127
Olfm3	NM_153458	9	1903	AAAGUGCUU	1911	3 UTR	0.0091
Vsx1	NM_054068	9	2177	AAAGUGCUU	2185	3 UTR	0.0088
Midn	NM_021565	9	2365	AAAGUGCUU	2373	3 UTR	0.0066
Klhl21	NM_001033352	9	3482	AAAGUGCUU	3490	3 UTR	0.0078
Crot	NM_023733	9	2350	AAAGUGCUU	2358	3 UTR	0.0031
Zfp810	NM_145612	9	2015	AAAGUGCUU	2023	3 UTR	0.0056
Prdx6-rs1	NM_177256	9	1887	AAAGUGCUU	1895	3 UTR	0.0070
Aifm1	NM_012019	9	1948	AAAGUGCUU	1956	3 UTR	0.0007
Nr2c1	NM_011629	9	2601	AAAGUGCUU	2609	3 UTR	0.0071
Zfp408	NM_001033451	9	3286	AAAGUGCUU	3294	3 UTR	0.0049
Unkl	NM_028789	9	1143	AAAGUGCUU	1151	3 UTR	0.0098
Snf1lk	NM_010831	9	4106	AAAGUGCUU	4114	3 UTR	0.0077
1810043G02Rik	NM_026431	9	1043	AAAGUGCUU	1051	3 UTR	0.0034
Map6d1	NM_198599	9	3251	AAAGUGCUU	3259	3 UTR	0.0101
1700021K19Rik	NM_172615	9	4946	AAAGUGCUU	4954	3 UTR	0.0085
AC160545.8	NM_001033250	9	1915	AAAGUGCUU	1923	3 UTR	0.0089
Napepld	NM_178728	9	2825	AAAGUGCUU	2833	3 UTR	0.0088
Mtm1	NM_019926	9	2130	AAAGUGCUU	2138	3 UTR	0.0055
Atp2b2	NM_001036684	9	5728	AAAGUGCUU	5736	3 UTR	0.0110
Kif5c	NM_008449	9	5199	AAAGUGCUU	5207	3 UTR	0.0138
AC166360.4-201	NM_199252	9	1997	AAAGUGCUU	2005	3 UTR	0.0038
Spic	NM_011461	9	930	AAAGUGCUU	938	3 UTR	0.0015
Chrna2	NM_144803	9	2604	AAAGUGCUU	2612	3 UTR	0.0071
Zfp597	NM_001033159	9	1624	AAAGUGCUU	1632	3 UTR	0.0155
Slc6a9	NM_008135	9	2445	AAAGUGCUU	2453	3 UTR	0.0040
Ttc9	NM_001033149	9	1975	AAAGUGCUU	1983	3 UTR	0.0051
Rab30	NM_029494	9	1259	AAAGUGCUU	1267	3 UTR	0.0048
Zbtb43	NM_027947	9	3368	AAAGUGCUU	3376	3 UTR	0.0129
Pip4k2a	NM_008845	9	3451	AAAGUGCUU	3459	3 UTR	0.0081
Rutbc2	NM_172718	9	4177	AAAGUGCUU	4185	3 UTR	0.0068
Ptpn21	NM_011877	9	4611	AAAGUGCUU	4619	3 UTR	0.0069
Dars	NM_145507	9	545	AAAGUGCUU	553	3 UTR	0.0009
Pkd2l2	NM_016927	9	2278	AAAGUGCUU	2286	3 UTR	0.0022
1300010M03Rik	NM_026501	8	2269	AAAGUGCU	2276	3 UTR	0.0248
AA409316	NM_134087	8	4248	AAAGUGCU	4255	3 UTR	0.0114
Gnpnat1	NM_019425	8	1814	AAAGUGCU	1821	3 UTR	0.0288
Sh3glb1	NM_019464	8	2632	AAAGUGCU	2639	3 UTR	0.0386
Hdac9	NM_024124	8	3599	AAAGUGCU	3606	3 UTR	0.0354
Dpp3	NM_133803	8	2386	AAAGUGCU	2393	3 UTR	0.0064
Dusp2	NM_010090	8	1547	AAAGUGCU	1554	3 UTR	0.0090
Ankmy2	NM_146033	8	2043	AAAGUGCU	2050	3 UTR	0.0135
Mll3	NM_001081383	8	15171	AAAGUGCU	15178	3 UTR	0.0282
1110018G07Rik	NM_178065	8	4895	AAAGUGCU	4902	3 UTR	0.0355
Tomm7	NM_025394	8	995	AAAGUGCU	1002	3 UTR	0.0134
Fndc3a	NM_207636	8	5142	AAAGUGCU	5149	3 UTR	0.0338
Tbc1d8b	NM_001081499	8	4596	AAAGUGCU	4603	3 UTR	0.0356
Mapk9	NM_207692	8	1903	AAAGUGCU	1910	3 UTR	0.0420
Rsrc2	NM_001005525	8	2141	AAAGUGCU	2148	3 UTR	0.0113
Frmd4a	NM_172475	8	3441	AAAGUGCU	3448	3 UTR	0.0432

Cbx4	NM_007625	8	4684	AAAGUGCU	4691	3 UTR	0.0496
Ssx2ip	NM_138744	8	2362	AAAGUGCU	2369	3 UTR	0.0198
Vsx2	NM_007701	8	1629	AAAGUGCU	1636	3 UTR	0.0304
Tgfbrap1	NM_001013025	8	4278	AAAGUGCU	4285	3 UTR	0.0362
Metap1	NM_175224	8	1430	AAAGUGCU	1437	3 UTR	0.0208
Fgd4	NM_139232	8	2705	AAAGUGCU	2712	3 UTR	0.0092
Rhov	NM_145530	8	1036	AAAGUGCU	1043	3 UTR	0.0134
Dpysl5	NM_023047	8	2370	AAAGUGCU	2377	3 UTR	0.0462
Sipa1l3	NM_001081028	8	6333	AAAGUGCU	6340	3 UTR	0.0266
Txnip	NM_001009935	8	2184	AAAGUGCU	2191	3 UTR	0.0201
Adam7	NM_007402	8	3365	AAAGUGCU	3372	3 UTR	0.0164
Atxn7l1	NM_001033436	8	2657	AAAGUGCU	2664	3 UTR	0.0033
Crk	NM_133656	8	1691	AAAGUGCU	1698	3 UTR	0.0388
Map3k1	NM_011945	8	4804	AAAGUGCU	4811	3 UTR	0.0369
Cybb	NM_007807	8	1958	AAAGUGCU	1965	3 UTR	0.0442
Reep3	NM_178606	8	1319	AAAGUGCU	1326	3 UTR	0.0333
Ear6	NM_053111	8	546	AAAGUGCU	553	3 UTR	0.0031
Camta1	NM_001081557	8	5962	AAAGUGCU	5969	3 UTR	0.0491
Slc2a4	NM_009204	8	1798	AAAGUGCU	1805	3 UTR	0.0164
Ccnj	NM_172839	8	2366	AAAGUGCU	2373	3 UTR	0.0370
Klhl23	NM_177784	8	2439	AAAGUGCU	2446	3 UTR	0.0398
Rps6ka5	NM_153587	8	3820	AAAGUGCU	3827	3 UTR	0.0251
Tmem97	NM_133706	8	939	AAAGUGCU	946	3 UTR	0.0109
Ccdc55	NM_001012309	8	1854	AAAGUGCU	1861	3 UTR	0.0219
Tmem123	NM_133739	8	2421	AAAGUGCU	2428	3 UTR	0.0322
Dgke	NM_019505	8	4805	AAAGUGCU	4812	3 UTR	0.0469
Zfp362	NM_001081098	8	1664	AAAGUGCU	1671	3 UTR	0.0217
Rreb1	NM_001039188	8	6743	AAAGUGCU	6750	3 UTR	0.0448
2310014L17Rik	NM_029809	8	1418	AAAGUGCU	1425	3 UTR	0.0327
0610007L01Rik	NM_001081394	8	2448	AAAGUGCU	2455	3 UTR	0.0369
Sfrs12	NM_172592	8	3608	AAAGUGCU	3615	3 UTR	0.0293
Dmtf1	NM_011806	8	3237	AAAGUGCU	3244	3 UTR	0.0178
Gpr88	NM_022427	8	2358	AAAGUGCU	2365	3 UTR	0.0279
Arhgef18	NM_133962	8	4868	AAAGUGCU	4875	3 UTR	0.0310
Brms1l	NM_001037756	8	1663	AAAGUGCU	1670	3 UTR	0.0220
Acaa1a	NM_130864	8	1604	AAAGUGCU	1611	3 UTR	0.0040
Mcfd2	NM_139295	8	628	AAAGUGCU	635	3 UTR	0.0192
Epas1	NM_010137	8	4817	AAAGUGCU	4824	3 UTR	0.0346
Fbxl4	NM_172988	8	2339	AAAGUGCU	2346	3 UTR	0.0048
Ihpk1	NM_013785	8	3593	AAAGUGCU	3600	3 UTR	0.0422
Nrsn2	NM_001009948	8	1135	AAAGUGCU	1142	3 UTR	0.0121
Bace2	NM_019517	8	3569	AAAGUGCU	3576	3 UTR	0.0318
Osm	NM_001013365	8	1365	AAAGUGCU	1372	3 UTR	0.0154
Atg7	NM_028835	8	3022	AAAGUGCU	3029	3 UTR	0.0138
Pfn	NM_019410	8	928	AAAGUGCU	935	3 UTR	0.0206
Tmem41b	NM_153525	8	1550	AAAGUGCU	1557	3 UTR	0.0388
Tusc2	NM_019742	8	836	AAAGUGCU	843	3 UTR	0.0181
Gabpb1	NM_207669	8	2137	AAAGUGCU	2144	3 UTR	0.0199
Pcdhb17	NM_053142	8	2761	AAAGUGCU	2768	3 UTR	0.0113
Erbb2ip	NM_001005868	8	6321	AAAGUGCU	6328	3 UTR	0.0289
Hmbox1	NM_177338	8	2868	AAAGUGCU	2875	3 UTR	0.0203
Abcd2	NM_011994	8	4317	AAAGUGCU	4324	3 UTR	0.0464
Jup	NM_010593	8	2555	AAAGUGCU	2562	3 UTR	0.0133
Dirc2	NM_153550	8	2251	AAAGUGCU	2258	3 UTR	0.0448
Fto	NM_011936	8	3403	AAAGUGCU	3410	3 UTR	0.0304
Ubxn2a	NM_145441	8	1748	AAAGUGCU	1755	3 UTR	0.0234
Inoc1	NM_026574	8	5142	AAAGUGCU	5149	3 UTR	0.0200
Nol8	NM_001081350	8	3897	AAAGUGCU	3904	3 UTR	0.0088
Ebna1bp2	NM_026932	8	2368	AAAGUGCU	2375	3 UTR	0.0286
Tspan11	NM_026743	8	1463	AAAGUGCU	1470	3 UTR	0.0473
Chn2	NM_023543	8	1934	AAAGUGCU	1941	3 UTR	0.0241
Synpo2l	NM_175132	8	3316	AAAGUGCU	3323	3 UTR	0.0134
Wee1	NM_009516	8	2725	AAAGUGCU	2732	3 UTR	0.0179

Oxr1	NM_130885	8	3808	AAAGUGCU	3815	3 UTR	0.0264
Gls	NM_001081081	8	2895	AAAGUGCU	2902	3 UTR	0.0405
Tbc1d2	NM_198664	8	3088	AAAGUGCU	3095	3 UTR	0.0207
Kif13a	NM_010617	8	5503	AAAGUGCU	5510	3 UTR	0.0229
Sgcb	NM_011890	8	2975	AAAGUGCU	2982	3 UTR	0.0410
Shc4	NM_199022	8	3158	AAAGUGCU	3165	3 UTR	0.0325
Cadm2	NM_178721	8	3803	AAAGUGCU	3810	3 UTR	0.0304
Atp1a2	NM_178405	8	5255	AAAGUGCU	5262	3 UTR	0.0446
Ttc9c	NM_027412	8	1659	AAAGUGCU	1666	3 UTR	0.0399
Acpl2	NM_153420	8	2374	AAAGUGCU	2381	3 UTR	0.0203
Gns	NM_029364	8	3699	AAAGUGCU	3706	3 UTR	0.0318
Fndc3b	NM_173182	8	6130	AAAGUGCU	6137	3 UTR	0.0466
5730593F17Rik	NM_172543	8	1519	AAAGUGCU	1526	3 UTR	0.0138
Ormdl3	NM_025661	8	959	AAAGUGCU	966	3 UTR	0.0214
Ogfrl1	NM_001081079	8	4105	AAAGUGCU	4112	3 UTR	0.0495
E130311K13Rik	NM_177856	8	952	AAAGUGCU	959	3 UTR	0.0097
Arid4b	NM_198122	8	3991	AAAGUGCU	3998	3 UTR	0.0281
Mgat5b	NM_172948	8	3047	AAAGUGCU	3054	3 UTR	0.0194
Znhit6	NM_001081094	8	2214	AAAGUGCU	2221	3 UTR	0.0192
Zim1	NM_011769	8	2733	AAAGUGCU	2740	3 UTR	0.0227
Acaa1b	NM_146230	8	1482	AAAGUGCU	1489	3 UTR	0.0045
Icos	NM_017480	8	1909	AAAGUGCU	1916	3 UTR	0.0211
A630047E20Rik	NM_173032	8	2781	AAAGUGCU	2788	3 UTR	0.0100
Usp46	NM_177561	8	2764	AAAGUGCU	2771	3 UTR	0.0300
Mycn	NM_008709	8	2543	AAAGUGCU	2550	3 UTR	0.0138
Stk38l	NM_172734	8	3285	AAAGUGCU	3292	3 UTR	0.0464
Josd1	NM_028792	8	2777	AAAGUGCU	2784	3 UTR	0.0275
Gfpt2	NM_013529	8	2797	AAAGUGCU	2804	3 UTR	0.0130
Aytl1	NM_173014	8	2439	AAAGUGCU	2446	3 UTR	0.0160
Smc2	NM_008017	8	3841	AAAGUGCU	3848	3 UTR	0.0244
F3	NM_010171	8	1613	AAAGUGCU	1620	3 UTR	0.0123
Mupcdh	NM_028069	8	2134	AAAGUGCU	2141	3 UTR	0.0010
Slc40a1	NM_016917	8	3168	AAAGUGCU	3175	3 UTR	0.0202
Elavl2	NM_207685	8	3440	AAAGUGCU	3447	3 UTR	0.0378
Slc5a7	NM_022025	8	1981	AAAGUGCU	1988	3 UTR	0.0440
Fmo1	NM_010231	8	2117	AAAGUGCU	2124	3 UTR	0.0066
Bcl2l14	NM_025778	8	2120	AAAGUGCU	2127	3 UTR	0.0149
Arhgef4	NM_183019	8	1815	AAAGUGCU	1822	3 UTR	0.0144
Map3k11	NM_022012	8	3623	AAAGUGCU	3630	3 UTR	0.0097
Mtmr7	NM_001040699	8	3194	AAAGUGCU	3201	3 UTR	0.0210
Cycs	NM_007808	8	1527	AAAGUGCU	1534	3 UTR	0.0400
D030056L22Rik	NM_177640	8	1325	AAAGUGCU	1332	3 UTR	0.0160
Rbp3	NM_015745	8	4213	AAAGUGCU	4220	3 UTR	0.0226
Zfand2a	NM_133349	8	1810	AAAGUGCU	1817	3 UTR	0.0360
Arhgef10	NM_172751	8	5334	AAAGUGCU	5341	3 UTR	0.0191
Pdzd3	NM_133226	8	2117	AAAGUGCU	2124	3 UTR	0.0102
Ssh3	NM_198113	8	2478	AAAGUGCU	2485	3 UTR	0.0104
Itgb8	NM_177290	8	2993	AAAGUGCU	3000	3 UTR	0.0048
Rbbp7	NM_009031	8	1729	AAAGUGCU	1736	3 UTR	0.0102
Chst10	NM_145142	8	1826	AAAGUGCU	1833	3 UTR	0.0247
AC160757.3-202	NM_028494	8	2307	AAAGUGCU	2314	3 UTR	0.0254
Neu1	NM_010893	8	1451	AAAGUGCU	1458	3 UTR	0.0076
Asah3l	NM_139306	8	1834	AAAGUGCU	1841	3 UTR	0.0493
Hcrtr2	NM_198962	8	2369	AAAGUGCU	2376	3 UTR	0.0341
Rell1	NM_145923	8	2580	AAAGUGCU	2587	3 UTR	0.0339
Clec5a	NM_001038604	8	3665	AAAGUGCU	3672	3 UTR	0.0457
Hmg20a	NM_025812	8	1926	AAAGUGCU	1933	3 UTR	0.0364
Clic4	NM_013885	8	936	AAAGUGCU	943	3 UTR	0.0467
Zfp697	NM_172863	8	2968	AAAGUGCU	2975	3 UTR	0.0495
Amz2	NM_025275	8	1683	AAAGUGCU	1690	3 UTR	0.0216
Foxn4	NM_148935	8	2275	AAAGUGCU	2282	3 UTR	0.0200
Tmem87b	NM_028248	8	2520	AAAGUGCU	2527	3 UTR	0.0437
Tiam1	NM_009384	8	6529	AAAGUGCU	6536	3 UTR	0.0302

Cntnap1	NM_016782	8	4873	AAAGUGCU	4880	3 UTR	0.0161
Sult1a1	NM_133670	8	1030	AAAGUGCU	1037	3 UTR	0.0093
Nufip1	NM_013745	8	3183	AAAGUGCU	3190	3 UTR	0.0371
Wfs1	NM_011716	8	3129	AAAGUGCU	3136	3 UTR	0.0117
Rbbp9	NM_015754	8	775	AAAGUGCU	782	3 UTR	0.0232
Tal1	NM_011527	8	3915	AAAGUGCU	3922	3 UTR	0.0452
Cygb	NM_030206	8	996	AAAGUGCU	1003	3 UTR	0.0206
Cxadr	NM_009988	8	1303	AAAGUGCU	1310	3 UTR	0.0081
Lhx8	NM_010713	8	1643	AAAGUGCU	1650	3 UTR	0.0103
Aldh9a1	NM_019993	8	2716	AAAGUGCU	2723	3 UTR	0.0156
Npas3	NM_013780	8	2829	AAAGUGCU	2836	3 UTR	0.0462
Lrrc19	NM_175305	8	1728	AAAGUGCU	1735	3 UTR	0.0236
Ppp6c	NM_024209	8	1097	AAAGUGCU	1104	3 UTR	0.0086
E2f2	NM_177733	8	3060	AAAGUGCU	3067	3 UTR	0.0451
6030458C11Rik	NM_029998	8	1766	AAAGUGCU	1773	3 UTR	0.0344
Macrod2	NM_028387	8	1601	AAAGUGCU	1608	3 UTR	0.0420
Naip5	NM_010870	8	4948	AAAGUGCU	4955	3 UTR	0.0143
Nfatc2ip	NM_010900	8	2629	AAAGUGCU	2636	3 UTR	0.0319
Daglb	NM_144915	8	2785	AAAGUGCU	2792	3 UTR	0.0160
Slc5a10	NM_001033227	8	2224	AAAGUGCU	2231	3 UTR	0.0034
Acox1	NM_015729	8	2743	AAAGUGCU	2750	3 UTR	0.0262
Taf12	NM_025579	8	1240	AAAGUGCU	1247	3 UTR	0.0101
Plscr4	NM_178711	8	3003	AAAGUGCU	3010	3 UTR	0.0257
Pcdha10	NM_054072	8	5100	AAAGUGCU	5107	3 UTR	0.0360
Armcx6	NM_001007578	8	1352	AAAGUGCU	1359	3 UTR	0.0113
Psmf1	NM_212446	8	2846	AAAGUGCU	2853	3 UTR	0.0385
Camta2	NM_178116	8	4412	AAAGUGCU	4419	3 UTR	0.0111
E2f5	NM_007892	8	1598	AAAGUGCU	1605	3 UTR	0.0092
Cep76	NM_001081073	8	2857	AAAGUGCU	2864	3 UTR	0.0276
Dapk2	NM_010019	8	1342	AAAGUGCU	1349	3 UTR	0.0085
Elk4	NM_007923	8	3397	AAAGUGCU	3404	3 UTR	0.0305
Ptcd1	NM_133735	8	2283	AAAGUGCU	2290	3 UTR	0.0126
Kpna2	NM_010655	8	1894	AAAGUGCU	1901	3 UTR	0.0044
Wdr45	NM_172372	8	1374	AAAGUGCU	1381	3 UTR	0.0054
Coq10b	NM_001039710	8	1327	AAAGUGCU	1334	3 UTR	0.0138
Ank2	NM_178655	8	5334	AAAGUGCU	5341	3 UTR	0.0355
C530043G21Rik	NM_145413	8	2935	AAAGUGCU	2942	3 UTR	0.0424
Rab11fip5	NM_177466	8	4066	AAAGUGCU	4073	3 UTR	0.0314
2310002B06Rik	NM_181649	8	1156	AAAGUGCU	1163	3 UTR	0.0468
5730472N09Rik	NM_175392	8	2985	AAAGUGCU	2992	3 UTR	0.0214
Foxj2	NM_021899	8	3756	AAAGUGCU	3763	3 UTR	0.0381
Exoc1	NM_027270	8	3089	AAAGUGCU	3096	3 UTR	0.0082
Sdhd	NM_025848	8	518	AAAGUGCU	525	3 UTR	0.0112
Trpv6	NM_022413	8	2635	AAAGUGCU	2642	3 UTR	0.0067
Vhlh	NM_009507	8	1227	AAAGUGCU	1234	3 UTR	0.0310
Fgf4	NM_010202	8	780	AAAGUGCU	787	3 UTR	0.0345
Nrip3	NM_020610	8	3831	AAAGUGCU	3838	3 UTR	0.0465
Uevld	NM_001040695	8	2414	AAAGUGCU	2421	3 UTR	0.0463
Zfp213	NM_001033496	8	2420	AAAGUGCU	2427	3 UTR	0.0135
Traf4	NM_009423	8	3536	AAAGUGCU	3543	3 UTR	0.0390
Shisa3	NM_001033415	8	1385	AAAGUGCU	1392	3 UTR	0.0121
Cd53	NM_007651	8	2273	AAAGUGCU	2280	3 UTR	0.0292
Zfp488	NM_001013777	8	2517	AAAGUGCU	2524	3 UTR	0.0468
Tfip11	NM_018783	8	3323	AAAGUGCU	3330	3 UTR	0.0111
Wbscr27	NM_024479	8	2659	AAAGUGCU	2666	3 UTR	0.0297
Phf7	NM_027949	8	1810	AAAGUGCU	1817	3 UTR	0.0073
Rhobtb2	NM_153514	8	3351	AAAGUGCU	3358	3 UTR	0.0378
Zbtb9	NM_001005916	8	2404	AAAGUGCU	2411	3 UTR	0.0185
Gpr146	NM_001038703	8	1232	AAAGUGCU	1239	3 UTR	0.0443
Osbp2	NM_152818	8	3213	AAAGUGCU	3220	3 UTR	0.0208
Huwe1	NM_021523	8	14554	AAAGUGCU	14561	3 UTR	0.0158
Gpr63	NM_030733	8	2220	AAAGUGCU	2227	3 UTR	0.0103
Foxo1	NM_019739	8	2556	AAAGUGCU	2563	3 UTR	0.0383

Slain2	NM_153567	8	2289	AAAGUGCU	2296	3 UTR	0.0413
Trp53inp2	NM_178111	8	3333	AAAGUGCU	3340	3 UTR	0.0455
Parp3	NM_145619	8	1822	AAAGUGCU	1829	3 UTR	0.0133
Ear1	NM_007894	8	547	AAAGUGCU	554	3 UTR	0.0031
4121402D02Rik	NM_028722	8	3156	AAAGUGCU	3163	3 UTR	0.0366
Ear12	NM_007895	8	553	AAAGUGCU	560	3 UTR	0.0027
Ccdc137	NM_152807	8	1364	AAAGUGCU	1371	3 UTR	0.0281
Entpd4	NM_026174	8	2255	AAAGUGCU	2262	3 UTR	0.0144
Ear11	NM_053113	8	524	AAAGUGCU	531	3 UTR	0.0032
C1qb	NM_175484	8	3034	AAAGUGCU	3041	3 UTR	0.0272
Prps2	NM_026662	8	2331	AAAGUGCU	2338	3 UTR	0.0385
Rufy1	NM_172557	8	2334	AAAGUGCU	2341	3 UTR	0.0078
Il10rb	NM_008349	8	1379	AAAGUGCU	1386	3 UTR	0.0078
Nedd1	NM_008682	8	2882	AAAGUGCU	2889	3 UTR	0.0220
Slc25a27	NM_028711	8	1102	AAAGUGCU	1109	3 UTR	0.0257
Scn1a	NM_018733	8	7482	AAAGUGCU	7489	3 UTR	0.0316
Slc16a12	NM_172838	8	3275	AAAGUGCU	3282	3 UTR	0.0334
Atp11c	NM_001001798	8	4396	AAAGUGCU	4403	3 UTR	0.0369
Gdap1	NM_010267	8	2018	AAAGUGCU	2025	3 UTR	0.0416
Ube2j1	NM_019586	8	3435	AAAGUGCU	3442	3 UTR	0.0360
Pfkp	NM_019703	8	2480	AAAGUGCU	2487	3 UTR	0.0239
Plekhm1	NM_183034	8	4048	AAAGUGCU	4055	3 UTR	0.0272
Mr1	NM_008209	8	2390	AAAGUGCU	2397	3 UTR	0.0210
Tceb3	NM_013736	8	3235	AAAGUGCU	3242	3 UTR	0.0337
Alx4	NM_007442	8	1528	AAAGUGCU	1535	3 UTR	0.0057
Lactb2	NM_145381	8	1153	AAAGUGCU	1160	3 UTR	0.0146
Dgkq	NM_199011	8	3668	AAAGUGCU	3675	3 UTR	0.0261
Zfp280d	NM_146224	8	3344	AAAGUGCU	3351	3 UTR	0.0193
Tor1b	NM_133673	8	1102	AAAGUGCU	1109	3 UTR	0.0295
Ak3l1	NM_009647	8	2080	AAAGUGCU	2087	3 UTR	0.0495
Rbm11	NM_198302	8	1588	AAAGUGCU	1595	3 UTR	0.0289
1200013P24Rik	NM_029090	8	960	AAAGUGCU	967	3 UTR	0.0229
Pdia6	NM_027959	8	2086	AAAGUGCU	2093	3 UTR	0.0116
Mapk14	NM_011951	8	2880	AAAGUGCU	2887	3 UTR	0.0310
Camsap1l1	NM_001081360	8	5905	AAAGUGCU	5912	3 UTR	0.0406
Spinlw1	NM_029325	8	549	AAAGUGCU	556	3 UTR	0.0045
Sfrs17b	NM_001081956	8	5488	AAAGUGCU	5495	3 UTR	0.0417
Trim6	NM_001013616	8	2577	AAAGUGCU	2584	3 UTR	0.0333
Mul1	NM_026689	8	2002	AAAGUGCU	2009	3 UTR	0.0387
Polq	NM_029977	8	8012	AAAGUGCU	8019	3 UTR	0.0119
AC158970.3	NM_020507	8	2121	AAAGUGCU	2128	3 UTR	0.0368
Rexo1	NM_025852	8	5184	AAAGUGCU	5191	3 UTR	0.0221
Ppp3r1	NM_024459	8	945	AAAGUGCU	952	3 UTR	0.0312
Pdc	NM_024458	8	999	AAAGUGCU	1006	3 UTR	0.0063
Bhmt2	NM_022884	8	1817	AAAGUGCU	1824	3 UTR	0.0146
Rtn4rl1	NM_177708	8	2869	AAAGUGCU	2876	3 UTR	0.0253
Pnp1	NM_013632	8	2655	AAAGUGCU	2662	3 UTR	0.0269
Clip4	NM_030179	8	2452	AAAGUGCU	2459	3 UTR	0.0271
Aktip	NM_010241	8	1501	AAAGUGCU	1508	3 UTR	0.0162
Clcn7	NM_011930	8	2676	AAAGUGCU	2683	3 UTR	0.0236
Acsf2	NM_153807	8	2363	AAAGUGCU	2370	3 UTR	0.0182
March8	NM_027920	8	1871	AAAGUGCU	1878	3 UTR	0.0479
Dtl	NM_029766	8	3184	AAAGUGCU	3191	3 UTR	0.0282
4933407N01Rik	NM_025745	8	2940	AAAGUGCU	2947	3 UTR	0.0300
Cbara1	NM_144822	8	2260	AAAGUGCU	2267	3 UTR	0.0119
Kat2b	NM_020005	8	2583	AAAGUGCU	2590	3 UTR	0.0279
Ostm1	NM_172416	8	2787	AAAGUGCU	2794	3 UTR	0.0288
Prrx1	NM_011127	8	3402	AAAGUGCU	3409	3 UTR	0.0470
Chaf1a	NM_013733	8	3071	AAAGUGCU	3078	3 UTR	0.0072
Tslp	NM_021367	8	1064	AAAGUGCU	1071	3 UTR	0.0104
RP23-58H5.3	NM_172877	8	2633	AAAGUGCU	2640	3 UTR	0.0253
Nanos1	NM_178421	8	1183	AAAGUGCU	1190	3 UTR	0.0427
Col4a2	NM_009932	8	5927	AAAGUGCU	5934	3 UTR	0.0140

Bxdc1	NM_001042556	8	1350	AAAGUGCU	1357	3 UTR	0.0075
Uchl1	NM_011670	8	1015	AAAGUGCU	1022	3 UTR	0.0048
Fbxl2	NM_178624	8	3066	AAAGUGCU	3073	3 UTR	0.0282
Mfsd9	NM_172499	8	2748	AAAGUGCU	2755	3 UTR	0.0258
Pip4k2c	NM_054097	8	2034	AAAGUGCU	2041	3 UTR	0.0302
Klhl12	NM_153128	8	2051	AAAGUGCU	2058	3 UTR	0.0214
Lbxcor1	NM_172446	8	3548	AAAGUGCU	3555	3 UTR	0.0106
Osbpl5	NM_024289	8	2990	AAAGUGCU	2997	3 UTR	0.0160
C230093N12Rik	NM_153560	8	2947	AAAGUGCU	2954	3 UTR	0.0346
Rnf138	NM_207623	8	2558	AAAGUGCU	2565	3 UTR	0.0311
Mmaa	NM_133823	8	2462	AAAGUGCU	2469	3 UTR	0.0227
Casp12	NM_009808	8	2469	AAAGUGCU	2476	3 UTR	0.0197
Lig4	NM_176953	8	1493	AAAGUGCU	1500	3 UTR	0.0000
Sec14l4	NM_146013	8	1436	AAAGUGCU	1443	3 UTR	0.0253
Clec1a	NM_175526	8	2522	AAAGUGCU	2529	3 UTR	0.0325
Cdkn1a	NM_007669	8	1016	AAAGUGCU	1023	3 UTR	0.0201
Tgds	NM_029578	8	1474	AAAGUGCU	1481	3 UTR	0.0097
Prr16	NM_001081224	8	1021	AAAGUGCU	1028	3 UTR	0.0193
4930562D19Rik	NM_177616	8	4160	AAAGUGCU	4167	3 UTR	0.0384
Diablo	NM_023232	8	1501	AAAGUGCU	1508	3 UTR	0.0179
Mllt11	NM_019914	8	1972	AAAGUGCU	1979	3 UTR	0.0247
Centg1	NM_001033263	8	4395	AAAGUGCU	4402	3 UTR	0.0205
BC038286	NM_170755	8	2367	AAAGUGCU	2374	3 UTR	0.0130
Nat14	NM_201355	7	1126	AAAGUGC	1132	3 UTR	0.0333
Fgd1	NM_008001	7	3434	AAAGUGC	3440	3 UTR	0.0116
Tctex1d2	NM_025329	7	620	AAAGUGC	626	3 UTR	0.0072
Psd	NM_028627	7	3508	AAAGUGC	3514	3 UTR	0.0396
Galnt1	NM_013814	7	2245	AAAGUGC	2251	3 UTR	0.0471
6330527O06Rik	NM_029530	7	1292	AAAGUGC	1298	3 UTR	0.0439
AC090489.8	NM_001081040	7	2444	AAAGUGC	2450	3 UTR	0.0317
Afg3l2	NM_027130	7	3039	AAAGUGC	3045	3 UTR	0.0325
Trip10	NM_134125	7	1817	AAAGUGC	1823	3 UTR	0.0166
Arl6ip5	NM_022992	7	641	AAAGUGC	647	3 UTR	0.0443
Adcy2	NM_153534	7	3584	AAAGUGC	3590	3 UTR	0.0405
Orm2	NM_011016	7	722	AAAGUGC	728	3 UTR	0.0068
Nup35	NM_027091	7	1210	AAAGUGC	1216	3 UTR	0.0303
Dnajc2	NM_009584	7	2003	AAAGUGC	2009	3 UTR	0.0035
Mcm3	NM_008563	7	2728	AAAGUGC	2734	3 UTR	0.0266
OTTMUSG00000005491	NM_001013783	7	2155	AAAGUGC	2161	3 UTR	0.0069
2210018M11Rik	NM_172280	7	3948	AAAGUGC	3954	3 UTR	0.0061
Mat2b	NM_134017	7	1122	AAAGUGC	1128	3 UTR	0.0432
9530002B09Rik	NM_023865	7	812	AAAGUGC	818	3 UTR	0.0187
0610010E21Rik	NM_001033140	7	625	AAAGUGC	631	3 UTR	0.0350
Mrpl3	NM_053159	7	1232	AAAGUGC	1238	3 UTR	0.0200
Serpinb9b	NM_011452	7	1435	AAAGUGC	1441	3 UTR	0.0359
Rasd1	NM_009026	7	1497	AAAGUGC	1503	3 UTR	0.0377
Kcnq2	NM_001003824	7	2598	AAAGUGC	2604	3 UTR	0.0356
Ninj2	NM_016718	7	656	AAAGUGC	662	3 UTR	0.0208
Fbxo39	NM_001099688	7	1534	AAAGUGC	1540	3 UTR	0.0126
Dsg4	NM_181564	7	3417	AAAGUGC	3423	3 UTR	0.0132
Trim3	NM_018880	7	2525	AAAGUGC	2531	3 UTR	0.0353
Gabrg2	NM_008073	7	1831	AAAGUGC	1837	3 UTR	0.0276
Neurog1	NM_010896	7	1222	AAAGUGC	1228	3 UTR	0.0442
Laptm4a	NM_008640	7	1872	AAAGUGC	1878	3 UTR	0.0331
Hacl1	NM_019975	7	2218	AAAGUGC	2224	3 UTR	0.0468
Cyp46a1	NM_010010	7	2041	AAAGUGC	2047	3 UTR	0.0347
Ttc30b	NM_028235	7	2190	AAAGUGC	2196	3 UTR	0.0350
Pthlh	NM_008970	7	1333	AAAGUGC	1339	3 UTR	0.0276
Nt5dc2	NM_027289	7	1331	AAAGUGC	1337	3 UTR	0.0065
Sorl1	NM_011436	7	6902	AAAGUGC	6908	3 UTR	0.0069
Men1	NM_008583	7	2641	AAAGUGC	2647	3 UTR	0.0434
Serpinb9c	NM_011453	7	1292	AAAGUGC	1298	3 UTR	0.0419
Pdcd1lg2	NM_021396	7	1090	AAAGUGC	1096	3 UTR	0.0419

Tmem63a	NM_144794	7	2743	AAAGUGC	2749	3 UTR	0.0458
Pla2g6	NM_016915	7	2499	AAAGUGC	2505	3 UTR	0.0314
Pus1	NM_001025561	7	1809	AAAGUGC	1815	3 UTR	0.0177
BC064033	NM_173375	7	822	AAAGUGC	828	3 UTR	0.0464
Zfp553	NM_146201	7	2437	AAAGUGC	2443	3 UTR	0.0493
Clpx	NM_011802	7	2835	AAAGUGC	2841	3 UTR	0.0450
Orm3	NM_013623	7	720	AAAGUGC	726	3 UTR	0.0069
Dusp8	NM_008748	7	2418	AAAGUGC	2424	3 UTR	0.0220
Nkx2-5	NM_008700	7	1374	AAAGUGC	1380	3 UTR	0.0216
Nagk	NM_019542	7	1324	AAAGUGC	1330	3 UTR	0.0072
Bcap31	NM_012060	7	1070	AAAGUGC	1076	3 UTR	0.0226
Thra	NM_178060	7	2197	AAAGUGC	2203	3 UTR	0.0350
Gbf1	NM_178930	7	6188	AAAGUGC	6194	3 UTR	0.0354
5430432M24Rik	NM_146127	7	1243	AAAGUGC	1249	3 UTR	0.0345
Usp3	NM_144937	7	1755	AAAGUGC	1761	3 UTR	0.0319
3200002M19Rik	NM_027532	7	619	AAAGUGC	625	3 UTR	0.0183
Nrbp1	NM_147201	7	1886	AAAGUGC	1892	3 UTR	0.0261
Xpc	NM_009531	7	3400	AAAGUGC	3406	3 UTR	0.0449
Wbp7	NM_029274	7	8384	AAAGUGC	8390	3 UTR	0.0185
Pde5a	NM_153422	7	2748	AAAGUGC	2754	3 UTR	0.0488
Ppapdc1b	NM_028000	7	963	AAAGUGC	969	3 UTR	0.0395
Bmpr2	NM_007561	7	3334	AAAGUGC	3340	3 UTR	0.0226
Egr3	NM_018781	7	1365	AAAGUGC	1371	3 UTR	0.0153
Prl7d1	NM_011120	7	846	AAAGUGC	852	3 UTR	0.0103
2610020H08Rik	NM_001004187	7	1662	AAAGUGC	1668	3 UTR	0.0115
Lilrb3	NM_011095	7	2650	AAAGUGC	2656	3 UTR	0.0089
AC124479.4-201	NM_001081471	7	1212	AAAGUGC	1218	3 UTR	0.0424
Mrps24	NM_026080	7	547	AAAGUGC	553	3 UTR	0.0280
Cldn5	NM_013805	7	998	AAAGUGC	1004	3 UTR	0.0364
Phf1	NM_009343	7	2141	AAAGUGC	2147	3 UTR	0.0196
AC154553.3	NM_018751	7	1458	AAAGUGC	1464	3 UTR	0.0306
Fastk	NM_023229	7	1783	AAAGUGC	1789	3 UTR	0.0069
C430048L16Rik	NM_198957	7	3233	AAAGUGC	3239	3 UTR	0.0392
Ifit3	NM_010501	7	1381	AAAGUGC	1387	3 UTR	0.0427
Trem2	NM_031254	7	1064	AAAGUGC	1070	3 UTR	0.0169
Btbd7	NM_172806	7	4072	AAAGUGC	4078	3 UTR	0.0290
Tcea2	NM_009326	7	1117	AAAGUGC	1123	3 UTR	0.0101
Lipt1	NM_001037918	7	1428	AAAGUGC	1434	3 UTR	0.0189
Rft1	NM_177815	7	1681	AAAGUGC	1687	3 UTR	0.0430
Fanci	NM_145946	7	4586	AAAGUGC	4592	3 UTR	0.0333
Apol10b	NM_177820	7	1737	AAAGUGC	1743	3 UTR	0.0485
Xrcc4	NM_028012	7	1339	AAAGUGC	1345	3 UTR	0.0263
2410004L22Rik	NM_029621	7	1341	AAAGUGC	1347	3 UTR	0.0163
C8b	NM_133882	7	2067	AAAGUGC	2073	3 UTR	0.0234
Plekhg5	NM_001004156	7	3806	AAAGUGC	3812	3 UTR	0.0493
5730410E15Rik	NM_176998	7	2830	AAAGUGC	2836	3 UTR	0.0428
Mcm7	NM_008568	7	2348	AAAGUGC	2354	3 UTR	0.0079
Ctxn1	NM_183315	7	1011	AAAGUGC	1017	3 UTR	0.0448
Btg3	NM_009770	7	1111	AAAGUGC	1117	3 UTR	0.0264
Abcg2	NM_011920	7	2475	AAAGUGC	2481	3 UTR	0.0038
Sqstm1	NM_011018	7	1989	AAAGUGC	1995	3 UTR	0.0382
Myh14	NM_028021	7	6043	AAAGUGC	6049	3 UTR	0.0236
Tsg101	NM_021884	7	1415	AAAGUGC	1421	3 UTR	0.0353
Unc45a	NM_133952	7	3028	AAAGUGC	3034	3 UTR	0.0205
Prickle3	NM_175097	7	2369	AAAGUGC	2375	3 UTR	0.0274
Prom2	NM_138750	7	2811	AAAGUGC	2817	3 UTR	0.0105
Pdzd7	NM_177605	7	1626	AAAGUGC	1632	3 UTR	0.0424
Tnks1bp1	NM_001081260	7	5435	AAAGUGC	5441	3 UTR	0.0299
Hsd17b11	NM_053262	7	1352	AAAGUGC	1358	3 UTR	0.0445
Sds	NM_145565	7	1144	AAAGUGC	1150	3 UTR	0.0077
Cpne7	NM_170684	7	2285	AAAGUGC	2291	3 UTR	0.0374
Zfp54	NM_011760	7	2360	AAAGUGC	2366	3 UTR	0.0230
Topors	NM_134097	7	3448	AAAGUGC	3454	3 UTR	0.0345

Neurog3	NM_009719	7	1392	AAAGUGC	1398	3 UTR	0.0392
Spp1	NM_009263	7	1175	AAAGUGC	1181	3 UTR	0.0260
Smad6	NM_008542	7	2018	AAAGUGC	2024	3 UTR	0.0255
Cpe	NM_013494	7	1904	AAAGUGC	1910	3 UTR	0.0348
Gstm6	NM_008184	7	1043	AAAGUGC	1049	3 UTR	0.0229
Crct1	NM_028798	7	524	AAAGUGC	530	3 UTR	0.0189
Wasf1	NM_031877	7	2107	AAAGUGC	2113	3 UTR	0.0493
Prr15	NM_030024	7	1230	AAAGUGC	1236	3 UTR	0.0344
Cd6	NM_009852	7	2456	AAAGUGC	2462	3 UTR	0.0496
1700113O17Rik	NM_026627	7	442	AAAGUGC	448	3 UTR	0.0071
Xrn1	NM_011916	7	5408	AAAGUGC	5414	3 UTR	0.0142
Dyrk4	NM_207210	7	2055	AAAGUGC	2061	3 UTR	0.0100
Caml	NM_007596	7	1308	AAAGUGC	1314	3 UTR	0.0251
Nr2e3	NM_013708	7	1947	AAAGUGC	1953	3 UTR	0.0369
AC140392.3	NM_001039147	7	1576	AAAGUGC	1582	3 UTR	0.0390
AI314976	NM_207219	7	1016	AAAGUGC	1022	3 UTR	0.0289
Ccdc64b	NM_153784	7	1689	AAAGUGC	1695	3 UTR	0.0223
Ugdh	NM_009466	7	2017	AAAGUGC	2023	3 UTR	0.0449
Ccdc128	NM_028658	7	2599	AAAGUGC	2605	3 UTR	0.0390
Rnf151	NM_026205	7	1107	AAAGUGC	1113	3 UTR	0.0239

Comprehensive view of miRNA predicted targets on mRNA 3' UTR region produced by miRWalk and other program

Note : The below table displays all putative targets produced by both miRWalk and other programs i.e. contains all the putative targets of other programs (3rd party algorithms).

MicroRNA	Gene	miRanda	miRDB	miRWalk	RNA22	Targetscan	SUM
**mmu-miR-294**	**Nos2**	**1**	**0**	**1**	**0**	**1**	**3**

S4 Table: In silico analysis of predicted miR-721 mRNA targets. Prediction analysis of miR-721 binding sequences in the 3' UTR of mRNA targets using the miRWalk software.

Gene Name	RefSeqID	Seed Length	Start	Sequence	End	Pvalue
Nfya	NM_010913	14	3402	CAGUGCAAUUAAAA	3415	0.0000
Slc35d1	NM_177732	14	1565	CAGUGCAAUUAAAA	1578	0.0000
Ube2d1	NM_145420	12	1245	CAGUGCAAUUAA	1256	0.0001
Ott	NM_011022	11	1515	CAGUGCAAUUA	1525	0.0001
Zfand1	NM_025512	11	1451	CAGUGCAAUUA	1461	0.0002
Ttc39b	NM_027238	11	6669	CAGUGCAAUUA	6679	0.0016
OTTMUSG00000019138	NM_001037716	11	1436	CAGUGCAAUUA	1446	0.0001
Ebi2	NM_183031	11	1501	CAGUGCAAUUA	1511	0.0004
Sub1	NM_011294	10	1676	CAGUGCAAUU	1685	0.0027
Slc24a2	NM_172426	10	6161	CAGUGCAAUU	6170	0.0078
Erlin2	NM_153592	10	2135	CAGUGCAAUU	2144	0.0027
Uts2d	NM_198166	10	645	CAGUGCAAUU	654	0.0004
Cyld	NM_173369	10	5159	CAGUGCAAUU	5168	0.0047
Gjd2	NM_010290	10	2127	CAGUGCAAUU	2136	0.0014
Manba	NM_027288	10	3604	CAGUGCAAUU	3613	0.0009
Tmem178	NM_026516	10	1200	CAGUGCAAUU	1209	0.0007
Rasef	NM_001017427	10	2213	CAGUGCAAUU	2222	0.0029
Ppp1r9a	NM_181595	10	8589	CAGUGCAAUU	8598	0.0056
AC121108.12	NM_030732	10	4395	CAGUGCAAUU	4404	0.0059
Tspyl2	NM_029836	10	2554	CAGUGCAAUU	2563	0.0006
Acyp2	NM_029344	10	354	CAGUGCAAUU	363	0.0003
Gadd45a	NM_007836	10	690	CAGUGCAAUU	699	0.0005
Frmd8	NM_026169	10	2240	CAGUGCAAUU	2249	0.0016
Clock	NM_007715	10	3811	CAGUGCAAUU	3820	0.0043
Zfp110	NM_022981	10	3125	CAGUGCAAUU	3134	0.0006
Cerk	NM_145475	10	3841	CAGUGCAAUU	3850	0.0023
Rdh16	NM_009040	10	3124	CAGUGCAAUU	3133	0.0021
Phf3	NM_001081080	10	6475	CAGUGCAAUU	6484	0.0014
Tnfrsf1b	NM_011610	10	2823	CAGUGCAAUU	2832	0.0033
Rxfp3	NM_178717	10	3533	CAGUGCAAUU	3542	0.0020
C030011O14Rik	NM_174868	10	3803	CAGUGCAAUU	3812	0.0027
EG240055	NM_001081656	10	4637	CAGUGCAAUU	4646	0.0043
Slc39a3	NM_134135	9	2846	CAGUGCAAU	2854	0.0092
Fut9	NM_010243	9	2843	CAGUGCAAU	2851	0.0389
Aff1	NM_133919	9	5883	CAGUGCAAU	5891	0.0170
Phactr2	NM_001033257	9	1815	CAGUGCAAU	1823	0.0033
Hepacam2	NM_178899	9	2125	CAGUGCAAU	2133	0.0031
Cdc2l6	NM_198164	9	2793	CAGUGCAAU	2801	0.0089
Tmprss3	NM_080727	9	1530	CAGUGCAAU	1538	0.0051
Atp9b	NM_015805	9	3615	CAGUGCAAU	3623	0.0036
Kcna4	NM_021275	9	3250	CAGUGCAAU	3258	0.0061
Plekha2	NM_031257	9	2488	CAGUGCAAU	2496	0.0070
9930013L23Rik	NM_030728	9	4808	CAGUGCAAU	4816	0.0032
Pfkfb4	NM_173019	9	3296	CAGUGCAAU	3304	0.0070
Usp48	NM_130879	9	3526	CAGUGCAAU	3534	0.0092
Ano5	NM_177694	9	5716	CAGUGCAAU	5724	0.0184
Mcam	NM_023061	9	1989	CAGUGCAAU	1997	0.0035
Ralbp1	NM_009067	9	3163	CAGUGCAAU	3171	0.0059
Erc1	NM_053204	9	6244	CAGUGCAAU	6252	0.0183
Inoc1	NM_026574	9	5802	CAGUGCAAU	5810	0.0050
Tbc1d1	NM_019636	9	1292	CAGUGCAAU	1300	0.0205
Bbx	NM_027444	9	7000	CAGUGCAAU	7008	0.0216
Itga4	NM_010576	9	5329	CAGUGCAAU	5337	0.0245
Fbxw5	NM_013908	9	1915	CAGUGCAAU	1923	0.0019
Btbd9	NM_172618	9	2279	CAGUGCAAU	2287	0.0176
Csf1	NM_007778	9	2851	CAGUGCAAU	2859	0.0081

Tnpo1	NM_178716	9	4789	CAGUGCAAU	4797	0.0098
Stk38l	NM_172734	9	1772	CAGUGCAAU	1780	0.0118
Cyp4a14	NM_007822	9	1828	CAGUGCAAU	1836	0.0036
Rybp	NM_019743	9	2931	CAGUGCAAU	2939	0.0137
Kdelc2	NM_212445	9	2598	CAGUGCAAU	2606	0.0078
Eefsec	NM_023060	9	1914	CAGUGCAAU	1922	0.0033
Pramef12	NM_029948	9	2297	CAGUGCAAU	2305	0.0035
Jub	NM_010590	9	2886	CAGUGCAAU	2894	0.0056
Zfp780b	NM_001081021	9	3770	CAGUGCAAU	3778	0.0110
Kcne2	NM_134110	9	1492	CAGUGCAAU	1500	0.0045
Mtmr12	NM_172958	9	3562	CAGUGCAAU	3570	0.0082
Hs3st1	NM_010474	9	1519	CAGUGCAAU	1527	0.0016
Dpy19l1	NM_172920	9	319	CAGUGCAAU	327	0.0180
Mll1	NM_001081049	9	14253	CAGUGCAAU	14261	0.0172
Sgms1	NM_144792	9	2062	CAGUGCAAU	2070	0.0063
Mtmr1	NM_016985	9	3263	CAGUGCAAU	3271	0.0094
Ensa	NM_001026212	9	1892	CAGUGCAAU	1900	0.0068
Cflar	NM_207653	9	2971	CAGUGCAAU	2979	0.0188
Robo1	NM_019413	9	6175	CAGUGCAAU	6183	0.0066
Lonrf2	NM_001029878	9	3241	CAGUGCAAU	3249	0.0133
Hspa4	NM_008300	9	3764	CAGUGCAAU	3772	0.0069
Flvcr2	NM_145447	9	2737	CAGUGCAAU	2745	0.0056
Gpr107	NM_178760	9	3738	CAGUGCAAU	3746	0.0085
Isx	NM_027837	9	1698	CAGUGCAAU	1706	0.0023
Zfp784	NM_001039532	9	1345	CAGUGCAAU	1353	0.0046
Usp8	NM_019729	9	3867	CAGUGCAAU	3875	0.0027
5430435G22Rik	NM_145509	9	1326	CAGUGCAAU	1334	0.0068
Zfp653	NM_177318	9	2055	CAGUGCAAU	2063	0.0012
Ms4a4b	NM_021718	9	855	CAGUGCAAU	863	0.0018
Phf20	NM_172674	9	5596	CAGUGCAAU	5604	0.0096
Mbip	NM_145442	9	1239	CAGUGCAAU	1247	0.0015
Cox5b	NM_009942	9	549	CAGUGCAAU	557	0.0004
Cmtm6	NM_026036	9	1183	CAGUGCAAU	1191	0.0105
Trib2	NM_144551	9	2677	CAGUGCAAU	2685	0.0071
Btbd3	NM_145534	9	4684	CAGUGCAAU	4692	0.0113
C1galt1	NM_052993	9	1194	CAGUGCAAU	1202	0.0031
Tmem109	NM_134142	9	1272	CAGUGCAAU	1280	0.0042
Mesdc2	NM_023403	9	3321	CAGUGCAAU	3329	0.0134
5730455P16Rik	NM_027472	9	1280	CAGUGCAAU	1288	0.0099
Tiam2	NM_011878	8	6118	CAGUGCAA	6125	0.0102
Ugt8a	NM_011674	8	2908	CAGUGCAA	2915	0.0282
Cxcl13	NM_018866	8	1025	CAGUGCAA	1032	0.0121
Sgpp2	NM_001004173	8	3523	CAGUGCAA	3530	0.0427
Acvr1	NM_007394	8	2405	CAGUGCAA	2412	0.0169
Numa1	NM_133947	8	7049	CAGUGCAA	7056	0.0117
Armc9	NM_027507	8	3673	CAGUGCAA	3680	0.0396
D4Ertd22e	NM_001025608	8	628	CAGUGCAA	635	0.0413
Ube4b	NM_022022	8	4652	CAGUGCAA	4659	0.0194
Gemin8	NM_146238	8	1374	CAGUGCAA	1381	0.0153
Scamp5	NM_020270	8	1062	CAGUGCAA	1069	0.0368
Slc22a2	NM_013667	8	1911	CAGUGCAA	1918	0.0058
Gnpnat1	NM_019425	8	2152	CAGUGCAA	2159	0.0288
Cdc2a	NM_007659	8	2578	CAGUGCAA	2585	0.0280
Nr2c2ap	NM_001025586	8	895	CAGUGCAA	902	0.0081
Zfp560	NM_001004190	8	4523	CAGUGCAA	4530	0.0325
1110018G07Rik	NM_178065	8	3907	CAGUGCAA	3914	0.0355
Tomm7	NM_025394	8	871	CAGUGCAA	878	0.0134
Ttyh3	NM_175274	8	2567	CAGUGCAA	2574	0.0421
Tmem9b	NM_020050	8	1211	CAGUGCAA	1218	0.0151

Zfpm2	NM_011766	8	4277	CAGUGCAA	4284	0.0154
Rab14	NM_026697	8	1965	CAGUGCAA	1972	0.0313
Taf4a	NM_001081092	8	3798	CAGUGCAA	3805	0.0195
B4galt5	NM_019835	8	4061	CAGUGCAA	4068	0.0432
Txnip	NM_001009935	8	2094	CAGUGCAA	2101	0.0201
D130043K22Rik	NM_001081051	8	3626	CAGUGCAA	3633	0.0222
2600010E01Rik	NM_001083810	8	2338	CAGUGCAA	2345	0.0415
Stam	NM_011484	8	1984	CAGUGCAA	1991	0.0284
Pank1	NM_023792	8	1790	CAGUGCAA	1797	0.0219
Jph3	NM_020605	8	3201	CAGUGCAA	3208	0.0203
Mfge8	NM_008594	8	1978	CAGUGCAA	1985	0.0100
Adcy2	NM_153534	8	3724	CAGUGCAA	3731	0.0103
Fbln7	NM_024237	8	2715	CAGUGCAA	2722	0.0220
1300001I01Rik	NM_001081158	8	4348	CAGUGCAA	4355	0.0187
Mafb	NM_010658	8	2894	CAGUGCAA	2901	0.0295
9130213B05Rik	NM_145562	8	1358	CAGUGCAA	1365	0.0151
Nrarp	NM_025980	8	1371	CAGUGCAA	1378	0.0285
Tbc1d9b	NM_029745	8	4286	CAGUGCAA	4293	0.0212
Lin28	NM_145833	8	811	CAGUGCAA	818	0.0415
Slitrk5	NM_198865	8	4239	CAGUGCAA	4246	0.0202
Dnajb6	NM_001037940	8	2641	CAGUGCAA	2648	0.0206
Fmo9	NM_172844	8	2165	CAGUGCAA	2172	0.0186
Pde1a	NM_016744	8	2128	CAGUGCAA	2135	0.0374
Mknk1	NM_021461	8	2118	CAGUGCAA	2125	0.0161
E2f4	NM_148952	8	1466	CAGUGCAA	1473	0.0108
Nf1	NM_010897	8	8697	CAGUGCAA	8704	0.0478
Lad1	NM_133664	8	2485	CAGUGCAA	2492	0.0155
E2f7	NM_178609	8	5262	CAGUGCAA	5269	0.0381
Arfip1	NM_001081093	8	1649	CAGUGCAA	1656	0.0252
BC003266	NM_030252	8	779	CAGUGCAA	786	0.0097
Wscd2	NM_177292	8	3104	CAGUGCAA	3111	0.0456
Dennd3	NM_001081066	8	4447	CAGUGCAA	4454	0.0195
Galnact2	NM_030165	8	2792	CAGUGCAA	2799	0.0251
Ntrk2	NM_001025074	8	3890	CAGUGCAA	3897	0.0245
Hectd2	NM_172637	8	3986	CAGUGCAA	3993	0.0343
Zmat4	NM_177086	8	3693	CAGUGCAA	3700	0.0497
Myst2	NM_177619	8	2609	CAGUGCAA	2616	0.0232
Tmem16a	NM_178642	8	4224	CAGUGCAA	4231	0.0206
Zfp39	NM_011758	8	3766	CAGUGCAA	3773	0.0245
Tspan1	NM_133681	8	1291	CAGUGCAA	1298	0.0167
Lrch1	NM_001033439	8	3396	CAGUGCAA	3403	0.0354
Nptn	NM_009145	8	1764	CAGUGCAA	1771	0.0157
Barhl2	NM_001005477	8	2046	CAGUGCAA	2053	0.0129
Fmr1	NM_008031	8	2862	CAGUGCAA	2869	0.0346
Sgcb	NM_011890	8	1176	CAGUGCAA	1183	0.0410
Epb4.9	NM_013514	8	2626	CAGUGCAA	2633	0.0162
Rdh13	NM_175372	8	1248	CAGUGCAA	1255	0.0293
Nrf1	NM_010938	8	3015	CAGUGCAA	3022	0.0268
Sephs2	NM_009266	8	1761	CAGUGCAA	1768	0.0107
Adamts18	NM_172466	8	4246	CAGUGCAA	4253	0.0126
Nup62	NM_053074	8	2142	CAGUGCAA	2149	0.0103
Syne1	NM_153399	8	6379	CAGUGCAA	6386	0.0338
Trim45	NM_194343	8	3447	CAGUGCAA	3454	0.0367
Madd	NM_145527	8	5426	CAGUGCAA	5433	0.0161
Map2k1	NM_008927	8	1791	CAGUGCAA	1798	0.0135
Fbxo3	NM_212433	8	4246	CAGUGCAA	4253	0.0470
Mcart6	NM_001082412	8	2685	CAGUGCAA	2692	0.0237
Agrn	NM_021604	8	5896	CAGUGCAA	5903	0.0186
Csnk2a1	NM_007788	8	3542	CAGUGCAA	3549	0.0414

Mfsd2	NM_029662	8	1821	CAGUGCAA	1828	0.0062
Hivep1	NM_007772	8	8565	CAGUGCAA	8572	0.0078
Stard13	NM_146258	8	4236	CAGUGCAA	4243	0.0307
Pmm2	NM_016881	8	1081	CAGUGCAA	1088	0.0152
Enpp4	NM_199016	8	4258	CAGUGCAA	4265	0.0448
BC006779	NM_183162	8	9433	CAGUGCAA	9440	0.0173
Saps1	NM_172894	8	3216	CAGUGCAA	3223	0.0116
Clcn4-2	NM_011334	8	2802	CAGUGCAA	2809	0.0287
Smad2	NM_010754	8	2255	CAGUGCAA	2262	0.0152
A130022J15Rik	NM_175313	8	2392	CAGUGCAA	2399	0.0169
Snx7	NM_029655	8	1337	CAGUGCAA	1344	0.0069
Canx	NM_007597	8	3524	CAGUGCAA	3531	0.0349
Adamts1	NM_009621	8	3616	CAGUGCAA	3623	0.0240
Bcap29	NM_007530	8	1675	CAGUGCAA	1682	0.0152
Sulf1	NM_172294	8	3220	CAGUGCAA	3227	0.0233
4931406C07Rik	NM_133732	8	1616	CAGUGCAA	1623	0.0223
Mier1	NM_027696	8	4686	CAGUGCAA	4693	0.0447
Pcdhb18	NM_053143	8	4968	CAGUGCAA	4975	0.0375
1110054O05Rik	NM_001013577	8	714	CAGUGCAA	721	0.0407
2310046K01Rik	NM_027172	8	1692	CAGUGCAA	1699	0.0166
Rpo1-1	NM_009085	8	1168	CAGUGCAA	1175	0.0028
Hrb	NM_010472	8	1982	CAGUGCAA	1989	0.0184
1110033F04Rik	NM_001048196	8	767	CAGUGCAA	774	0.0068
Chmp7	NM_134078	8	2459	CAGUGCAA	2466	0.0191
Gpr12	NM_001010941	8	1595	CAGUGCAA	1602	0.0147
Nek6	NM_021606	8	3458	CAGUGCAA	3465	0.0396
Irf4	NM_013674	8	3542	CAGUGCAA	3549	0.0485
Actr5	NM_175419	8	2327	CAGUGCAA	2334	0.0049
Sepp1	NM_009155	8	1749	CAGUGCAA	1756	0.0121
Etl4	NM_001081006	8	6853	CAGUGCAA	6860	0.0200
Usp33	NM_133247	8	3442	CAGUGCAA	3449	0.0191
Agtr1a	NM_177322	8	2059	CAGUGCAA	2066	0.0126
Lnpep	NM_172827	8	3236	CAGUGCAA	3243	0.0304
Cst6	NM_028623	8	795	CAGUGCAA	802	0.0485
Stk11ip	NM_027886	8	3394	CAGUGCAA	3401	0.0169
Ssbp2	NM_024272	8	2357	CAGUGCAA	2364	0.0409
Tnip3	NM_001001495	8	3587	CAGUGCAA	3594	0.0438
D1Ertd622e	NM_133825	8	1415	CAGUGCAA	1422	0.0275
Slc17a1	NM_009198	8	1865	CAGUGCAA	1872	0.0069
Prei4	NM_028802	8	2644	CAGUGCAA	2651	0.0222
Mdfic	NM_175088	8	1235	CAGUGCAA	1242	0.0345
Ube3b	NM_054093	8	4407	CAGUGCAA	4414	0.0226
Arrdc3	NM_001042591	8	2389	CAGUGCAA	2396	0.0393
Dynll2	NM_026556	8	607	CAGUGCAA	614	0.0286
Ptpre	NM_011212	8	3478	CAGUGCAA	3485	0.0446
Limk2	NM_010718	8	3494	CAGUGCAA	3501	0.0353
Ddx58	NM_172689	8	3390	CAGUGCAA	3397	0.0305
Csmd1	NM_053171	8	12681	CAGUGCAA	12688	0.0472
Fut11	NM_028428	8	2113	CAGUGCAA	2120	0.0277
S1pr3	NM_010101	8	1997	CAGUGCAA	2004	0.0429
Skp1a	NM_011543	8	666	CAGUGCAA	673	0.0127
Mgst3	NM_025569	8	573	CAGUGCAA	580	0.0018
Gap43	NM_008083	8	1106	CAGUGCAA	1113	0.0073
Limd2	NM_172397	8	2599	CAGUGCAA	2606	0.0362
Snx27	NM_001082484	8	2113	CAGUGCAA	2120	0.0083
2310035C23Rik	NM_173187	8	4032	CAGUGCAA	4039	0.0230
Ptgfr	NM_008966	8	1725	CAGUGCAA	1732	0.0449
Baz2a	NM_054078	8	7356	CAGUGCAA	7363	0.0382
5730470L24Rik	NM_025679	8	1857	CAGUGCAA	1864	0.0316

Il6st	NM_010560	8	3220	CAGUGCAA	3227	0.0367
C330016O10Rik	NM_145974	8	2107	CAGUGCAA	2114	0.0084
Amigo1	NM_001004293	8	3822	CAGUGCAA	3829	0.0467
Akap1	NM_009648	8	2788	CAGUGCAA	2795	0.0146
Fbxw8	NM_172721	8	2178	CAGUGCAA	2185	0.0463
Ccna2	NM_009828	8	2231	CAGUGCAA	2238	0.0209
Nox4	NM_015760	8	2728	CAGUGCAA	2735	0.0258
Gbl	NM_019988	8	1814	CAGUGCAA	1821	0.0342
Homer3	NM_011984	8	1261	CAGUGCAA	1268	0.0182
Cd1d1	NM_007639	8	1353	CAGUGCAA	1360	0.0113
Padi6	NM_153106	8	2192	CAGUGCAA	2199	0.0034
Tcp1	NM_013686	8	1908	CAGUGCAA	1915	0.0090
Add1	NM_001024458	8	3647	CAGUGCAA	3654	0.0225
D14Ertd436e	NM_172599	8	2968	CAGUGCAA	2975	0.0292
Nkd1	NM_001081364	8	6274	CAGUGCAA	6281	0.0121
Inhbb	NM_008381	8	3784	CAGUGCAA	3791	0.0279
Mpp3	NM_007863	8	2117	CAGUGCAA	2124	0.0138
Wnt2b	NM_009520	8	1722	CAGUGCAA	1729	0.0325
Lif	NM_008501	8	3800	CAGUGCAA	3807	0.0474
Il17rb	NM_019583	8	1946	CAGUGCAA	1953	0.0069
Ltf	NM_008522	8	2604	CAGUGCAA	2611	0.0089
Spsb3	NM_027141	8	1519	CAGUGCAA	1526	0.0068
Ear11	NM_053113	8	590	CAGUGCAA	597	0.0032
Mmd	NM_026178	8	1061	CAGUGCAA	1068	0.0275
Zfp352	NM_153102	8	2128	CAGUGCAA	2135	0.0064
Zfyve26	NM_001008550	8	9101	CAGUGCAA	9108	0.0244
Plat	NM_008872	8	2181	CAGUGCAA	2188	0.0115
Btaf1	NM_001080706	8	6028	CAGUGCAA	6035	0.0397
Ubxn8	NM_178648	8	1002	CAGUGCAA	1009	0.0292
Tmem55a	NM_028264	8	1997	CAGUGCAA	2004	0.0210
Arl6ip1	NM_019419	8	1216	CAGUGCAA	1223	0.0213
Hadhb	NM_145558	8	1494	CAGUGCAA	1501	0.0077
Map2k2	NM_023138	8	2170	CAGUGCAA	2177	0.0149
Ivns1abp	NM_001039512	8	3364	CAGUGCAA	3371	0.0163
Tor3a	NM_023141	8	1840	CAGUGCAA	1847	0.0173
Lipa	NM_021460	8	2448	CAGUGCAA	2455	0.0254
Pea15a	NM_011063	8	1543	CAGUGCAA	1550	0.0285
Depdc1b	NM_178683	8	2205	CAGUGCAA	2212	0.0130
Ube2d3	NM_025356	8	2295	CAGUGCAA	2302	0.0291
Nme4	NM_019731	8	584	CAGUGCAA	591	0.0043
Abca1	NM_013454	8	7159	CAGUGCAA	7166	0.0470
Usp32	NM_001029934	8	6112	CAGUGCAA	6119	0.0297
Wnt1	NM_021279	8	1683	CAGUGCAA	1690	0.0140
Mllt10	NM_010804	8	3461	CAGUGCAA	3468	0.0238
Kcns2	NM_181317	8	3999	CAGUGCAA	4006	0.0361
Apobec2	NM_009694	8	914	CAGUGCAA	921	0.0064
Zfp41	NM_001044718	8	2973	CAGUGCAA	2980	0.0396
S1pr1	NM_007901	8	1929	CAGUGCAA	1936	0.0205
Chrnb4	NM_148944	8	1705	CAGUGCAA	1712	0.0293
Snx22	NM_001025612	8	770	CAGUGCAA	777	0.0289
5031414D18Rik	NM_198642	8	2469	CAGUGCAA	2476	0.0069
Rph3a	NM_011286	8	4087	CAGUGCAA	4094	0.0253
Loh12cr1	NM_026371	8	1369	CAGUGCAA	1376	0.0136
Akap7	NM_018747	8	2837	CAGUGCAA	2844	0.0297
Tpp1	NM_009906	8	3358	CAGUGCAA	3365	0.0269
Tnrc6c	NM_198022	8	7084	CAGUGCAA	7091	0.0389
Efnb2	NM_010111	8	4012	CAGUGCAA	4019	0.0469
Meox2	NM_008584	8	1467	CAGUGCAA	1474	0.0174
Rtcd1	NM_025517	8	1250	CAGUGCAA	1257	0.0041

Eif4h	NM_033561	8	1636	CAGUGCAA	1643	0.0246
Npepl1	NM_213733	8	1685	CAGUGCAA	1692	0.0070
Ykt6	NM_019661	8	1108	CAGUGCAA	1115	0.0261
Mnt	NM_010813	8	3323	CAGUGCAA	3330	0.0363
Itpk1	NM_172584	8	2373	CAGUGCAA	2380	0.0233
Eif4e3	NM_025829	8	1970	CAGUGCAA	1977	0.0281
Med17	NM_144933	8	3222	CAGUGCAA	3229	0.0284
Snf1lk	NM_010831	8	2692	CAGUGCAA	2699	0.0306
Upk1b	NM_178924	8	1364	CAGUGCAA	1371	0.0138
B3galnt1	NM_020026	8	1864	CAGUGCAA	1871	0.0113
1810026J23Rik	NM_178619	8	3026	CAGUGCAA	3033	0.0319
Fggy	NM_029347	8	1609	CAGUGCAA	1616	0.0091
Rad23b	NM_009011	8	3000	CAGUGCAA	3007	0.0338
Clrn3	NM_178669	8	852	CAGUGCAA	859	0.0372
Lrp2	NM_001081088	8	14360	CAGUGCAA	14367	0.0198
Prnp	NM_011170	8	1878	CAGUGCAA	1885	0.0190
Pygb	NM_153781	8	3580	CAGUGCAA	3587	0.0190
Ppp1r2	NM_025800	8	1571	CAGUGCAA	1578	0.0472
Rab34	NM_033475	8	1364	CAGUGCAA	1371	0.0055
Rnpepl1	NM_181405	8	1933	CAGUGCAA	1940	0.0115
Tmem63b	NM_198167	8	3095	CAGUGCAA	3102	0.0094
Fzd4	NM_008055	8	2677	CAGUGCAA	2684	0.0263
Acot8	NM_133240	8	1065	CAGUGCAA	1072	0.0024
Leng1	NM_027203	8	1714	CAGUGCAA	1721	0.0157
AC123686.11-201	NM_029048	8	1764	CAGUGCAA	1771	0.0274
Ucp3	NM_009464	8	1281	CAGUGCAA	1288	0.0200
Wasl	NM_028459	8	3988	CAGUGCAA	3995	0.0373
Cblb	NM_001033238	8	5985	CAGUGCAA	5992	0.0499
Tulp3	NM_011657	8	2032	CAGUGCAA	2039	0.0292
Pip4k2a	NM_008845	8	2144	CAGUGCAA	2151	0.0319
Akap4	NM_009651	8	2794	CAGUGCAA	2801	0.0029
Gpd1	NM_010271	8	2331	CAGUGCAA	2338	0.0263
Slc25a19	NM_026071	8	1252	CAGUGCAA	1259	0.0065
Papss2	NM_011864	8	2256	CAGUGCAA	2263	0.0244
Nat14	NM_201355	7	1332	CAGUGCA	1338	0.0333
Dpm2	NM_010073	7	400	CAGUGCA	406	0.0342
Apol7c	NM_175391	7	1561	CAGUGCA	1567	0.0458
Oas1c	NM_033541	7	2137	CAGUGCA	2143	0.0497
Chrne	NM_009603	7	1505	CAGUGCA	1511	0.0060
Tmsb4x	NM_021278	7	284	CAGUGCA	290	0.0245
Gadd45g	NM_011817	7	969	CAGUGCA	975	0.0307
1810023F06Rik	NM_145449	7	995	CAGUGCA	1001	0.0142
Dpp3	NM_133803	7	2426	CAGUGCA	2432	0.0254
Tex24	NM_001013609	7	1390	CAGUGCA	1396	0.0414
Hspa1b	NM_010478	7	2399	CAGUGCA	2405	0.0389
4930589M24Rik	NM_001081428	7	3591	CAGUGCA	3597	0.0294
Thsd1	NM_019576	7	2673	CAGUGCA	2679	0.0133
1810037C20Rik	NM_025473	7	1541	CAGUGCA	1547	0.0371
Acoxl	NM_028765	7	2606	CAGUGCA	2612	0.0440
Ccnf	NM_007634	7	2985	CAGUGCA	2991	0.0398
Kcnj8	NM_008428	7	1695	CAGUGCA	1701	0.0398
Nola2	NM_026631	7	816	CAGUGCA	822	0.0299
Txndc12	NM_025334	7	1093	CAGUGCA	1099	0.0403
Slc2a6	NM_172659	7	1633	CAGUGCA	1639	0.0330
Gimap7	NM_146167	7	1217	CAGUGCA	1223	0.0177
Rmnd1	NM_025343	7	1742	CAGUGCA	1748	0.0178
Ndfip1	NM_022996	7	830	CAGUGCA	836	0.0126
Poll	NM_020032	7	2086	CAGUGCA	2092	0.0319
0610012H03Rik	NM_028747	7	1175	CAGUGCA	1181	0.0469

Lancl2	NM_133737	7	2317	CAGUGCA	2323	0.0443
Aes	NM_010347	7	975	CAGUGCA	981	0.0401
Slc26a4	NM_011867	7	2613	CAGUGCA	2619	0.0315
Nog	NM_008711	7	1402	CAGUGCA	1408	0.0409
Tspo	NM_009775	7	786	CAGUGCA	792	0.0157
Il13	NM_008355	7	592	CAGUGCA	598	0.0443
9930012K11Rik	NM_001004155	7	1288	CAGUGCA	1294	0.0466
Gm266	NM_001033248	7	932	CAGUGCA	938	0.0334
Oas1d	NM_133893	7	1542	CAGUGCA	1548	0.0362
Rcc2	NM_173867	7	1723	CAGUGCA	1729	0.0164
Cdh7	NM_172853	7	2729	CAGUGCA	2735	0.0387
AI118078	NM_172923	7	2399	CAGUGCA	2405	0.0384
Aurkb	NM_011496	7	1765	CAGUGCA	1771	0.0360
Vit	NM_028813	7	2394	CAGUGCA	2400	0.0371
BC052328	NM_198301	7	1134	CAGUGCA	1140	0.0419
Ebag9	NM_019480	7	1028	CAGUGCA	1034	0.0387
Msr2	NM_030707	7	1618	CAGUGCA	1624	0.0248
Gm853	NM_001034872	7	1569	CAGUGCA	1575	0.0453
Lrrc50	NM_026648	7	2551	CAGUGCA	2557	0.0470
Gcnt7	NM_001039560	7	1959	CAGUGCA	1965	0.0448
Ddit3	NM_007837	7	751	CAGUGCA	757	0.0124
Sh3tc2	NM_172628	7	4179	CAGUGCA	4185	0.0390
Hspa5	NM_022310	7	2197	CAGUGCA	2203	0.0208
Apbb3	NM_146085	7	1930	CAGUGCA	1936	0.0178
Pdlim7	NM_026131	7	863	CAGUGCA	869	0.0240
Mrps9	NM_023514	7	1297	CAGUGCA	1303	0.0129
Casr	NM_013803	7	3871	CAGUGCA	3877	0.0468
Hnt	NM_172290	7	1522	CAGUGCA	1528	0.0342
Ninj2	NM_016718	7	738	CAGUGCA	744	0.0208
H2-Q7	NM_010394	7	1072	CAGUGCA	1078	0.0202
Lypd3	NM_133743	7	1233	CAGUGCA	1239	0.0328
2410008K03Rik	NM_028022	7	1418	CAGUGCA	1424	0.0307
Gabrg2	NM_008073	7	1962	CAGUGCA	1968	0.0276
Adam8	NM_007403	7	2821	CAGUGCA	2827	0.0322
Hsd3b5	NM_008295	7	1327	CAGUGCA	1333	0.0224
Lmbr1l	NM_029098	7	1768	CAGUGCA	1774	0.0312
Tnnt3	NM_011620	7	836	CAGUGCA	842	0.0105
Eepd1	NM_026189	7	2433	CAGUGCA	2439	0.0311
Brpf1	NM_030178	7	3966	CAGUGCA	3972	0.0331
Chchd4	NM_133928	7	586	CAGUGCA	592	0.0441
Cyp46a1	NM_010010	7	1818	CAGUGCA	1824	0.0347
Top3a	NM_009410	7	3339	CAGUGCA	3345	0.0279
Speer4f	NM_027609	7	1008	CAGUGCA	1014	0.0272
Tmco4	NM_029857	7	3123	CAGUGCA	3129	0.0386
Ppp2r2d	NM_026391	7	1983	CAGUGCA	1989	0.0356
Abcf2	NM_013853	7	2034	CAGUGCA	2040	0.0329
Ccdc46	NM_029606	7	3223	CAGUGCA	3229	0.0271
Lrrc14	NM_145471	7	2090	CAGUGCA	2096	0.0267
BC046331	NM_177673	7	3121	CAGUGCA	3127	0.0263
Wnt10b	NM_011718	7	1737	CAGUGCA	1743	0.0426
Parp1	NM_007415	7	3292	CAGUGCA	3298	0.0442
Abi3	NM_025659	7	1148	CAGUGCA	1154	0.0195
BC029169	NM_153782	7	2370	CAGUGCA	2376	0.0269
Dok7	NM_172708	7	2264	CAGUGCA	2270	0.0421
Mlxipl	NM_021455	7	2751	CAGUGCA	2757	0.0337
Hsp90b1	NM_011631	7	2604	CAGUGCA	2610	0.0158
Wnt10a	NM_009518	7	2255	CAGUGCA	2261	0.0343
Rbm43	NM_030243	7	1824	CAGUGCA	1830	0.0442
Gstm1	NM_010358	7	942	CAGUGCA	948	0.0260

Ercc4	NM_015769	7	3129	CAGUGCA	3135	0.0296
Sardh	NM_138665	7	3016	CAGUGCA	3022	0.0092
Oas1e	NM_145210	7	1520	CAGUGCA	1526	0.0377
Zfp112	NM_021307	7	3493	CAGUGCA	3499	0.0428
Cnga4	NM_001033317	7	2161	CAGUGCA	2167	0.0376
Phc3	NM_153421	7	3118	CAGUGCA	3124	0.0317
Lsg1	NM_178069	7	2088	CAGUGCA	2094	0.0232
Gabrr1	NM_008075	7	1657	CAGUGCA	1663	0.0441
Gpi1	NM_008155	7	1776	CAGUGCA	1782	0.0155
Acsm5	NM_178758	7	1956	CAGUGCA	1962	0.0345
Thra	NM_178060	7	1803	CAGUGCA	1809	0.0350
Dexi	NM_021428	7	1232	CAGUGCA	1238	0.0443
Dnajc9	NM_134081	7	1149	CAGUGCA	1155	0.0432
Gdpd2	NM_023608	7	2022	CAGUGCA	2028	0.0378
Rassf8	NM_027760	7	1470	CAGUGCA	1476	0.0239
Syngr2	NM_009304	7	1430	CAGUGCA	1436	0.0486
Plekhg1	NM_001033253	7	4426	CAGUGCA	4432	0.0059
Rnaseh1	NM_011275	7	964	CAGUGCA	970	0.0315
Itsn2	NM_011365	7	5696	CAGUGCA	5702	0.0457
2310008M10Rik	NM_025509	7	606	CAGUGCA	612	0.0344
Il18bp	NM_010531	7	1134	CAGUGCA	1140	0.0412
Lcor	NM_172154	7	4231	CAGUGCA	4237	0.0301
Gcdh	NM_008097	7	1934	CAGUGCA	1940	0.0367
Taldo1	NM_011528	7	1072	CAGUGCA	1078	0.0117
Cdkn2d	NM_009878	7	1006	CAGUGCA	1012	0.0306
4931429I11Rik	NM_001081121	7	2490	CAGUGCA	2496	0.0412
Bag3	NM_013863	7	1861	CAGUGCA	1867	0.0348
Zbp1	NM_021394	7	1953	CAGUGCA	1959	0.0363
AC124479.4-201	NM_001081471	7	1179	CAGUGCA	1185	0.0424
BC024997	NM_146004	7	1956	CAGUGCA	1962	0.0112
Commd4	NM_025417	7	662	CAGUGCA	668	0.0121
Speer4d	NM_025759	7	913	CAGUGCA	919	0.0214
Oas1h	NM_145228	7	1570	CAGUGCA	1576	0.0341
BC048651	NM_207258	7	1334	CAGUGCA	1340	0.0319
Nkx6-1	NM_144955	7	1769	CAGUGCA	1775	0.0141
Tmsb10	NM_001039392	7	466	CAGUGCA	472	0.0160
Col25a1	NM_198711	7	2737	CAGUGCA	2743	0.0433
Vps39	NM_147153	7	2862	CAGUGCA	2868	0.0298
Ccdc59	NM_025602	7	1070	CAGUGCA	1076	0.0201
Ikzf5	NM_026849	7	2426	CAGUGCA	2432	0.0363
Snx2	NM_026386	7	1818	CAGUGCA	1824	0.0264
Slc35f5	NM_028787	7	2158	CAGUGCA	2164	0.0472
Clstn3	NM_153508	7	3426	CAGUGCA	3432	0.0492
Fanci	NM_145946	7	4125	CAGUGCA	4131	0.0333
Myst1	NM_026370	7	1496	CAGUGCA	1502	0.0068
Nip7	NM_025391	7	1183	CAGUGCA	1189	0.0447
Tcf25	NM_001037878	7	2553	CAGUGCA	2559	0.0394
Hspa12b	NM_028306	7	2649	CAGUGCA	2655	0.0457
Ng23	NM_023893	7	468	CAGUGCA	474	0.0234
Dvl3	NM_007889	7	2569	CAGUGCA	2575	0.0401
Glrx5	NM_028419	7	647	CAGUGCA	653	0.0277
Hp	NM_017370	7	1193	CAGUGCA	1199	0.0106
Srebf1	NM_011480	7	3584	CAGUGCA	3590	0.0496
Nppc	NM_010933	7	553	CAGUGCA	559	0.0317
Inf2	NM_198411	7	4248	CAGUGCA	4254	0.0407
Il10rb	NM_008349	7	1186	CAGUGCA	1192	0.0309
B4galt2	NM_017377	7	1853	CAGUGCA	1859	0.0496
Pepd	NM_008820	7	1783	CAGUGCA	1789	0.0207
Tmem131	NM_018872	7	5937	CAGUGCA	5943	0.0446

Snapc2	NM_133968	7	1311	CAGUGCA	1317	0.0258
Prrxl1	NM_001001796	7	1298	CAGUGCA	1304	0.0467
Ttc33	NM_026213	7	1085	CAGUGCA	1091	0.0496
Klc3	NM_146182	7	1693	CAGUGCA	1699	0.0122
Gabrb1	NM_008069	7	1804	CAGUGCA	1810	0.0262
Lamp1	NM_010684	7	2125	CAGUGCA	2131	0.0471
Dtna	NM_010087	7	1677	CAGUGCA	1683	0.0153
Morn5	NM_029309	7	682	CAGUGCA	688	0.0074
Socs2	NM_007706	7	1396	CAGUGCA	1402	0.0367
Ppa2	NM_146141	7	1072	CAGUGCA	1078	0.0126
Dap3	NM_022994	7	1702	CAGUGCA	1708	0.0241
Echdc2	NM_026728	7	992	CAGUGCA	998	0.0195
Gnpat	NM_010322	7	2596	CAGUGCA	2602	0.0443
Zbtb8a	NM_028603	7	1863	CAGUGCA	1869	0.0324
Dyrk1b	NM_001037957	7	2271	CAGUGCA	2277	0.0224
Fut8	NM_016893	7	2510	CAGUGCA	2516	0.0401
Pfdn5	NM_027044	7	542	CAGUGCA	548	0.0103
2310007H09Rik	NM_029609	7	1282	CAGUGCA	1288	0.0436
AC152410.6	NM_027829	7	752	CAGUGCA	758	0.0140
4930504E06Rik	NM_133858	7	2449	CAGUGCA	2455	0.0259
Slc26a5	NM_030727	7	2612	CAGUGCA	2618	0.0296
Lipi	NM_177142	7	1404	CAGUGCA	1410	0.0306
4732473B16Rik	NM_175307	7	2278	CAGUGCA	2284	0.0498
Polr2c	NM_009090	7	960	CAGUGCA	966	0.0387
Atp6v0d1	NM_013477	7	1208	CAGUGCA	1214	0.0261
Tmem128	NM_025480	7	598	CAGUGCA	604	0.0340
Dhrs13	NM_183286	7	1488	CAGUGCA	1494	0.0332
Spg3a	NM_178628	7	2497	CAGUGCA	2503	0.0414
AC153819.5	NM_018769	7	1747	CAGUGCA	1753	0.0319
Cebpe	NM_207131	7	1221	CAGUGCA	1227	0.0107
Apol7a	NM_029419	7	1892	CAGUGCA	1898	0.0466
Wdr20b	NM_027614	7	2048	CAGUGCA	2054	0.0167
Gstm3	NM_010359	7	763	CAGUGCA	769	0.0317
Wnt16	NM_053116	7	1451	CAGUGCA	1457	0.0176
Hes1	NM_008235	7	1257	CAGUGCA	1263	0.0244
Myo1f	NM_053214	7	3508	CAGUGCA	3514	0.0242
Nup188	NM_198304	7	5324	CAGUGCA	5330	0.0232
2510039O18Rik	NM_029841	7	2186	CAGUGCA	2192	0.0416
Plk1	NM_011121	7	1967	CAGUGCA	1973	0.0172
1810022C23Rik	NM_026947	7	1050	CAGUGCA	1056	0.0190
Rps24	NM_207634	7	618	CAGUGCA	624	0.0217
Eps8l1	NM_026146	7	2368	CAGUGCA	2374	0.0190
Zc3h8	NM_020594	7	1190	CAGUGCA	1196	0.0360
Slc13a1	NM_019481	7	2041	CAGUGCA	2047	0.0408
Gnrh1	NM_008145	7	379	CAGUGCA	385	0.0094
Pnpla6	NM_015801	7	4373	CAGUGCA	4379	0.0155
Ifitm6	NM_001033632	7	460	CAGUGCA	466	0.0117
Srd5a2l	NM_020611	7	1437	CAGUGCA	1443	0.0412
Raly	NM_023130	7	1632	CAGUGCA	1638	0.0224
Ppp3ca	NM_008913	7	1958	CAGUGCA	1964	0.0332
Lefty1	NM_010094	7	1402	CAGUGCA	1408	0.0267
EG331493	NM_001033541	7	1250	CAGUGCA	1256	0.0478

Comprehensive view of miRNA predicted targets on mRNA 3' UTR region produced by miRWalk and other programs

Note : The below table displays all putative targets produced by both miRWalk and other programs i.e. contains all the putative targets of other programs (3rd party algorithms).

MicroRNA	Gene	miRanda	miRDB	miRWalk	RNA22	Targetscan	SUM
**mmu-miR-721**	**Nos2**	**1**	**0**	**0**	**0**	**1**	**2**
